# TFEB and TFE3 drive kidney cystogenesis and tumorigenesis

**DOI:** 10.15252/emmm.202216877

**Published:** 2023-03-29

**Authors:** Chiara Di Malta, Angela Zampelli, Letizia Granieri, Claudia Vilardo, Rossella De Cegli, Laura Cinque, Edoardo Nusco, Salvatore Pece, Daniela Tosoni, Francesca Sanguedolce, Nicolina Cristina Sorrentino, Maria J Merino, Deborah Nielsen, Ramaprasad Srinivasan, Mark W Ball, Christopher J Ricketts, Cathy D Vocke, Martin Lang, Baktiar Karim, Luisa Lanfrancone, Laura S Schmidt, W Marston Linehan, Andrea Ballabio

**Affiliations:** ^1^ Telethon Institute of Genetics and Medicine (TIGEM) Pozzuoli Italy; ^2^ Medical Genetics Unit, Department of Medical and Translational Science Federico II University Naples Italy; ^3^ Department of Experimental Oncology European Institute of Oncology IRCCS (IEO) Milan Italy; ^4^ Department of Pathology University of Foggia Foggia Italy; ^5^ Department of Clinical Medicine and Surgery Federico II University Naples Italy; ^6^ Laboratory of Pathology, Center for Cancer Research, National Cancer Institute National Institutes of Health Bethesda MD USA; ^7^ Urologic Oncology Branch, Center for Cancer Research National Cancer Institute, National Institutes of Health Bethesda MD USA; ^8^ Molecular Histopathology Laboratory Frederick National Laboratory for Cancer Research Frederick MD USA; ^9^ Basic Science Program, Frederick National Laboratory for Cancer Research National Cancer Institute Frederick MD USA; ^10^ Department of Molecular and Human Genetics Baylor College of Medicine Houston TX USA; ^11^ Jan and Dan Duncan Neurological Research Institute Texas Children's Hospital Houston TX USA

**Keywords:** BHD, cysts, kidney cancer, TFE3, TFEB, Cancer

## Abstract

Birt‐Hogg‐Dubé (BHD) syndrome is an inherited familial cancer syndrome characterized by the development of cutaneous lesions, pulmonary cysts, renal tumors and cysts and caused by loss‐of‐function pathogenic variants in the gene encoding the tumor‐suppressor protein folliculin (FLCN). FLCN acts as a negative regulator of TFEB and TFE3 transcription factors, master controllers of lysosomal biogenesis and autophagy, by enabling their phosphorylation by the mechanistic Target Of Rapamycin Complex 1 (mTORC1). We have previously shown that deletion of *Tfeb* rescued the renal cystic phenotype of kidney‐specific *Flcn* KO mice. Using *Flcn/Tfeb/Tfe3* double and triple KO mice, we now show that both Tfeb and Tfe3 contribute, in a differential and cooperative manner, to kidney cystogenesis. Remarkably, the analysis of BHD patient‐derived tumor samples revealed increased activation of TFEB/TFE3‐mediated transcriptional program and silencing either of the two genes rescued tumorigenesis in human BHD renal tumor cell line‐derived xenografts (CDXs). Our findings demonstrate in disease‐relevant models that both TFEB and TFE3 are key drivers of renal tumorigenesis and suggest novel therapeutic strategies based on the inhibition of these transcription factors.

## Graphical abstract

TFEB and TFE3 transcription factors are master regulators of cell metabolism. This study shows that in Birt‐Hogg‐Dubé (BHD) hereditary cancer syndrome, these factors concomitantly activate cellular catabolic and anabolic pathways, playing a key role in kidney cystogenesis and tumorigenesis.

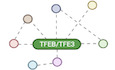

**The paper explained**

**Problem**

Birt‐Hogg‐Dubé (BHD) is a hereditary cancer syndrome caused by germline pathogenic variants of the *FLCN* gene in which affected individuals are at risk for the development of cutaneous fibrofolliculomas, pulmonary cysts and pneumothoraces, and bilateral, multifocal renal cysts and solid tumors. There is currently no effective form of therapy for BHD‐associated cutaneous or lung lesions and the only treatment for the kidney tumors is surgical removal.

**Results**

While we and others have previously shown that FLCN loss results in nuclear translocation and constitutive activation of both TFEB and TFE3, and that genetic deletion of TFEB rescues renal cystogenesis in a kidney‐specific Flcn KO mouse model, the role of TFEB/TFE3 in human BHD tumorigenesis was unknown. In the current study, we show that both Tfeb and Tfe3 contribute to murine renal cystogenesis and that human BHD renal tumors are characterized by activation of the TFEB/TFE3 transcriptional program. We also show that silencing either TFEB or TFE3 in human BHD‐associated kidney cancer cells rescues xenograft tumorigenesis. In addition, we highlight the previously unrecognized presence of an aggressive human variant of BHD‐associated RCC characterized by a pathologic phenotype reminiscent of both TFE3 papillary‐clear and TFEB eosinophilic‐clear renal cell carcinoma with cystic, cystic/solid, and solid features.

**Impact**

In this study, we highlight the critical role and clinical implications of TFEB‐ and TFE3‐driven solid, cystic and cystic/solid renal lesions. The finding that downregulation of several of the TFEB/TFE3 target genes had a significant effect on proliferation of BHD RCC tumor cells will hopefully provide the foundation for the development of an effective form of therapy for patients affected with this cancer and related disorders.

## Introduction

The *FLCN* gene encodes an evolutionarily conserved and ubiquitously expressed protein that forms heterodimers with either folliculin‐interacting protein 1 (FNIP1) or its homolog, FNIP2 (Hasumi *et al*, 2015). A main function of FLCN is to promote, through its GTPase Activating protein (GAP) activity, the activation of RagC and D GTPases, which participate in the regulation of the mechanistic Target Of Rapamycin Complex 1 (mTORC1) (Tsun *et al*, 2013; Lawrence *et al*, 2019; Shen *et al*, 2019). Mammals have four types of Rag GTPases (Rags): Rag A, B, C and D, which form obligate RagA/RagB and RagC/RagD heterodimers (Sekiguchi *et al*, 2001). The activity of the Rags depends on their nucleotide (GTP vs. GDP) binding state. In the presence of amino acids, RagA/B become GTP‐loaded and RagC/D GDP‐loaded, thus forming RagA/B‐GTP; RagC/D‐GDP heterodimers, which promote mTORC1 lysosomal recruitment and activity (Kim *et al*, 2008; Sancak *et al*, 2008). However, recent studies, including our own, have shown that whereas the GTP loading of Rag A and B is essential for the phosphorylation of all mTORC1 substrates, the GDP loading of RagC/D is dispensable for mTORC1‐mediated activity toward some substrates, such as S6K and 4E‐BP1, but is essential for the phosphorylation of MiT‐TFE transcription factors (TFs), in particular Transcription Factor EB (TFEB) and E3 (TFE3) (Wada *et al*, 2016; Lawrence *et al*, 2019; Napolitano *et al*, 2020). Because of these substrate‐specific differences, the pathway through which mTORC1 phosphorylates MiT‐TFE factors was named noncanonical mTORC1 signaling (Napolitano *et al*, 2022). The structure of the mTORC1‐TFEB‐Rag‐Ragulator protein complex was recently solved by Cryo‐EM, providing structural evidence for mTORC1 substrate specificity (Cui *et al*, 2023).

MiT‐TFE TFs can bind the same DNA motifs (Hemesath *et al*, 1994) and act as master regulators of lysosomal biogenesis and autophagy (Sardiello *et al*, 2009; Settembre *et al*, 2011; Martina *et al*, 2014). mTORC1 exerts a crucial control of MiT‐TFE TFs by inhibiting their cytoplasm‐to‐nucleus translocation through the phosphorylation of specific serine residues (Martina *et al*, 2012; Roczniak‐Ferguson *et al*, 2012; Settembre *et al*, 2012). Therefore, by activating RagC/D, FLCN enables mTORC1‐mediated phosphorylation of TFEB and TFE3, thus acting as a key negative regulator of these factors. Loss‐of‐function pathogenic variants of *FLCN* are known to cause Birt‐Hogg‐Dubé (BHD) syndrome, an autosomal dominant disorder characterized by pulmonary and renal cysts, spontaneous pneumothorax, benign cutaneous fibrofolliculomas, and an increased risk of developing kidney tumors (Schmidt & Linehan, 2015). Individuals affected with BHD inherit germline *FLCN* variants, whereas somatic “second hit” alterations are found in BHD‐associated kidney tumors (Vocke *et al*, 2005) resulting in loss of function. BHD syndrome is associated with mTORC1 hyperactivation (Baba *et al*, 2006, 2008; Chen *et al*, 2008), which paradoxically contradicts the view of FLCN as a positive regulator of mTORC1 (Petit *et al*, 2013; Tsun *et al*, 2013). We and others explained this paradox by demonstrating that loss of FLCN selectively impairs mTORC1‐mediated phosphorylation of TFEB and TFE3, resulting in their constitutive activation, without affecting the phosphorylation of other key mTORC1 substrates, such as S6K and 4EBP1 (Wada *et al*, 2016; Lawrence *et al*, 2019; Napolitano *et al*, 2020; Li *et al*, 2022). TFEB and TFE3 constitutive activation in turn promotes mTORC1 hyperactivity through a previously described mTORC1‐TFEB/TFE3‐mTORC1 feedback loop (Di Malta *et al*, 2017). We recently showed that genetic deletion of TFEB completely rescued renal cystogenesis and associated early lethality, as well as mTORC1 hyperactivation, in kidney‐specific *Flcn* KO mice (*Flcn* ^*flox/flox*^; *Ksp‐cre* ^+^), thus pointing at TFEB as a key driver of BHD associated kidney pathology (Napolitano *et al*, 2020). However, in this study, we did not explore a possible role of TFE3, whose function is known to largely overlap with that of TFEB. Furthermore, the high severity of the renal cystic phenotype of kidney‐specific *Flcn* KO mice leads to early lethality, before the development of kidney tumors, thus hampering the evaluation of the role of TFEB/TFE3 in kidney tumorigenesis. Here we show, using murine disease‐relevant models as well as BHD‐associated renal tumors, that both TFEB and TFE3 play important roles not only in the kidney cystic phenotype but also in human renal tumor formation.

## Results and Discussion

**Figure 1:**
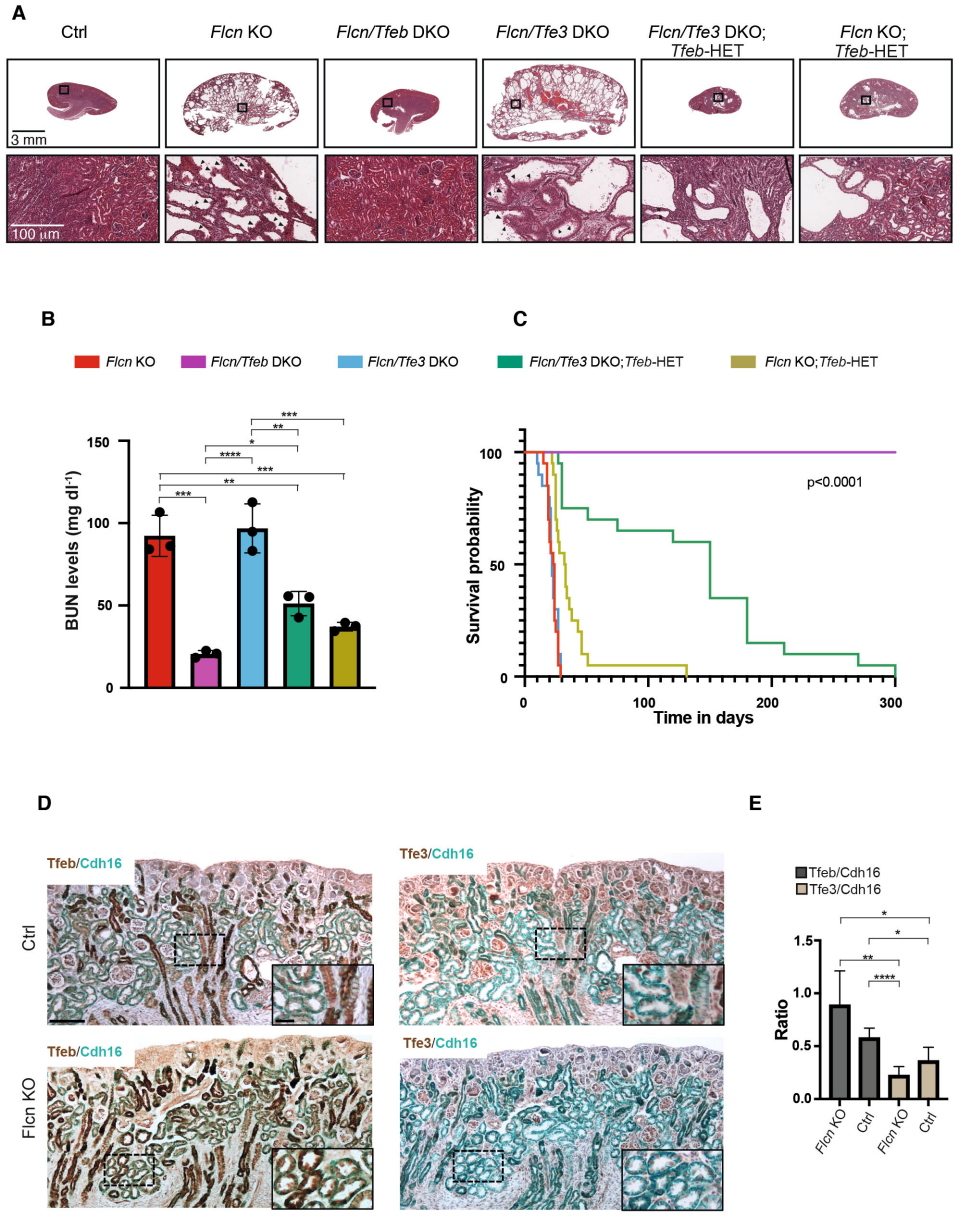
Both TFEB and TFE3 contribute to kidney pathology in *Flcn* KO mice Hematoxylin and eosin (H&E) staining of kidneys from control (*Flcn* ^*flox/flox*^), *Flcn* KO, *Flcn/Tfeb* DKO, *Flcn/Tfe3* DKO, *Flcn/Tfe3* DKO; *Tfeb*‐HET and *Flcn* KO; *Tfeb*‐HET mice at p18 (replicated three times). Scale bars, 3 mm (upper panels). Boxed areas are magnified in the bottom panels. Arrowhead indicates tubular papillary atypical hyperplasia. Scale bars, 100 μm (lower panels).Blood urea nitrogen (BUN) levels in mice of the indicated genotypes at p18 (mean ± SD, *n* = 3). One‐way ANOVA and the Tukey's HSD posthoc test (corrected for multiple comparisons) were applied. Significance for each comparison is provided in Materials and Methods.Kaplan–Meyer survival analysis of the indicated genotypes (*n* = 20 for each genotype); log‐rank test, *P*‐value < 0.0001.Representative immunohistochemical (IHC) analysis of Tfeb or Tfe3 and Cadherin‐16 (Cdh16) on adjacent kidney sections from mice of the indicated genotypes at p2. Tfeb and Tfe3 were stained in DAB, Cdh16 was stained in teal. Nuclei were stained with hematoxylin II (blue). Magnification: 10×, scale bar: 100 μm. Magnification: 20× (in inset image), scale bar: 25 μm.Quantification of TFEB or TFE3 levels relative to cadherin 16 (indicated as ratio); values represent mean ± SEM (*n* = 4 biological replicates for each genotype). A Welch's One Way ANOVA test with the Dunnett's T3 multiple comparisons test was applied. Significance for each comparison is provided in Materials and Methods. Data information: *P < 0.05; **P < 0.01; ***P < 0.001. Source data are available online for this figure.

**Figure 2:**
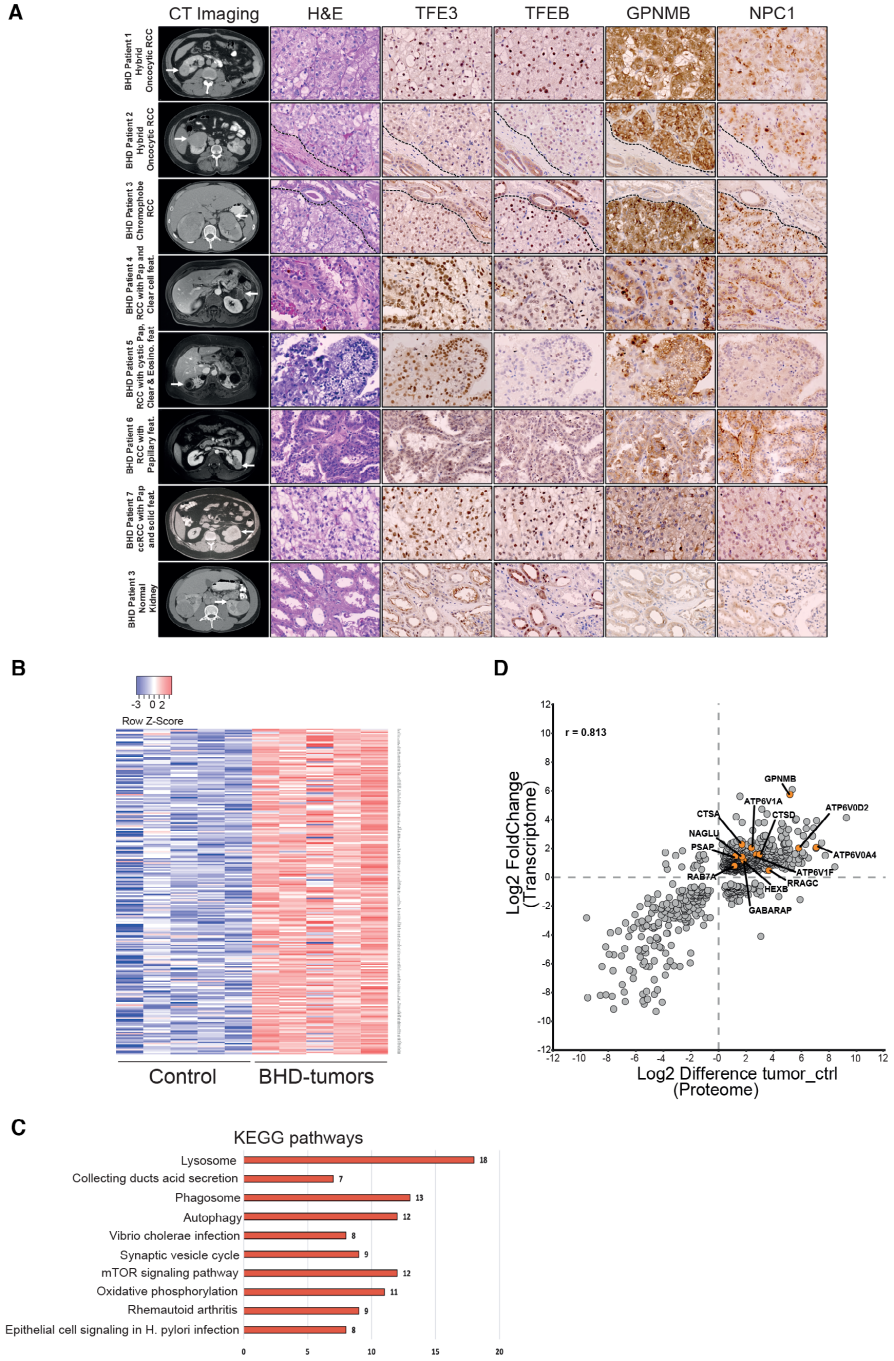
BHD kidney tumors present increased the activation of TFEB/TFE3 downstream transcriptional programs Abdominal imaging, H&E staining, and immunohistochemical staining for TFE3, TFEB, GPNMB, and NPC1 in Hybrid RCC tumors from BHD patients 1 and 2, BHD Chromophobe RCC tumor (patient 3), BHD papillary/clear RCC (patients 4, 5, and 6), a BHD patient with clear/eosinophilic/papillary RCC (patient 7, origin of the BHD RCC cell line UOK257), and an area of normal kidney from the BHD patient 3. In the rows 1–7, the arrows point to the tumor in the right or left kidney of BHD patients 1–7; in the bottom row, the arrow points to uninvolved, normal kidney in BHD patient 3.Heatmap showing validated TFEB/TFE3 target genes differentially expressed from kidney tumors of BHD patients and control kidney samples (relative to Dataset EV2). Genes are ranked from the most significantly upregulated to the less significantly upregulated in the tumor samples. Each row shows the relative expression level of a single gene. Each column shows the expression level of a single sample. Upregulated transcripts are shown in red and downregulated transcripts are shown in blue.Kegg pathways associated with genes in (B).Correlation between transcriptomic and proteomic analysis of BHD‐associated kidney tumor samples (*n* = 7 biological replicates) relative to control kidney tissues (*n* = 5 biological replicates). Source data are available online for this figure.

**Figure 3:**
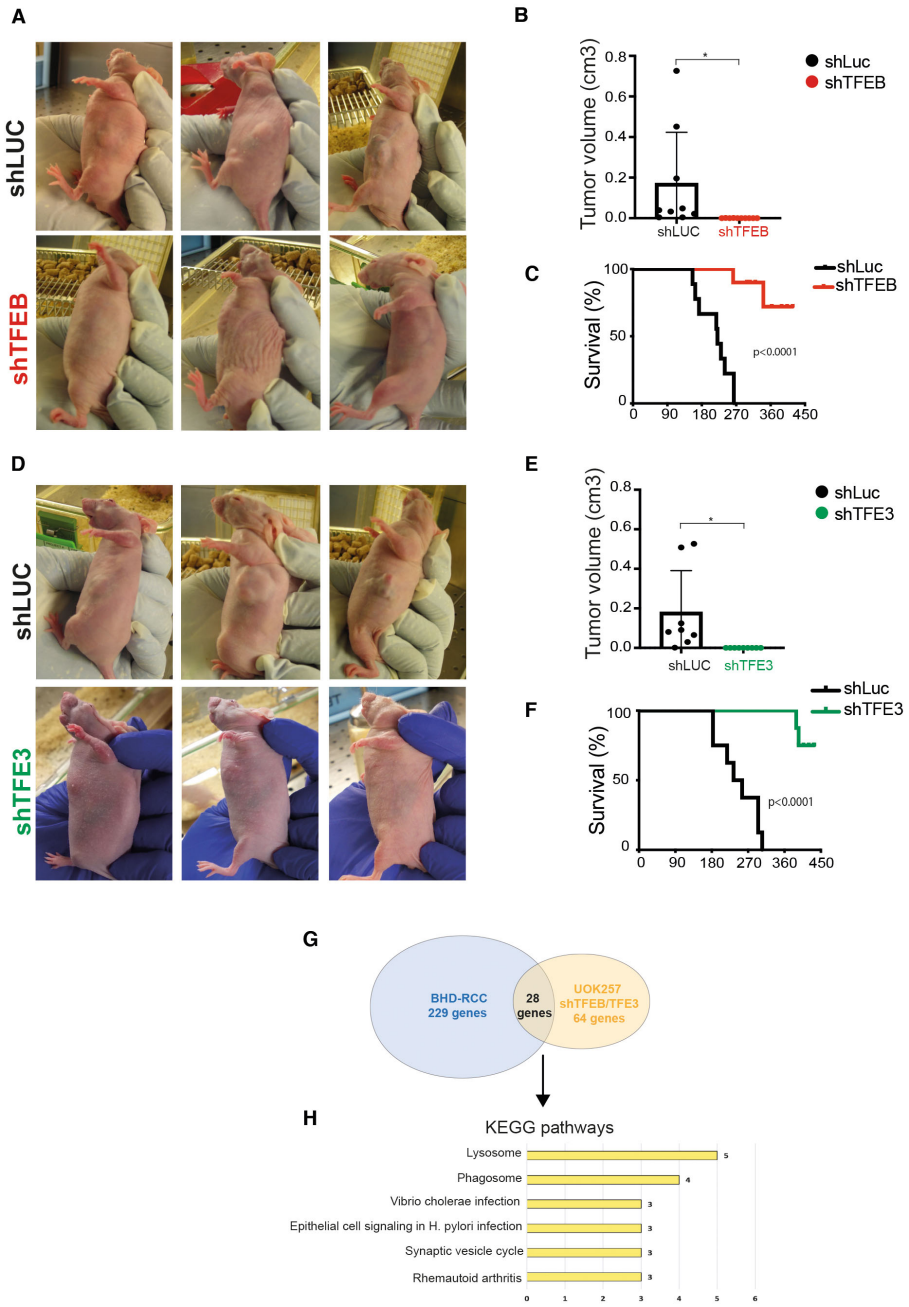
Silencing of TFEB or TFE3 inhibits the growth of BHD‐associated tumors A–F(A, D) Representative pictures of mice injected with UOK257 cells silenced for luciferase (shLuc) (A, D) or for TFEB (A) or for TFE3 (D). Pictures were taken approximately 4 months after injection. (B, E) Plots show tumor volumes in mice injected with UOK257/shLuc (*n* = 8) or UOK257/shTFEB (*n* = 10) (B) and mice injected with UOK257/shLuc (*n* = 8) or UOK257/shTFE3 (*n* = 9) (E); (mean ± SD). *P = 0.036 for plot in (B) and *P = 0.023 for plot in (E); unpaired *t*‐test. (C, F) Kaplan–Meier analysis of mice survival relative to the two experimental groups. Long‐rank test, *P* < 0.0001. The median survival time of mice injected with UOK257/shLuc is 219.5 days in (C) and 244.5 days in (F).GVenn diagram showing overlapping genes in the analyzed data sets.HKEGG pathways relative to overlapping genes in (G) (Dataset EV4). Source data are available online for this figure.

**Figure EV1:**
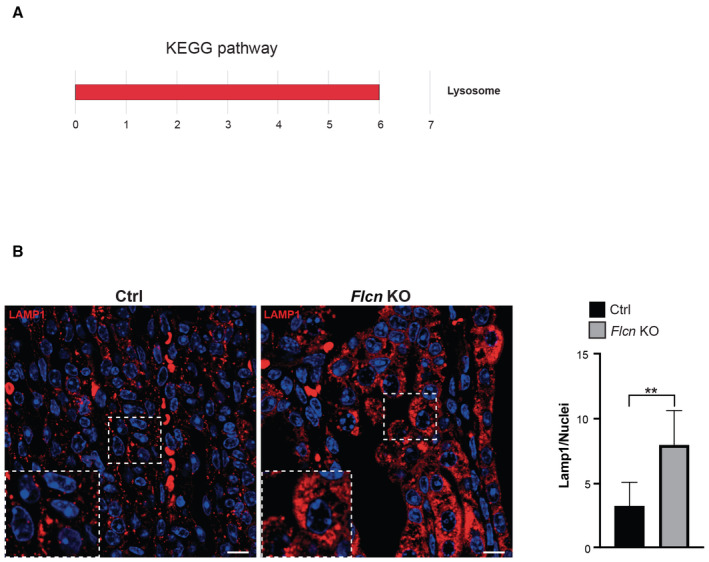
Kidney tissues from *Flcn*‐KO mice show upregulation of the lysosomal pathway Kegg pathway associated with genes significantly upregulated in kidney tissues from *Flcn* KO mice relative to control mice at the precystic stage p2 (Dataset EV1).Lamp1 immunostaining (in red) of renal tissues from control (Ctrl) and kidney‐specific *Flcn* KO (*Flcn* KO) mice. Insets show magnification of the boxed area. Nuclei were stained with DAPI (blue). Scale bar 10 μm. Bar graph shows quantification of Lamp1‐positive vesicles/nuclei. Mean ± 95% of confidence interval (*n* = 3 biological replicates). Unpaired *t*‐test **P < 0.01. Source data are available online for this figure.

**Figure EV2:**
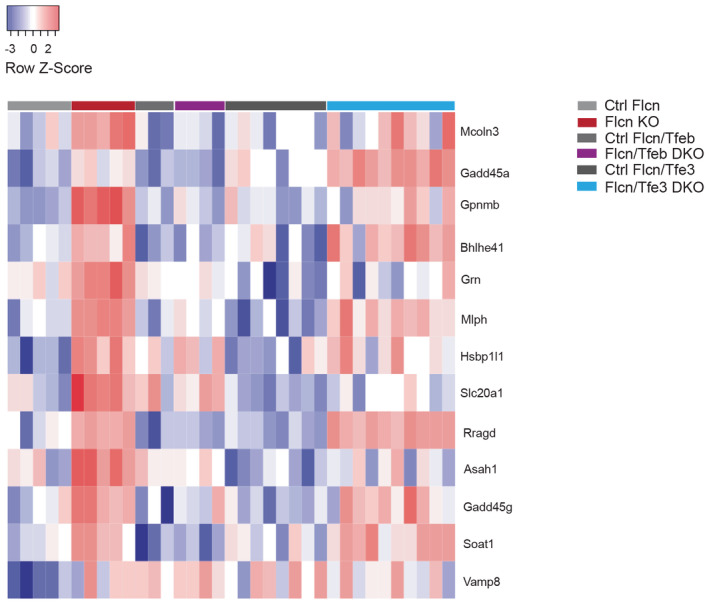
Depletion of *Tfeb* but not *Tfe3* corrects the aberrant upregulation of validated Tfeb/Tfe3 target genes in *Flcn*‐KO mice Heatmap showing validated TFEB/TFE3 target genes differentially expressed from kidney samples of the indicated murine groups (relative to Dataset EV1). Genes are ranked from the most significantly upregulated to the less significantly upregulated in the Flcn KO group. Each row shows the relative expression level of a single gene. Each column shows the expression level of a single sample. Upregulated transcripts are shown in red and downregulated transcripts are shown in blue.

**Figure EV3:**
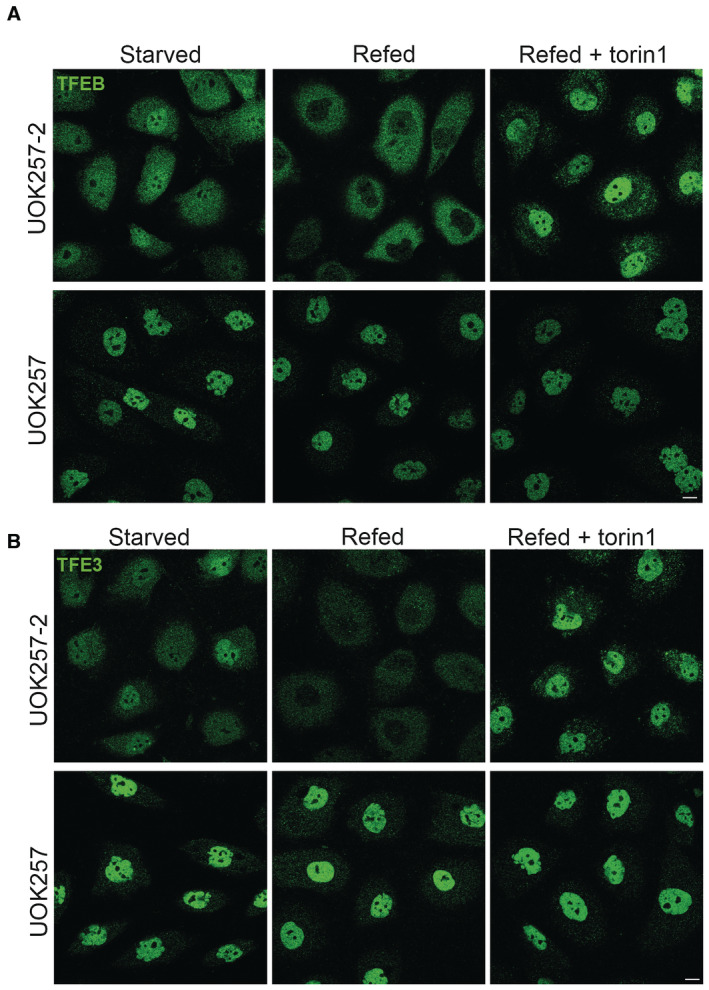
Both TFEB and TFE3 are constitutively nuclear in UOK257 cells A, BRepresentative immunofluorescence analysis of TFEB (A) or TFE3 (B) localization in BHD patient‐derived UOK257 cells and control UOK257‐2 cells, obtained by stable transfection of UOK257 cells with exogenous FLCN. Cells were deprived of amino acids for 2 h (starved) and then restimulated with amino acids (refed) for 1 h in the presence or absence of 300 nM torin1. Scale bar, 10 μm. Source data are available online for this figure.

**Figure EV4:**
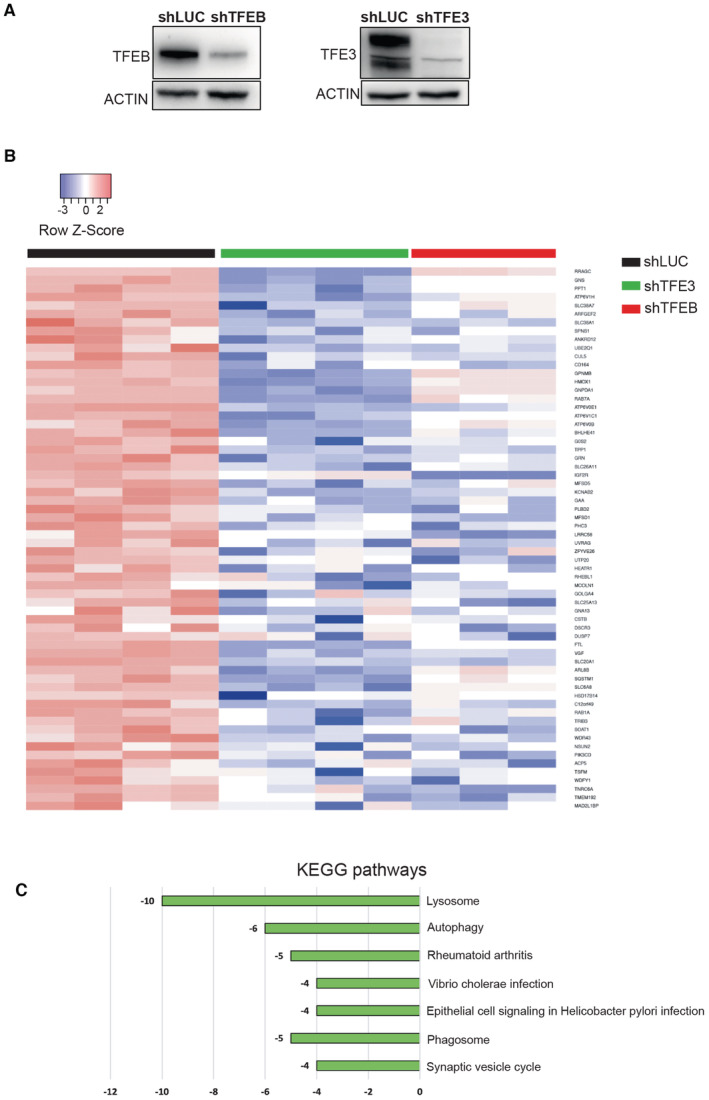
Silencing of TFEB or TFE3 abrogates the activation of target genes in UOK257 cells Immunoblot analysis of the indicated proteins in UOK257 cells silenced for luciferase (shLUC) or TFEB (shTFEB) or TFE3 (shTFE3).Heatmap showing validated TFEB target genes differentially expressed from UOK257 cells infected with the indicated shRNA (relative to Dataset EV3). Genes are ranked from the most significantly upregulated to the less significantly upregulated in the cells infected with control shRNA (shLUC). Each row shows the relative expression level of a single gene. Each column shows the expression level of a single sample. Upregulated transcripts are shown in red and downregulated transcripts are shown in blue.Kegg pathways associated with TFEB target genes significantly downregulated in (B). Source data are available online for this figure.

**Figure EV5:**
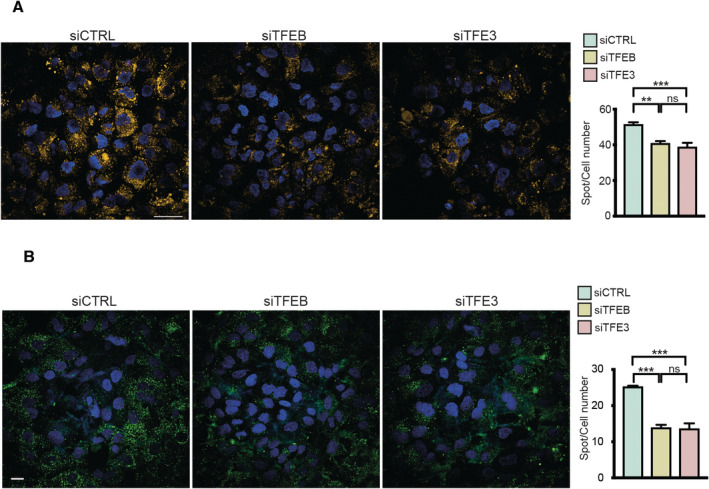
Silencing of TFEB or TFE3 significantly reduces lysosomal number and degradative ability A, BLysoTracker (A) and DQ‐BSA (B) analysis of UOK257 cells silenced for TFEB or TFE3 or scramble siRNA (CTRL). Scale bars: 50 μm in (A) and 20 μm in (B). Plots represent number of spots/cell and are expressed as mean ± SE (*n* = 3 biological replicates), ordinary one‐way ANOVA, Tukey's multiple comparisons test. Data information: **P < 0.01; ***P < 0.001; ns: not significant. Source data are available online for this figure.

As above discussed, FLCN loss is known to promote nuclear translocation and constitutive activation of both TFEB and TFE3 (Hong *et al*, 2010; Petit *et al*, 2013). In order to evaluate the role of TFE3 in the BHD renal phenotype, we crossed *Tfe3* null mice, which are viable and fertile (Steingrimsson *et al*, 2002), with kidney‐specific *Flcn* KO mice to generate *Flcn*/*Tfe3* kidney‐specific double KO (DKO) mice (*Flcn* ^*flox/flox*^; *Ksp‐cre* ^+^; *Tfe3* ^−/−^ mice) and compared their phenotype to that of kidney‐specific *Flcn* KO mice (*Flcn* ^flox/flox^; *Ksp‐cre* ^+^) and kidney‐specific *Tfeb/Flcn* DKO mice (*Flcn* ^flox/flox^; *Tfeb* ^flox/flox^; *Ksp‐cre* ^+^). We found that *Flcn/Tfe3* DKO mice were indistinguishable from *Flcn* single KO mice even at a late disease stage (post‐natal (p) day 18), showing multiple cysts expanding the cortex and medulla. Cysts were lined by a single or multiple layers of cuboidal epithelial cells (Fig 1A). In line with this observation, both *Flcn/Tfe3* DKO mice and *Flcn* single KO mice showed excessive levels of blood urea nitrogen (BUN), due to kidney dysfunction, and lethality at approximately post‐natal day 22 (Fig 1B and C). Thus, in this mouse model TFE3, unlike TFEB, appears to have a marginal role in the phenotype. Accordingly, RNA‐seq analysis showed that the expression of 139 transcripts was significantly increased in the kidneys of *Flcn* KO at a precystic stage (p2), and this upregulation was fully abrogated upon genetic depletion of TFEB but not TFE3 (Dataset EV1). By analyzing these 139 transcripts through the “Kyoto Encyclopedia of Genes and Genomes” (KEGG) database, we found that the lysosomal gene category was the only significantly enriched one, in line with the established role of these TFs in the control of lysosome biogenesis and function (Fig EV1A, Table EV1). Consistently, kidney tissues from *Flcn* KO mice showed a significant upregulation of the lysosomal marker LAMP1 (Fig EV1B). About 10% of these 139 genes scored positive on previous TFEB Chip‐seq analyses (Palmieri *et al*, 2011; Gambardella *et al*, 2020), thus representing validated target genes (Fig EV2). Considering the strong similarities between the TFEB and TFE3 transcriptional networks, we postulated that the diverse contributions of TFEB and TFE3 to kidney pathology observed in *Flcn* KO mice were due to differences in the expression levels of these transcription factors in the specific kidney cell population in which the Ksp (cadherin 16‐driven)‐Cre recombinase is expressed, which includes collecting ducts, distal tubules and thick ascending limb of Henle's loops (Shao *et al*, 2002). In line with this hypothesis, we found that TFEB expression levels are significantly higher than those of TFE3 in cadherin16‐expressing kidney cells in control (*Flcn* ^flox/flox^) mice at p2, and this difference appears even more evident in kidney‐specific *Flcn* KO mice (Fig 1D and E). This difference suggests that TFEB function is more relevant compared to that of TFE3 in these cell types at this stage and may explain why deletion of *Tfeb* rescued the phenotype of *Flcn* KO mice, whereas deletion of *Tfe3* did not. We also tested whether full deletion of *Tfe3* in the context of heterozygous deletion of *Tfeb* rescued, at least partially, the phenotype of *Flcn* mice through a “dosage effect” between these two transcription factors. At p18, *Flcn* ^flox/flox^; *Tfeb* ^flox/+^; Ksp‐cre^+^; *Tfe3* ^−/−^ (hereafter *Flcn/Tfe3*DKO; *Tfeb*‐HET mice) were indistinguishable from *Flcn* ^flox/flox^; *Tfeb* ^flox/+^; Ksp‐cre^+^ (hereafter *Flcn* KO; *Tfeb*‐HET mice) in terms of kidney appearance and function, both presenting fewer hyperplastic cysts and reduced BUN levels compared to *Flcn* KO and *Flcn/Tfe3* DKO mice (Fig 1A and B). However, *Flcn/Tfe3* DKO; *Tfeb*‐HET mice live significantly longer (mean survival 132 days) not only compared to *Flcn* KO and *Flcn/Tfe3* DKO mice but also compared to *Flcn* KO; *Tfeb*‐HET mice (mean survival 37.5 days) (Fig 1C). These results suggest that both TFEB and TFE3 play a role in the kidney phenotype associated with BHD syndrome and that *in vivo* studies of transgenic mice, which are based on the use of Ksp‐driven‐CRE recombinase may lead to an under estimation of TFE3 contribution to disease pathogenesis. To investigate the activation state of TFEB and TFE3 in BHD patient‐derived tissue samples, we performed histological examination of their cellular localization in renal tumors removed surgically at the National Cancer Institute from seven unrelated BHD patients (Fig 2A, Table EV2). While BHD‐associated kidney tumors are characterized by a variety of histological subtypes, most frequently hybrid oncocytic tumor (50%: Patients 1 and 2) and chromophobe (35%: Patient 3) renal tumors (Schmidt & Linehan, 2015), we also have BHD patients with more aggressive papillary/cystic histologic and/or clinical phenotypes (consistent with TFE3 fusion/translocation RCC) (Patients 4, 5, and 6) and those with eosinophilic/clear histology RCC reminiscent of TFE3/B translocation fusion RCC (Patient 7, origin of the UOK257 cell line). The patients with pathologic phenotypes reminiscent of TFE3/TFEB RCC tended to have a more aggressive clinical phenotype. Patients 6 and 7, for example, developed metastatic disease and patient 5 became dialysis‐dependent after surgery on both kidneys. We found increased nuclear staining of both TFEB and TFE3 in BHD hybrid and chromophobe RCC relative to control kidney tissues, as well as in the TFE3/TFEB‐like BHD tumors with tubulo papillary/eosinophilic clear RCC, as well as cystic and cystic/solid lesions, which correlated with increased staining of the Glycoprotein non‐metastatic melanoma protein B (GPNMB) and the lysosomal marker NPC intracellular cholesterol transporter 1 (NPC1), highly responsive TFEB/TFE3 transcriptional targets (Fig 2A). Transcriptomic data from renal tumors from seven additional unrelated patients (Table EV2) relative to control kidney tissues revealed that 229 out of 2,402 (about 10%) significantly upregulated genes represented validated TFEB target genes (Fig 2B, Dataset EV2). By analyzing this list of TFEB targets through KEGG database, we found that, also in this case, the lysosomal gene category was the most significantly enriched one (Fig 2C, Table EV3). Upregulation of several TFEB/TFE3 targets was also confirmed by proteomic analysis performed on the same samples (Fig 2D). These data suggest that increased activity of TFEB and TFE3 may contribute to tumor development and growth in BHD patients. To test the contribution of TFEB and TFE3 to human kidney tumorigenesis in BHD syndrome, we used the UOK257 renal cell carcinoma cell line, derived from an individual with BHD syndrome, which carries biallelic *FLCN* loss‐of‐function variants (one germline and one somatic, Fig 2A, Patient 7) (Yang *et al*, 2008). This cell line exhibits the same histological characteristics as the original tumor from which it was derived and is suitable for the generation of subcutaneous tumors in mouse xenografts, thus representing a human disease‐relevant tool for the study of the pathogenic mechanisms driving cancer growth in BHD syndrome. As expected, both TFEB and TFE3 show a constitutively nuclear localization in UOK257 cells, consistent with FLCN loss‐of‐function, whereas they are cytoplasmic in normally fed UOK257‐2 control cells, in which wild‐type FLCN is stably expressed (Baba *et al*, 2006; Hong *et al*, 2010) (Fig EV3). We infected UOK257 cells with lentiviral vectors encoding short hairpin RNAs that target TFEB or TFE3 for silencing, or with control vector (sh‐Luciferase) (Fig EV4A). Inactivation of these TFs was associated with the downregulation of 411 genes, in TFEB or TFE3‐depleted cells compared to control UOK257 (Dataset EV3). About 15% of these downregulated genes were validated TFEB targets (Palmieri *et al*, 2011; Gambardella *et al*, 2020), involved in different pathways, with the lysosomal pathway being, again, the most significantly enriched category (Fig EV4B and C, Table EV4). Accordingly, downregulation of TFEB or TFE3 significantly reduced lysosomal number and degradative capacity of UOK257 cells (Fig EV5A and B). UOK257 cells silenced for *TFEB* or *TFE3* and control UOK257 cells were then injected subcutaneously into the flanks of immunodeficient mice (8.5 × 10^5^ cells per mouse, *n* = at least eight mice per each group) and tumor growth and mouse survival monitored over time. Strikingly, we found that the single depletion of either TFEB or TFE3 was sufficient to fully abolish the growth of UOK257‐derived tumors, also resulting in prolonged survival for transplanted mice (Fig 3A–F). These results strongly indicate, in a human disease‐relevant model, that both TFEB and TFE3 drive tumorigenesis in BHD syndrome. We also compared the list of TFEB/TFE3 target genes significantly upregulated in BHD tumor samples with the list of targets downregulated in UOK257 cells silenced for TFEB or TFE3 compared to control UOK257. This comparison allowed us to identify 28 TFEB/TFE3 target genes shared between the two data sets in opposite correlation (Fig 3G and H, Dataset EV4, Table EV5). We postulate that these 28 target genes, which belong to different functional categories (Table EV6), are candidates to play a role, most likely in a cooperative manner, in BHD renal tumorigenesis. Most of these genes are involved in the regulation of cell metabolism by acting both on catabolic pathways, such as lysosomal activity and autophagy (e.g. *GNS*, *GAA*, *GRN*, *TPP1*, *Rab7*, *ARL8*, several subunits of the vATPase and *SQSTM1*) and anabolic pathways, such as mTORC1 signaling and amino acid transport (e.g., *RRAGC*, the glutamine transporters *SLC38A1* and *SLC38A7* and the creatinine transporter *SLC6A8*). These findings are consistent with previous studies showing that, once activated, TFEB and TFE3 not only promote lysosome‐mediated catabolic pathways but also increase mTORC1 activity through a feedback loop driven by transcriptional regulation of Rag C/D (Di Malta *et al*, 2017). Therefore, BHD renal tumor cells, similar to other MiT/TFE ‐dependent cancer cells, may have acquired mechanisms to escape the negative regulation of mTORC1 signaling over the autophagy process to maximize nutrient scavenging pathways for the *de novo* synthesis of the macromolecules required for cancer proliferation and growth (Perera *et al*, 2019; Napolitano *et al*, 2022). Moreover, among our selected list of candidate genes responsible of tumor growth in BHD, we also find the protumorigenic protein GPNMB, a transmembrane protein highly enriched at the plasma membrane of different types of cancer cells (e.g., melanoma, glioblastoma, and breast cancer) whose upregulation has already been reported in BHD‐RCC (Hong *et al*, 2010; Furuya *et al*, 2015), but also in translocation (t)‐RCC (Baba *et al*, 2019), MITF‐RCC (Lang *et al*, 2021) and TSC‐renal tumors (Taya & Hammes, 2018; Salles *et al*, 2022). To start assessing the potential contribution of these 28 TFEB/TFE3 target genes to the BHD renal phenotype, we downregulated each of them in UOK257 cells and then monitored cell proliferation through MTT assay. Notably, we found that the silencing of several tested genes significantly reduced cell proliferation of UOK257 cells, in line with the idea that different TFEB/TFE3 targets may contribute to tumorigenesis (Appendix Fig S1). Further analyses, especially *in vivo*, are needed to really understand whether specific TFEB/TFE3 targets are primarily responsible for tumor formation and growth. In summary, our study demonstrates that both TFEB and TFE3 are key drivers of kidney pathology in BHD syndrome and cooperate in promoting kidney cystogenesis and tumorigenesis associated with this condition. However, the relevance of each of these transcription factors in BHD syndrome kidney phenotype depends on their relative expression levels in specific renal cell populations. The evidence that depletion of only one of these transcription factors is sufficient to fully abolish tumorigenesis in *FLCN*‐deficient xenografts further supports the importance of TFEB+TFE3 gene dosage and the cooperative effect between these TFs in tumorigenesis. It will be interesting to explore whether TFEB and TFE3 have differential relevance in specific histological tumor subtypes of BHD syndrome. Furthermore, it is likely that the cooperative role played by TFEB and TFE3 in BHD tumorigenesis also contributes to the growth of other types of tumors associated with increased nuclear localization of MiT/TFE factors (e.g., PDA and invasive basal‐like breast carcinoma) (Perera *et al*, 2015; El‐Houjeiri *et al*, 2021). Future studies will also be focused on the identification of TFEB/TFE3 target genes that play critical roles in different types of malignancies. Finally, identification of molecules that are able to inhibit both of these transcription factors (e.g., by acting on upstream regulators), in an intermittent mode, may provide the foundation for the development of effective therapeutic strategies for BHD syndrome as well as other cancer‐associated diseases.

## Materials and Methods

### Materials

Reagents were obtained from the following sources: human TFEB (4240) anti‐rabbit from Cell Signaling Technology; antibody to murine TFEB from Bethyl laboratories (A303‐673A); human TFE3 anti‐rabbit from Sigma Aldrich (#SAB4200803), antibody to Cadherin 16 from Novus Biologicals (NBP1‐59248); human osteoactivin/GPNMB antigoat from R&D Systems; mouse anti‐LAMP1 was from Santa Cruz (1D4B). DMEM, RPMI, and fetal bovine serum (FBS) were from Euroclone; lentiviral plasmids sh‐RNAs, amino acids, and blasticidine were from Sigma Aldrich; Torin 1 (cat. no. 4247) from Tocris; LysoTracker DND99 (L7528) and DQ Green BSA (D12050) were from Thermo Fisher; siRNAs were purchased as SMART pool from Dharmacon.

### Cell culture, treatments, and immunofluorescence analysis

The UOK257 and the FLCN‐restored UOK257‐2 cell lines were provided by our collaborator Dr Marston Linehan; cells were cultured in DMEM high glucose (cat. no. ECM0728L, Euroclone). UOK257‐2cells were also maintained in the presence of blasticidine (2 μg/ml). UOK 257 and UOK 257‐2 cells grown on glass coverslip were rinsed twice with PBS and incubated for 2 h in amino acid‐free RPMI (cat. no. R9010‐01, US Biological) supplemented with 10% dialysed FBS. For amino acid refeeding, cells were restimulated for 1 h with 1× water‐solubilized mix of essential (cat. no.11130036, Thermo Fisher Scientific) and nonessential (cat. no. 11140035, Thermo Fisher Scientific) amino acids resuspended in aminoacid‐free RPMI supplemented with 10% dialysed FBS, plus glutamine, in presence or absence of 1 μM Torin 1. For detection of TFEB or TFE3, cells were rinsed with PBS once and then: fixed for 15 min with 4% paraformaldehyde in PBS at RT, rinsed twice with PBS, and then permeabilized with 0.1% Triton X‐100 for 5 min and then blocked with 3% bovine serum albumin in PBS + 0.02% saponin for 1 h at room temperature. Immunostainings were performed upon dilution of primary antibodies in blocking solution and overnight incubation at 4°C (for TFEB) or 2 h at RT (for TFE3), followed by three washes and secondary antibody incubation in blocking solution for 1 h at room temperature. After additional three washes, coverslips were finally mounted in VECTASHIELD mounting medium with DAPI and analyzed using LSM 800. Operators were blinded to sample identity.

### LysoTracker and DQ‐BSA experiments

LysoTracker DND99 (L7528 Thermo Fisher) was incubated at 50 nM in dark for 40 min at 37°C. DQ Green BSA (D12050 Thermo Fisher) was incubated at 10 μg/ml in dark for 15 min at 37°C. Images were acquired using the Harmony software by Opera Fenix (PerkinElmer).

For LysoTracker and DQ‐BSA experiments, 18 and 16 fields/wells were acquired, respectively. The images were subsequently analyzed using Columbus software (PerkinElmer) thanks to the support of High Content Screening facility at Tigem. Operators were blinded to sample identity.

### Mouse models

All mice used were maintained in a C57BL/6 strain background. *In vivo* studies were performed according to fully authorized animal facility, notification of the experiments to the Ministry of Health (as required by the Italian Law) Authorization n° 240/2019‐PR. The mouse line for conditional deletion of Tfeb (Tfeb^flox/flox^), or Flcn (Flcn^flox/flox^), the Tfe3^−/−^ mice and the Cdh16‐cre (Ksp‐cre) mice were previously described (Shao *et al*, 2002; Steingrimsson *et al*, 2002; Baba *et al*, 2008; Chen *et al*, 2008; Settembre *et al*, 2012). Survival curves were calculated for a period of 300 days on 20 mice for each genotype. All mice were grown in the same animal facility, all in same background (C57BL/6), mice of both sexes were used. Values were plotted by the product‐limit method of Kaplan and Meier; statistical analyses were carried out applying the log‐rank test. To analyze serum blood urea nitrogen, blood was collected from mice at p18 from retro orbital plexus. Serum blood urea nitrogen content was measured by an ammonia colorimetric assay (BioVision; cat. no. K370‐ 100) according to the manufacturer's instructions. For statistics, one‐way ANOVA and the Tukey's HSD posthoc test (corrected for multiple comparisons) were applied. Significance for each comparison was the following: *Flcn* KO versus *Flcn/Tfeb* DKO *P* = 2.27e‐05, *Flcn* KO versus *Flcn/Tfe3* DKO; *Tfeb*‐HET *P* = 2.30e‐03, *Flcn* KO versus *Flcn* KO; *Tfeb*‐HET *P* = 2.25e‐04, *Flcn/Tfeb* DKO versus *Flcn/Tfe3* DKO *P* = 1.30e‐05, *Flcn/Tfeb* DKO versus *Flcn/Tfe3* DKO; *Tfeb*‐HET *P* = 1.70e‐02, *Flcn/Tfe3* DKO versus *Flcn/Tfe3* DKO; *Tfeb*‐HET *P* = 1.04e‐03; *Flcn/Tfe3* DKO versus *Flcn* KO; *Tfeb*‐HET *P* = 1.15e‐04.

For staining of LAMP1 on kidney sections, paraffin‐embedded sections were cut at 7 μm, deparaffinized, blocked, and permeabilized in 3% (w/v) BSA, 5% goat serum in PBS + 0.3% Triton X‐100 for 3 h and then were incubated with the primary antibody overnight. Finally, sections were washed three times with 3% BSA in PBS + 0.3% Triton X‐100 and then incubated for 1 h with secondary antibodies Alexa‐Fluor‐conjugated. Sections were mounted in VECTASHIELD mounting medium with DAPI and analyzed using LSM 800 (Carl Zeiss).

### Patients and human studies

All patients were evaluated and managed in the Clinical Research Center, National Institutes of Health, Bethesda Maryland by members of the Urologic Oncology Branch, National Cancer Institute. All patient recruitment, clinical and genetic evaluation, and tissue procurement and use were approved by the Institutional Review Board of the National Cancer Institute on NCI‐ 97‐C‐0147 and/or NCI‐02‐C‐0159. Informed consent was obtained from all subjects, and the experiments were conformed to the principles set out in the WMA Declaration of Helsinki and the Department of Health and Human Services Belmont Report. The human BHD tumor histologies were read by pathologists at the National Cancer Institute, including MJM.

### Histology

For murine models, histopathological analysis was conducted on 3 μm‐thick formalin‐fixed, paraffin‐embedded kidney (FFPE) sections stained with H&E and images captured by using ImageScope (Leica‐Biosystems Nussloch GmbH). Operators were blinded to sample identity. Histology was reviewed by experienced pathologist (F.S.). For immunohistochemical analysis of TFEB, TFE3, and Cadherin 16 on kidney tissues at p2, formalin‐fixed, paraffin‐embedded kidney sections (6 μm) was analyzed by VENTANA BenchMark Ultra automated staining instrument (Ventana Medical Systems, Roche), using VENTANA reagents according to the manufacturer's instructions, thanks to the support of the Advanced Histopathology facility at TIGEM. Briefly, slides were deparaffinized using EZ Prep solution (cat # 950‐102) for 16 min at 72°C. Epitope retrieval was accomplished with CC1 solution (cat # 950‐224) at a high temperature (95°C) for a period that is suitable for a specific tissue type. Antibodies were titered with a blocking solution into user fillable dispensers for use on the automated stainer. For brightfield detection, slides were developed using the VENTANA ultra view Universal DAB detection kit (cat #760‐500) and DISCOVERY Teal HRP Kit (RUO) (cat #760‐247) according to the manufacturer's instructions. Slides were then counterstained with hematoxylin II (cat # 790‐2208) for 8 min, followed by Bluing reagent (cat # 760‐2037) for 4 min. Bright‐field sections were scanned with ZEISS Axio Scan.Z1. The whole digital slides were viewed by zen blue software. The TFEB, TFE3, and Cadherin‐16 signal was quantified by Qu‐Path software. The positive pixel count algorithm was selected and adjusted to cover each positive staining of each marker analysis using Qu‐path Software. The TFEB, TFE3, and Cadherin‐16 data were presented as positivity, which was obtained from the following formula: Ratio: percentage area of TFE3 or TFEB/percentage area of Cadherin‐16. The data were expressed as mean ± SEM. The significance of difference among the experimental groups was calculated by Welch's one‐way ANOVA test with the Dunnett's T3 multiple comparisons test. Significance for each comparison was the following: Tfeb/Cdh16 in *Flcn* KO mice versus Tfe3/Cdh16 in *Flcn* KO mice *P* = 0.0084, Tfeb/Cdh16 in *Flcn* KO mice versus Tfe3/Cdh16 in Ctrl mice *P* = 0.0320, Tfeb/Cdh16 in Ctrl mice versus Tfe3/Cdh16 in *Flcn* KO mice *P* < 0.0001, and Tfeb/Cdh16 in Ctrl mice versus Tfe3/Cdh16 in Ctrl mice *P* = 0.0367.

For histological analysis of human BHD tumor samples, H&E staining was performed on formalin‐fixed paraffin‐embedded sections by standard methods. Histology was reviewed by a pathologist experienced in evaluating kidney cancer. Immunohistochemistry for TFE3, TFEB, and GPNMB was performed as previously described (Lang *et al*, 2021).

### Xenograft experiments

Lentiviral particles from single plasmid (shLuc or shTFEB or shTFE3) were produced from HEK‐293T transfection and were added to UOK257 cells at multiplicity of infection ~4 for 16 h in standard medium supplemented with 4 μg/ml polybrene (Sigma‐Aldrich), followed by complete medium replacement. Puromycin (1 μg/ml) was added 48 h post‐infection and surviving cells harvested after 3 days. 8.5 × 10^5^ UOK257 cells were transplanted subcutaneously into the right flank of nude mice with 1:3 Matrigel Matrix HC (Corning, Cat#354248) and L15 medium (shTFEB, 10 mice per group; shTFE3, nine mice per group; shLuc, eight mice per each experimental group (shTFEB and shTFE3)). Tumor growth was monitored weekly in two dimensions using a digital caliper and mice sacrificed when the tumor reached ∼0.8 cm^3^ in volume (V), or for other reasons (i.e. age, health, and weight loss). Tumor volumes were calculated using the modified ellipsoid formula *V* = *L* × *l* ^2^/2 (*L* length; *l* width). The statistical difference in tumor volume among the groups was assessed by unpaired *t*‐test. Survival analysis was calculated with GraphPad Prism 9.0, and differences among groups were estimated by using Log‐rank test.

Nude mice were purchased from Charles River. Mice of both sexes, 6–7 weeks old, were used for experimental procedures. *In vivo* studies were performed according to fully authorized animal facility, notification of the experiments to the Ministry of Health (as required by the Italian Law) Authorization n° 509/2021‐PR.

### Cell proliferation

For MTT assay 5 mg of MTT powder was solubilized in 1 ml of PBS glucose and filtered. Cells were incubated with 100 μl of MTT solution for 1 h. At the end of the incubation time, cells treated with solubilization solution (2 ml H2O ammonia (H5NO) + 50 ml DMSO) for 10 min at 37°C to obtain a complete solubilization of the crystals. As readout, absorbance of the 96‐well plate was measured recording the optical density (OD) at 540 nm with a microplate spectrophotometer system.

### Expression analysis

For gene expression analyses, total RNA was extracted according to the manufacturer's instructions (RNeasy Mini Kit Cat No./ID: 74106 (250)). RNA extracted was precisely quantified and mixed at 50 ng/5 μl. RNA‐seq analysis was performed by Next Generation Diagnostic (NGDx s.r.l.). Libraries preparation was performed with a total of 100 ng of RNA from each sample using QuantSeq 3′mRNA‐Seq Library prep kit (Lexogen, Vienna, Austria) according to manufacturer's instructions. Amplified fragmented cDNA of 300 bp in size was sequenced in single‐end mode by NovaSeq 6000 (Illumina) with a read length of 100 bp. Illumina NovaSeq 6000 base call (BCL) files were converted in fastq file through bcl2fastq. For the analysis, the sequence reads were trimmed BBDuk (sourceforge.net/projects/bbmap/) to remove adapter sequences and low‐quality end bases (*Q* < 20). Alignment was performed with STAR 2.6.0a on the Hg38 reference provided by UCSC Genome Browser. For the mouse dataset, sequence reads were trimmed using bbduk software (https://jgi.doe.gov/data-and-tools/bbtools/bb-tools-user-guide/usage-guide/) (bbmap suite 37.31) to remove adapter sequences, poly‐A tails and low‐quality end bases (regions with average quality below) and then aligned on mm10 reference sequence using STAR. About the data processing step: the gene expression levels were determined with HTseq‐count 0.9.1. The raw expression data were normalized, analyzed, and visualized by Rosalind HyperScale architecture (OnRamp BioInformatics, Inc.). Data were analyzed by using the DAVID online tool (DAVID Bioinformatics Resources 6.8), for Gene Ontology Enrichment Analysis (GOEA), and the “Kyoto Encyclopedia of Genes and Genomes” (KEGG Pathway) analyses. The threshold for statistical significance of GOEA was FDR < 0,1, and Enrichment Score ≥ 1.5, while for the KEGG Pathway analyses was FDR < 0,1. To bioinformatically identify the CLEAR motifs in the promoter of the DEGs of interest, we used the TFEBexplorer database (De Cegli *et al*, 2022). The DEGs promoter sequence included 2,000 bp upstream and 500 bp downstream from the transcription start site (TSS) for the human or the mouse sequences. Heatmap, Venn diagram, and 3D‐Pie chart were generated using custom‐annotated scripts.

### Mass spectrometry

For proteomic analysis of BHD tumor samples relative to control kidney tissues, samples were solubilized in homogenization buffer (25 mM Tris–HCl pH 7.4, 10 mM EDTA, 10 mM EGTA, 1% NP40 supplemented with protease and phosphatase inhibitors). The soluble fractions from tissue lysates were isolated by centrifugation at 16,000 *g* for 10 min in a microfuge. All the experiments were performed in a labeling‐free setting. Proteins (30 μg) were precipitated overnight in cold acetone and peptides purified using the iST Kit (Preomics) following the company instructions. Instruments for LC MS/MS analysis consisted of a NanoLC 1200 coupled via a nano‐electrospray ionization source to the quadrupole‐based Q Exactive HF benchtop mass spectrometer (Michalski *et al*, 2011). Peptide separation was carried out according to their hydrophobicity on a home‐made chromatographic column, 75 μm ID, 8 μm tip, bed packed with Reprosil‐PUR, C18‐AQ, 1.9 μm particle size, 120 Angstrom pore size, using a binary buffer system consisting of solution A: 0.1% formic acid and B: 80% acetonitrile, 0.1% formic acid. Runs of 120 min after loading were used for proteome samples, with a constant flow rate of 300 nl/min. After sample loading, run start at 5% buffer B for 5 min, followed by a series of linear gradients, from 5% to 30% B in 90 min, then a 10 min step to reach 50% and a 5 min step to reach 95%. This last step was maintained for 10 min. Q Exactive HF settings: MS spectra were acquired using 3E6 as an AGC target, a maximal injection time of 20 ms and a 120,000 resolution at 200 *m/z*. The mass spectrometer operated in a data‐dependent Top20 mode with subsequent acquisition of higher energy collisional dissociation (HCD) fragmentation MS/MS spectra of the top 20 most intense peaks. Resolution, for MS/MS spectra, was set to 15,000 at 200 *m/z*, AGC target to 1E5, max injection time to 20 ms, and the isolation window to 1.6Th. The intensity threshold was set at 2.0 E4 and Dynamic exclusion at 30 s.

All acquired raw files were processed using MaxQuant and the implemented Andromeda search engine. For protein assignment, spectra were correlated with the UniProt Homo Sapiens including a list of common contaminants. Searches were performed with tryptic specifications and default settings for mass tolerances for MS and MS/MS spectra. Carbamidomethyl at cysteine residues was set as a fixed modification, while oxidations at methionine and acetylation at the N‐terminus were defined as variable modifications. The minimal peptide length was set to seven amino acids, and the false discovery rate for proteins and peptide‐spectrum matches to 1%. The match‐between‐run feature with a time window of 0.7 min was used. For further analysis, the Perseus software was used and first filtered for contaminants and reverse entries as well as proteins that were only identified by a modified peptide. Label‐free quantification was performed using IBAQ quantification. The values were logarithmized, grouped, and filtered for min. valid number (min. 3 in at least one group). Missing values have been replaced by random numbers that are drawn from a normal distribution. Significantly regulated proteins between conditions were determined by student *t*‐test using FDR < 0.05 as threshold. Log2 Fold‐change values for significant proteins and genes (FDR < 0.05) expressed in both the proteome and transcriptome data sets were used for correlation analysis. The Pearson correlation coefficient was used for global correlation analysis correlating the data sets. Data visualization was done in the statistical environment R.

## Author contributions

**Chiara Di Malta:** Conceptualization; formal analysis; supervision; funding acquisition; investigation; visualization; writing – original draft; project administration; writing – review and editing. **Angela Zampelli:** Investigation. **Letizia Granieri:** Formal analysis; investigation. **Claudia Vilardo:** Investigation. **Rossella De Cegli:** Formal analysis. **Laura Cinque:** Investigation. **Edoardo Nusco:** Investigation. **Salvatore Pece:** Investigation. **Daniela Tosoni:** Investigation. **Francesca Sanguedolce:** Formal analysis. **Nicolina Cristina Sorrentino:** Investigation. **Maria J Merino:** Investigation. **Deborah Nielsen:** Investigation. **Ramaprasad Srinivasan:** Investigation. **Mark W Ball:** Investigation. **Christopher J Ricketts:** Investigation. **Cathy D Vocke:** Investigation. **Martin Lang:** Investigation. **Baktiar Karim:** Investigation. **Luisa Lanfrancone:** Supervision; investigation. **Laura S Schmidt:** Resources; investigation; writing – review and editing. **W Marston Linehan:** Resources; supervision; writing – review and editing. **Andrea Ballabio:** Conceptualization; supervision; funding acquisition; writing – original draft; project administration; writing – review and editing.

## Disclosure and competing interests statement

AB is cofounder of Casma Therapeutics and advisory board member of Next Generation Diagnostics, Avilar Therapeutics and Coave Therapeutics. AB is an editorial advisory board member. This has no bearing on the editorial consideration of this article for publication.

## For more information

1. https://bhdsyndrome.org/
2. https://myrovlytistrust.org/
3. W. Marston Linehan, M.D. | Center for Cancer Research

## Supporting information



Appendix

Expanded View Figures PDF

Table EV1

Table EV2

Table EV3

Table EV4

Table EV5

Table EV6

Dataset EV1

Dataset EV2

Dataset EV3

Dataset EV4

Source Data for Expanded View

PDF+

Source Data for Figure 1

Source Data for Figure 2

Source Data for Figure 3

## Data Availability

For expression data, the whole set of results is available in the GEO database as: GSE213063: Gene expression profile of kidneys from mice at post‐natal day 2. GSE207649: Gene expression profile of kidney tumor samples from BHD patients relative to control kidney samples. GSE207650: Gene expression profile of UOK257 cells silenced for TFEB or TFE3 relative to control UOK257 silenced for luciferase. The mass spectrometry proteomics data have been deposited to the ProteomeXchange Consortium via the PRIDE [1] partner repository with the dataset identifier PXD035450.

## References

[emmm202216877-bib-0001] Baba M , Hong S‐B , Sharma N , Warren MB , Nickerson ML , Iwamatsu A , Esposito D , Gillette WK , Hopkins RF , Hartley JL *et al* (2006) Folliculin encoded by the BHD gene interacts with a binding protein, FNIP1, and AMPK, and is involved in AMPK and mTOR signaling. Proc Natl Acad Sci USA 103: 15552–15557 17028174 10.1073/pnas.0603781103 PMC1592464

[emmm202216877-bib-0002] Baba M , Furihata M , Hong S‐B , Tessarollo L , Haines DC , Southon E , Patel V , Igarashi P , Alvord WG , Leighty R *et al* (2008) Kidney‐targeted Birt‐Hogg‐Dube gene inactivation in a mouse model: Erk1/2 and Akt‐mTOR activation, cell hyperproliferation, and polycystic kidneys. J Natl Cancer Inst 100: 140–154 18182616 10.1093/jnci/djm288 PMC2704336

[emmm202216877-bib-0003] Baba M , Furuya M , Motoshima T , Lang M , Funasaki S , Ma W , Sun H‐W , Hasumi H , Huang Y , Kato I *et al* (2019) TFE3 Xp11.2 translocation renal cell carcinoma mouse model reveals novel therapeutic targets and identifies GPNMB as a diagnostic marker for human disease. Mol Cancer Res 17: 1613–1626 31043488 10.1158/1541-7786.MCR-18-1235 PMC6679785

[emmm202216877-bib-0004] Chen J , Futami K , Petillo D , Peng J , Wang P , Knol J , Li Y , Khoo S‐K , Huang D , Qian C‐N *et al* (2008) Deficiency of FLCN in mouse kidney led to development of polycystic kidneys and renal neoplasia. PLoS One 3: e3581 18974783 10.1371/journal.pone.0003581 PMC2570491

[emmm202216877-bib-0005] Cui Z , Napolitano G , de Araujo MEG , Esposito A , Monfregola J , Huber LA , Ballabio A , Hurley JH (2023) Structure of the lysosomal mTORC1–TFEB–Rag–Ragulator megacomplex. Nature 614: 572–579 36697823 10.1038/s41586-022-05652-7 PMC9931586

[emmm202216877-bib-0006] De Cegli R , Carrella D , Siciliano D , Gambardella G , Napolitano G , Di Malta C , Ballabio A , di Bernardo D (2022) TFEBexplorer: an integrated tool to study genes regulated by the stress‐responsive transcription factor EB. Autophagy Rep 1: 295–305 10.1080/27694127.2022.2097822 PMC11864625 40396031

[emmm202216877-bib-0007] Di Malta C , Siciliano D , Calcagni A , Monfregola J , Punzi S , Pastore N , Eastes AN , Davis O , De Cegli R , Zampelli A *et al* (2017) Transcriptional activation of RagD GTPase controls mTORC1 and promotes cancer growth. Science 356: 1188–1192 28619945 10.1126/science.aag2553 PMC5730647

[emmm202216877-bib-0008] El‐Houjeiri L , Biondini M , Paquette M , Kuasne H , Pacis A , Park M , Siegel PM , Pause A (2021) Folliculin impairs breast tumor growth by repressing TFE3‐dependent induction of the Warburg effect and angiogenesis. J Clin Invest 131: e144871 34779410 10.1172/JCI144871 PMC8592551

[emmm202216877-bib-0009] Furuya M , Hong S‐B , Tanaka R , Kuroda N , Nagashima Y , Nagahama K , Suyama T , Yao M , Nakatani Y (2015) Distinctive expression patterns of glycoprotein non‐metastatic B and folliculin in renal tumors in patients with Birt‐Hogg‐Dubé syndrome. Cancer Sci 106: 315–323 25594584 10.1111/cas.12601 PMC4376441

[emmm202216877-bib-0010] Gambardella G , Staiano L , Moretti MN , De Cegli R , Fagnocchi L , Di Tullio G , Polletti S , Braccia C , Armirotti A , Zippo A *et al* (2020) GADD34 is a modulator of autophagy during starvation. Sci Adv 6: eabb0205 32978159 10.1126/sciadv.abb0205 PMC7518873

[emmm202216877-bib-0011] Hasumi H , Baba M , Hasumi Y , Lang M , Huang Y , Oh HF , Matsuo M , Merino MJ , Yao M , Ito Y *et al* (2015) Folliculin‐interacting proteins Fnip1 and Fnip2 play critical roles in kidney tumor suppression in cooperation with Flcn. Proc Natl Acad Sci USA 112: E1624–E1631 25775561 10.1073/pnas.1419502112 PMC4386336

[emmm202216877-bib-0012] Hemesath TJ , Steingrímsson E , McGill G , Hansen MJ , Vaught J , Hodgkinson CA , Arnheiter H , Copeland NG , Jenkins NA , Fisher DE (1994) Microphthalmia, a critical factor in melanocyte development, defines a discrete transcription factor family. Genes Dev 8: 2770–2780 7958932 10.1101/gad.8.22.2770

[emmm202216877-bib-0013] Hong S‐B , Oh H , Valera VA , Baba M , Schmidt LS , Linehan WM (2010) Inactivation of the FLCN tumor suppressor gene induces TFE3 transcriptional activity by increasing its nuclear localization. PLoS One 5: e15793 21209915 10.1371/journal.pone.0015793 PMC3012117

[emmm202216877-bib-0014] Kim E , Goraksha‐Hicks P , Li L , Neufeld TP , Guan K‐L (2008) Regulation of TORC1 by Rag GTPases in nutrient response. Nat Cell Biol 10: 935–945 18604198 10.1038/ncb1753 PMC2711503

[emmm202216877-bib-0015] Lang M , Vocke CD , Ricketts CJ , Metwalli AR , Ball MW , Schmidt LS , Linehan WM (2021) Clinical and molecular characterization of microphthalmia‐associated transcription factor (MITF)‐related renal cell carcinoma. Urology 149: 89–97 33242557 10.1016/j.urology.2020.11.025 PMC8728951

[emmm202216877-bib-0016] Lawrence RE , Fromm SA , Fu Y , Yokom AL , Kim DJ , Thelen AM , Young LN , Lim C‐Y , Samelson AJ , Hurley JH *et al* (2019) Structural mechanism of a Rag GTPase activation checkpoint by the lysosomal folliculin complex. Science 366: 971–977 31672913 10.1126/science.aax0364 PMC6945816

[emmm202216877-bib-0017] Li K , Wada S , Gosis BS , Thorsheim C , Loose P , Arany Z (2022) Folliculin promotes substrate‐selective mTORC1 activity by activating RagC to recruit TFE3. PLoS Biol 20: e3001594 35358174 10.1371/journal.pbio.3001594 PMC9004751

[emmm202216877-bib-0018] Martina JA , Chen Y , Gucek M , Puertollano R (2012) MTORC1 functions as a transcriptional regulator of autophagy by preventing nuclear transport of TFEB. Autophagy 8: 903–914 22576015 10.4161/auto.19653 PMC3427256

[emmm202216877-bib-0019] Martina JA , Diab HI , Lishu L , Jeong‐A L , Patange S , Raben N , Puertollano R (2014) The nutrient‐responsive transcription factor TFE3 promotes autophagy, lysosomal biogenesis, and clearance of cellular debris. Sci Signal 7: ra9 24448649 10.1126/scisignal.2004754 PMC4696865

[emmm202216877-bib-0020] Michalski A , Damoc E , Hauschild J‐P , Lange O , Wieghaus A , Makarov A , Nagaraj N , Cox J , Mann M , Horning S (2011) Mass spectrometry‐based proteomics using Q Exactive, a high‐performance benchtop quadrupole Orbitrap mass spectrometer. Mol Cell Proteomics 10: M111.011015 10.1074/mcp.M111.011015 PMC3284220 21642640

[emmm202216877-bib-0021] Napolitano G , Di Malta C , Esposito A , de Araujo MEG , Pece S , Bertalot G , Matarese M , Benedetti V , Zampelli A , Stasyk T *et al* (2020) A substrate‐specific mTORC1 pathway underlies Birt‐Hogg‐Dubé syndrome. Nature 585: 597–602 32612235 10.1038/s41586-020-2444-0 PMC7610377

[emmm202216877-bib-0022] Napolitano G , Di Malta C , Ballabio A (2022) Non‐canonical mTORC1 signaling at the lysosome. Trends Cell Biol 32: 920–931 35654731 10.1016/j.tcb.2022.04.012

[emmm202216877-bib-0023] Palmieri M , Impey S , Kang H , di Ronza A , Pelz C , Sardiello M , Ballabio A (2011) Characterization of the CLEAR network reveals an integrated control of cellular clearance pathways. Hum Mol Genet 20: 3852–3866 21752829 10.1093/hmg/ddr306

[emmm202216877-bib-0024] Perera RM , Stoykova S , Nicolay BN , Ross KN , Fitamant J , Boukhali M , Lengrand J , Deshpande V , Selig MK , Ferrone CR *et al* (2015) Transcriptional control of autophagy‐lysosome function drives pancreatic cancer metabolism. Nature 524: 361–365 26168401 10.1038/nature14587 PMC5086585

[emmm202216877-bib-0025] Perera RM , Di Malta C , Ballabio A (2019) MiT/TFE family of transcription factors, lysosomes, and cancer. Annu Rev Cancer Biol 3: 203–222 31650096 10.1146/annurev-cancerbio-030518-055835 PMC6812561

[emmm202216877-bib-0026] Petit CS , Roczniak‐Ferguson A , Ferguson SM (2013) Recruitment of folliculin to lysosomes supports the amino acid‐dependent activation of Rag GTPases. J Cell Biol 202: 1107–1122 24081491 10.1083/jcb.201307084 PMC3787382

[emmm202216877-bib-0027] Roczniak‐Ferguson A , Petit CS , Froehlich F , Qian S , Ky J , Angarola B , Walther TC , Ferguson SM (2012) The transcription factor TFEB links mTORC1 signaling to transcriptional control of lysosome homeostasis. Sci Signal 5: ra42 22692423 10.1126/scisignal.2002790 PMC3437338

[emmm202216877-bib-0028] Salles DC , Asrani K , Woo J , Vidotto T , Liu HB , Vidal I , Matoso A , Netto GJ , Argani P , Lotan TL (2022) GPNMB expression identifies TSC1/2/mTOR‐associated and MiT family translocation‐driven renal neoplasms. J Pathol 257: 158–171 35072947 10.1002/path.5875 PMC9310781

[emmm202216877-bib-0029] Sancak Y , Peterson TR , Shaul YD , Lindquist RA , Thoreen CC , Bar‐Peled L , Sabatini DM (2008) The Rag GTPases bind raptor and mediate amino acid signaling to mTORC1. Science 320: 1496–1501 18497260 10.1126/science.1157535 PMC2475333

[emmm202216877-bib-0030] Sardiello M , Palmieri M , di Ronza A , Medina DL , Valenza M , Gennarino VA , Di Malta C , Donaudy F , Embrione V , Polishchuk RS *et al* (2009) A gene network regulating lysosomal biogenesis and function. Science 325: 473–477 19556463 10.1126/science.1174447

[emmm202216877-bib-0031] Schmidt LS , Linehan WM (2015) Molecular genetics and clinical features of Birt‐Hogg‐Dubé syndrome. Nat Rev Urol 12: 558–569 26334087 10.1038/nrurol.2015.206 PMC5119524

[emmm202216877-bib-0032] Sekiguchi T , Hirose E , Nakashima N , Ii M , Nishimoto T (2001) Novel G proteins, Rag C and Rag D, interact with GTP‐binding proteins, Rag A and Rag B. J Biol Chem 276: 7246–7257 11073942 10.1074/jbc.M004389200

[emmm202216877-bib-0033] Settembre C , Di Malta C , Polito VA , Garcia Arencibia M , Vetrini F , Erdin S , Erdin SU , Huynh T , Medina D , Colella P *et al* (2011) TFEB links autophagy to lysosomal biogenesis. Science 332: 1429–1433 21617040 10.1126/science.1204592 PMC3638014

[emmm202216877-bib-0034] Settembre C , Zoncu R , Medina DL , Vetrini F , Erdin S , Erdin S , Huynh T , Ferron M , Karsenty G , Vellard MC *et al* (2012) A lysosome‐to‐nucleus signalling mechanism senses and regulates the lysosome via mTOR and TFEB: self‐regulation of the lysosome via mTOR and TFEB. EMBO J 31: 1095–1108 22343943 10.1038/emboj.2012.32 PMC3298007

[emmm202216877-bib-0035] Shao X , Johnson JE , Richardson JA , Hiesberger T , Igarashi P (2002) A minimal Ksp‐cadherin promoter linked to a green fluorescent protein reporter gene exhibits tissue‐specific expression in the developing kidney and genitourinary tract. J Am Soc Nephrol 13: 1824–1836 12089378 10.1097/01.asn.0000016443.50138.cd

[emmm202216877-bib-0036] Shen K , Rogala KB , Chou H‐T , Huang RK , Yu Z , Sabatini DM (2019) Cryo‐EM structure of the human FLCN‐FNIP2‐Rag‐Ragulator complex. Cell 179: 1319–1329 31704029 10.1016/j.cell.2019.10.036 PMC7008705

[emmm202216877-bib-0037] Steingrimsson E , Tessarollo L , Pathak B , Hou L , Arnheiter H , Copeland NG , Jenkins NA (2002) Mitf and Tfe3, two members of the Mitf‐Tfe family of bHLH‐Zip transcription factors, have important but functionally redundant roles in osteoclast development. Proc Natl Acad Sci USA 99: 4477–4482 11930005 10.1073/pnas.072071099 PMC123673

[emmm202216877-bib-0038] Taya M , Hammes SR (2018) Glycoprotein non‐metastatic melanoma protein B (GPNMB) and cancer: a novel potential therapeutic target. Steroids 133: 102–107 29097143 10.1016/j.steroids.2017.10.013 PMC6166407

[emmm202216877-bib-0039] Tsun Z‐Y , Bar‐Peled L , Chantranupong L , Zoncu R , Wang T , Kim C , Spooner E , Sabatini DM (2013) The folliculin tumor suppressor is a GAP for the RagC/D GTPases that signal amino acid levels to mTORC1. Mol Cell 52: 495–505 24095279 10.1016/j.molcel.2013.09.016 PMC3867817

[emmm202216877-bib-0040] Vocke CD , Yang Y , Pavlovich CP , Schmidt LS , Nickerson ML , Torres‐Cabala CA , Merino MJ , Walther MM , Zbar B , Linehan WM (2005) High frequency of somatic frameshift BHD gene mutations in Birt‐Hogg‐Dubé‐associated renal tumors. J Natl Cancer Inst 97: 931–935 15956655 10.1093/jnci/dji154

[emmm202216877-bib-0041] Wada S , Neinast M , Jang C , Ibrahim YH , Lee G , Babu A , Li J , Hoshino A , Rowe GC , Rhee J *et al* (2016) The tumor suppressor FLCN mediates an alternate mTOR pathway to regulate browning of adipose tissue. Genes Dev 30: 2551–2564 27913603 10.1101/gad.287953.116 PMC5159669

[emmm202216877-bib-0042] Yang Y , Padilla‐Nash HM , Vira MA , Abu‐Asab MS , Val D , Worrell R , Tsokos M , Merino MJ , Pavlovich CP , Ried T *et al* (2008) The UOK 257 cell line: a novel model for studies of the human Birt‐Hogg‐Dubé gene pathway. Cancer Genet Cytogenet 180: 100–109 18206534 10.1016/j.cancergencyto.2007.10.010 PMC2440670

