# Supplementary material for: TFEB and TFE3 drive kidney cystogenesis and tumorigenesis
Source: EMBO Mol Med. 2023 Mar 29;15(5):e16877. doi: 10.15252/emmm.202216877 (PMC10165358; doi:10.15252/emmm.202216877)
Supplement: Supplementary file 1 — Appendix [file EMMM-15-e16877-s003.pdf]

Table of content:

Appendix Figure S1

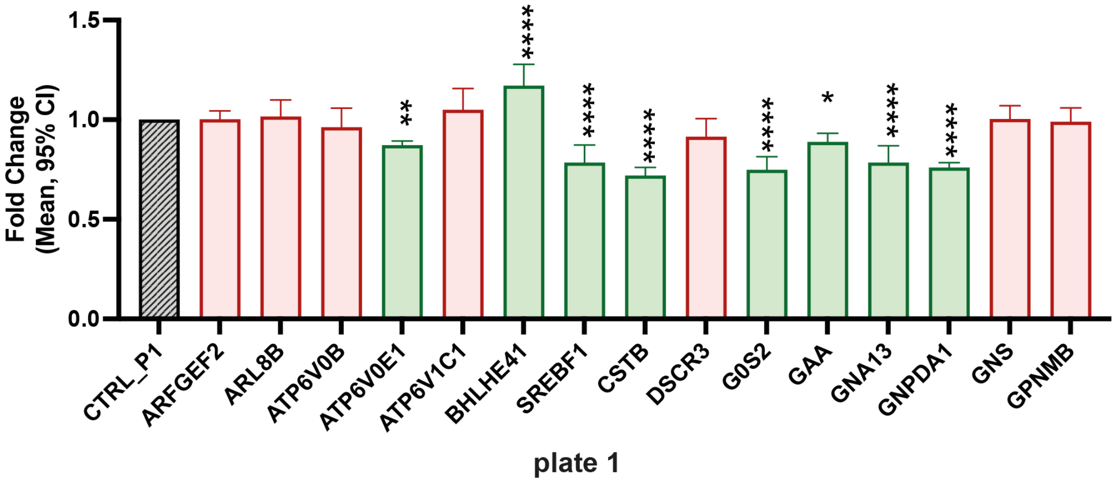

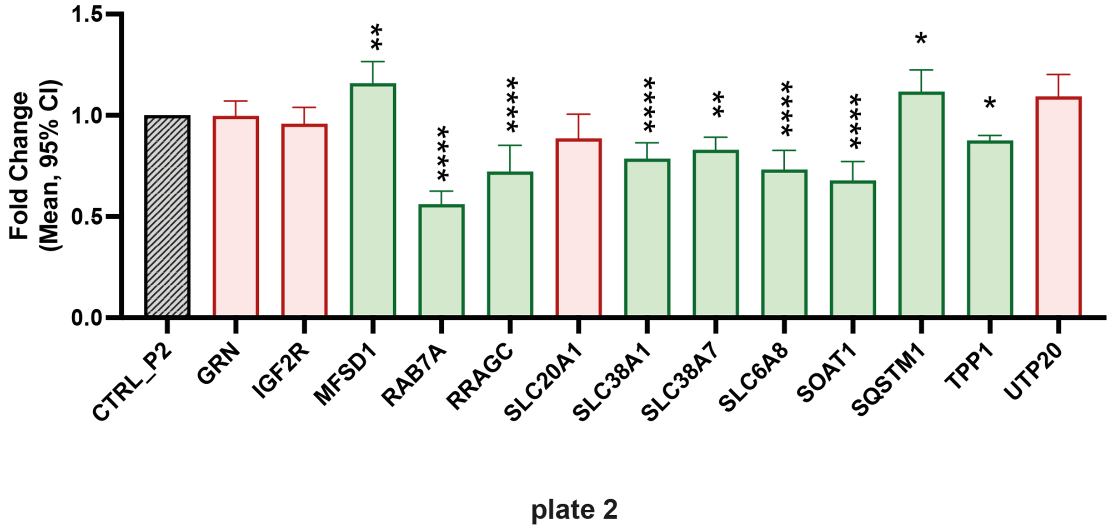

Appendix Figure S1. **Silencing of several TFEB/TFE3 target genes significantly reduces UOK257 cell proliferation.** UOK257 cells were silenced for the indicated genes and then cell proliferation index analyzed through MTT assay on two different plates (plate 1 and 2). Values are reported as fold change relative to cells transfected with scramble siRNA (CTRL). Plots represent Mean ± 95% of confidence interval (n=4), one-way ANOVA. Significant and not significant differences are shown as green and red bars, respectively. *P < 0.05; **P < 0.01; ***P < 0.001.
