# Supplementary material for: TFEB and TFE3 drive kidney cystogenesis and tumorigenesis
Source: EMBO Mol Med. 2023 Mar 29;15(5):e16877. doi: 10.15252/emmm.202216877 (PMC10165358; doi:10.15252/emmm.202216877)
Supplement: Supplementary file 2 — Expanded View Figures PDF [file EMMM-15-e16877-s011.pdf]

Expanded View Figures

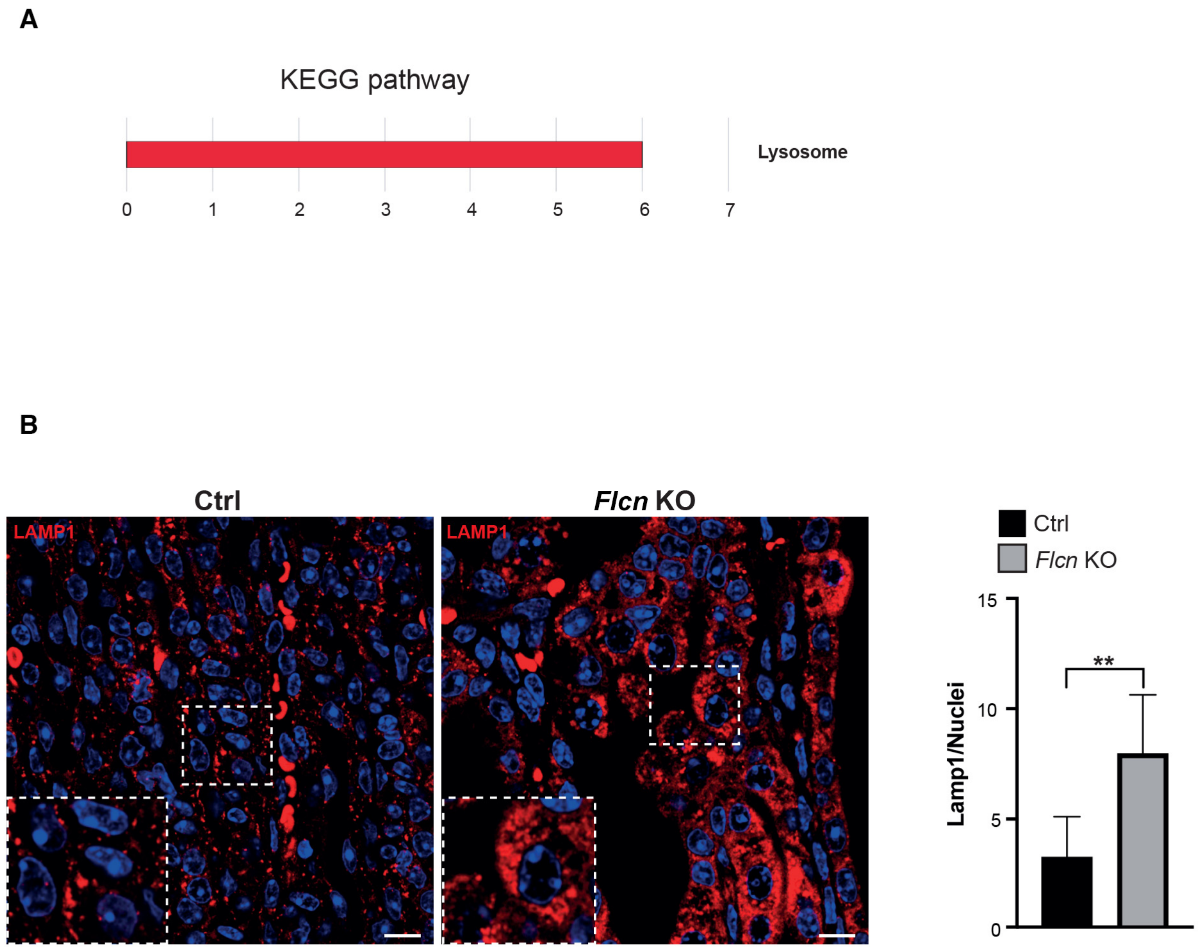

Figure EV1. Kidney tissues from *Flcn*-KO mice show upregulation of the lysosomal pathway.

A Kegg pathway associated with genes significantly upregulated in kidney tissues from *Flcn* KO mice relative to control mice at the precystic stage p2 (Dataset EV1).

B Lamp1 immunostaining (in red) of renal tissues from control (Ctrl) and kidney-specific *Flcn* KO (*Flcn* KO) mice. Insets show magnification of the boxed area. Nuclei were stained with DAPI (blue). Scale bar 10 μm. Bar graph shows quantification of Lamp1-positive vesicles/nuclei. Mean ± 95% of confidence interval (n = 3 biological replicates). Unpaired t-test $^{**}P < 0.01$.

Source data are available online for this figure.

© 2023 The Authors

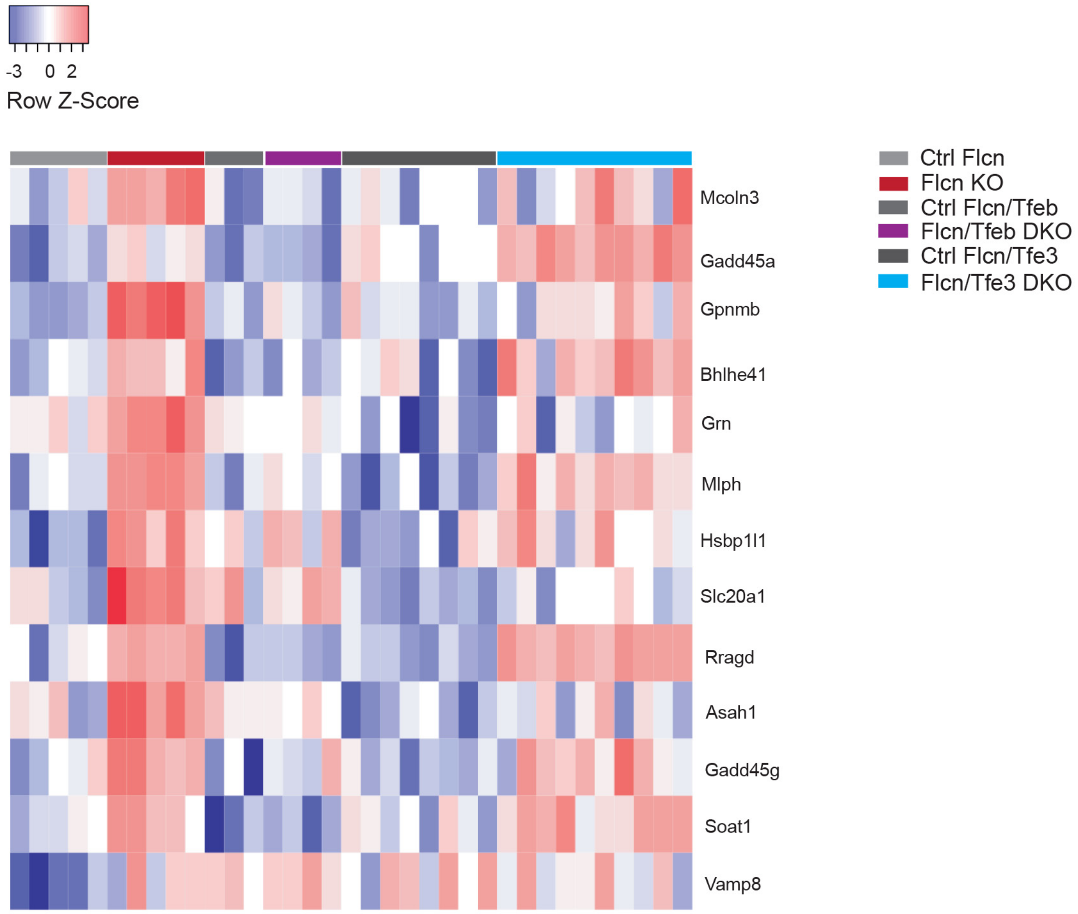

Figure EV2. Depletion of *Tfeb* but not *Tfe3* corrects the aberrant upregulation of validated Tfeb/Tfe3 target genes in *Flcn*-KO mice.

Heatmap showing validated TFEB/TFE3 target genes differentially expressed from kidney samples of the indicated murine groups (relative to Dataset EV1). Genes are ranked from the most significantly upregulated to the less significantly upregulated in the Flcn KO group. Each row shows the relative expression level of a single gene. Each column shows the expression level of a single sample. Upregulated transcripts are shown in red and downregulated transcripts are shown in blue.

 © 2023 The Authors

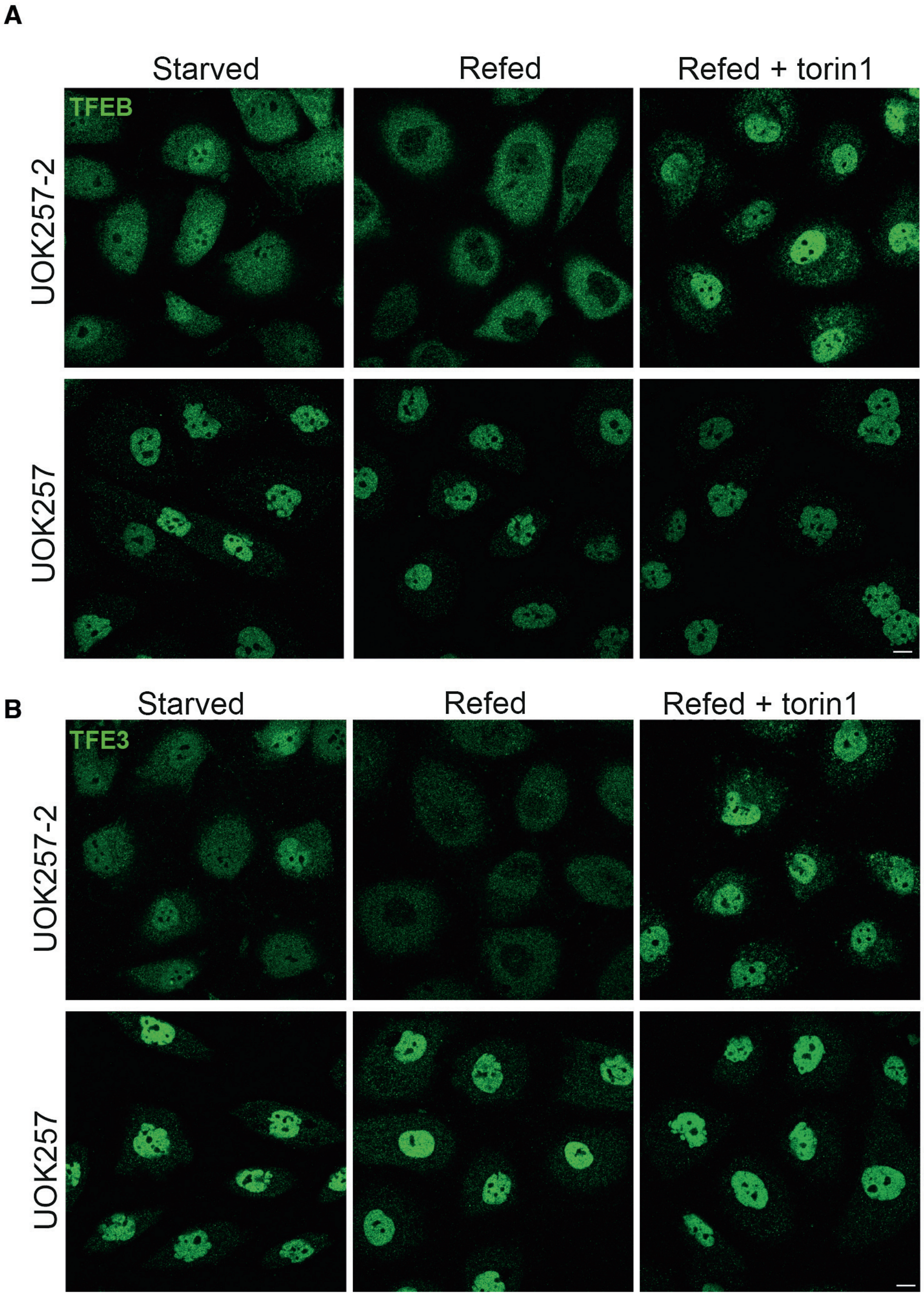

Figure EV3. Both TFEB and TFE3 are constitutively nuclear in UOK257 cells.

A, B Representative immunofluorescence analysis of TFEB (A) or TFE3 (B) localization in BHD patient-derived UOK257 cells and control UOK257-2 cells, obtained by stable transfection of UOK257 cells with exogenous FLCN. Cells were deprived of amino acids for 2 h (starved) and then restimulated with amino acids (refed) for 1 h in the presence or absence of 300 nM torin1. Scale bar, 10 µm.

Source data are available online for this figure.

© 2023 The Authors

Figure EV4. Silencing of TFEB or TFE3 abrogates the activation of target genes in UOK257 cells.

A Immunoblot analysis of the indicated proteins in UOK257 cells silenced for luciferase (shLUC) or TFEB (shTFEB) or TFE3 (shTFE3).

B Heatmap showing validated TFEB target genes differentially expressed from UOK257 cells infected with the indicated shRNA (relative to Dataset EV3). Genes are ranked from the most significantly upregulated to the less significantly upregulated in the cells infected with control shRNA (shLUC). Each row shows the relative expression level of a single gene. Each column shows the expression level of a single sample. Upregulated transcripts are shown in red and downregulated transcripts are shown in blue.

C Kegg pathways associated with TFEB target genes significantly downregulated in (B).

Source data are available online for this figure.

 © 2023 The Authors

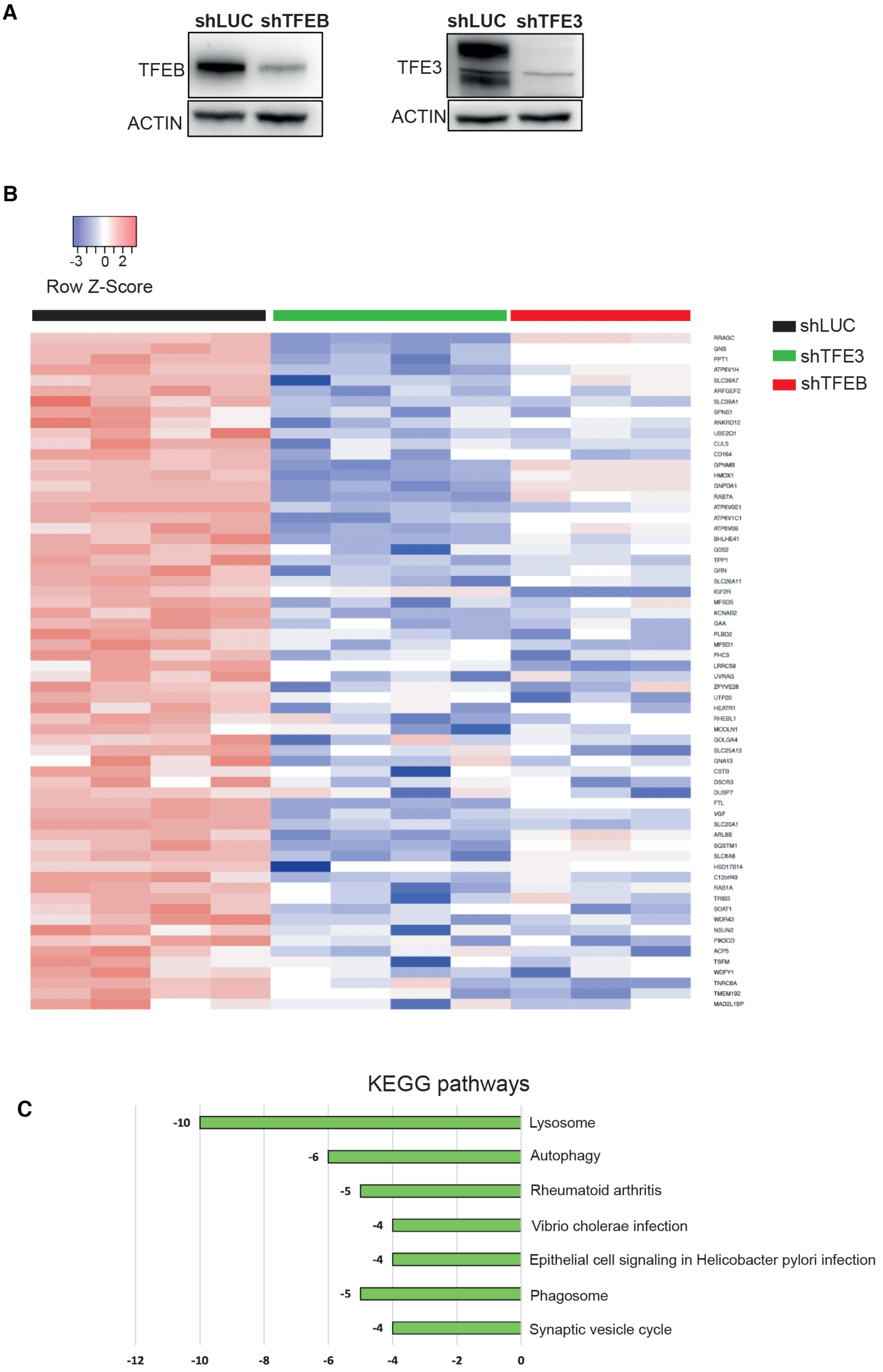
A
shLUC shTFEB
TFEB
ACTIN
shLUC shTFE3
TFE3
ACTIN
B
-3 0 2
Row Z-Score
shLUC
shTFE3
shTFEB
RRAGC
GNS
PPT1
ATP6V1H
SLC38A7
ARFGEF2
SLC38A1
SPNS1
ANKRD12
UBE2Q1
CUL5
CD164
GPNMB
HMOX1
GNPDA1
RAB7A
ATP6V0E1
ATP6V1C1
ATP6V0B
BHLHE41
G0S2
TPP1
GRN
SLC26A11
IGF2R
MFSD5
KCNAB2
GAA
PLBD2
MFSD1
PHC3
LRRC58
UVRAG
ZFYVE26
UTP20
HEATR1
RHEBL1
MCOLN1
GOLGA4
SLC25A13
GNA13
CSTB
DSCR3
DUSP7
FTL
VGF
SLC20A1
ARL8B
SQSTM1
SLC6A8
HSD17B14
C12orf49
RAB1A
TRIB3
SOAT1
WDR43
NSUN2
PIK3CD
ACP5
TSFM
WDFY1
TNRC6A
TMEM192
MAD2L1BP
C
KEGG pathways
-10 Lysosome
-6 Autophagy
-5 Rheumatoid arthritis
-4 Vibrio cholerae infection
-4 Epithelial cell signaling in Helicobacter pylori infection
-5 Phagosome
-4 Synaptic vesicle cycle
-12 -10 -8 -6 -4 -2 0

Figure EV4.

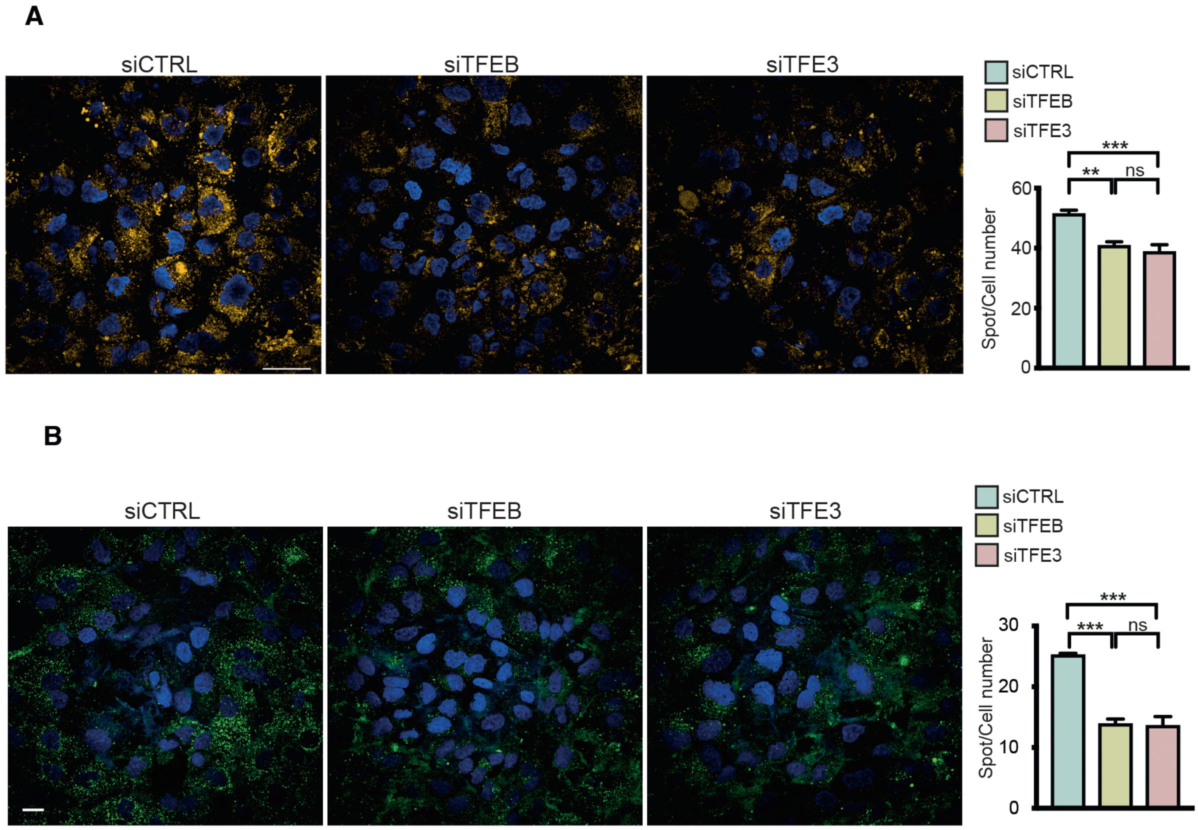

Figure EV5. Silencing of TFEB or TFE3 significantly reduces lysosomal number and degradative ability.

A, B LysoTracker (A) and DQ-BSA (B) analysis of UOK257 cells silenced for TFEB or TFE3 or scramble siRNA (CTRL). Scale bars: 50 µm in (A) and 20 µm in (B). Plots represent number of spots/cell and are expressed as mean ± SE (n = 3 biological replicates), ordinary one-way ANOVA, Tukey's multiple comparisons test.

Data information: $^{**}P < 0.01$; $^{***}P < 0.001$; ns: not significant.
Source data are available online for this figure.

 © 2023 The Authors
