# Supplementary material for: TFEB and TFE3 drive kidney cystogenesis and tumorigenesis
Source: EMBO Mol Med. 2023 Mar 29;15(5):e16877. doi: 10.15252/emmm.202216877 (PMC10165358; doi:10.15252/emmm.202216877)
Supplement: Supplementary file 4 — Table EV2 [file EMMM-15-e16877-s010.docx]

| **Patient ID** | **Age** | **Gender** | **Germline Mutation** | **Procedure** | **Histology** | **Use** |
| --- | --- | --- | --- | --- | --- | --- |
| BHD Patient 1 | 61 | Female | c.1300G>C, p.Glu434Gln | Right Partial Nx. | Multiple Hybrid RCCs | IHC Patient 1 |
| BHD Patient 2 | 60 | Male | c.1389C>G, p.Tyr463X | Right Partial Nx. | Multiple Hybrid RCCs | IHC Patient 2 |
| BHD Patient 3 | 42 | Male | c.1285dupC, p.His429ProfsX27 | Left Partial Nx. | Multiple Chromophobe RCCs | IHC Patient 3 |
| BHD Patient 4 | 73 | Female | c.632_633delCGinsC, p.Glu211AlafsX11 | Left Partial Nx. | RCC with papillary, cystic, and clear cell features | IHC Patient 4 |
| BHD Patient 5 | 67 | Female | c.503_518del16inATCAG,  p.Arg168Hisfs*28 | Right Partial Nx. | RCC with papillary, solid, clear & eosinophilic areas | IHC Patient 5 |
| BHD Patient 6 | 37 | Male | c.1285dupC, p.His429ProfsX27 | Left Partial Nx. | RCC with papillary  features | IHC Patient 6 |
| BHD Patient 7 | 45 | Male | c.1285dupC, p.His429ProfsX27 | Left Radical Nx. | Clear cell RCC with papillary and solid features | IHC Patient 7 |
| BHD Patient 8 | 47 | Female | c.250-2A>G, Splice mutation | Right Partial Nx. | Multiple Hybrid RCCs | RNAseq |
| BHD Patient 9 | 48 | Male | Partial gene deletion, Exons 1-6 | Left Partial Nx. | Multiple Hybrid RCCs | RNAseq |
| BHD Patient 10 | 55 | Male | c.889 890delGA, p.Glu297LysfsX6 | Left Partial Nx. | Multiple Hybrid RCCs | RNAseq |
| BHD Patient 11 | 38 | Male | c.1318_1334dup17, p.Leu449GlnfsX25 | Left Partial Nx. | Multiple Hybrid RCCs | RNAseq |
| BHD Patient 12 | 33 | Female | c.33C>A, p.Cys11X | Left Partial Nx. | Multiple Hybrid RCCs | RNAseq |
| BHD Patient 13 | 53 | Female | c.1599_1600delGA, p.Lys534AlafsX67 | Left Partial Nx. | Multiple Hybrid RCCs | RNAseq |
| BHD Patient 14 | 43 | Female | c. I285delC, p.His429ThrfsX39 | Left Partial Nx. | Multiple Hybrid RCCs | RNAseq |

**Table EV2. Overview of patient data**
