# Supplementary material for: TFEB and TFE3 drive kidney cystogenesis and tumorigenesis
Source: EMBO Mol Med. 2023 Mar 29;15(5):e16877. doi: 10.15252/emmm.202216877 (PMC10165358; doi:10.15252/emmm.202216877)
Supplement: Supplementary file 7 — Table EV5 [file EMMM-15-e16877-s009.docx]

| **Gene** | **Protein** |
| --- | --- |
| ARFGEF2 | Brefeldin A-inhibited guanine nucleotide-exchange protein 2 |
| ARL8B | ADP-ribosylation factor-like protein 8B |
| ATP6V0B | V-type proton ATPase 21 kDa proteolipid subunit c |
| ATP6V0E1 | V-type proton ATPase subunit e 1 |
| ATP6V1C1 | V-type proton ATPase subunit C 1 |
| BHLHE41 | Class E basic helix-loop-helix protein 41 |
| SPRING1 | SREBP regulating gene protein |
| CSTB | Cystatin-B |
| VPS26C | Vacuolar protein sorting-associated protein 26C |
| G0S2 | G0/G1 switch protein 2 |
| GAA | Lysosomal alpha-glucosidase |
| GNA13 | Guanine nucleotide-binding protein subunit alpha-13 |
| GNPDA1 | Glucosamine-6-phosphate isomerase 1 |
| GNS | N-acetylglucosamine-6-sulfatase |
| GPNMB | Transmembrane glycoprotein NMB |
| GRN | Progranulin |
| IGF2R | Cation-independent mannose-6-phosphate receptor |
| MFSD1 | Major facilitator superfamily domain-containing protein 1 |
| RAB7A | Ras-related protein Rab-7a |
| RRAGC | Ras-related GTP-binding protein C |
| SLC20A1 | Sodium-dependent phosphate transporter 1 |
| SLC38A1 | Sodium-coupled neutral amino acid transporter 1 |
| SLC38A7 | Putative sodium-coupled neutral amino acid transporter 7 |
| SLC6A8 | Sodium- and chloride-dependent creatine transporter 1 |
| SOAT1 | Sterol O-acyltransferase 1 |
| SQSTM1 | Sequestosome-1 |
| TPP1 | Tripeptidyl-peptidase 1 |
| UTP20 | Small subunit processome component 20 homolog |

**Table EV5. List of candidate genes responsible for tumor growth in BHD**
