# Supplementary material for: TFEB and TFE3 drive kidney cystogenesis and tumorigenesis
Source: EMBO Mol Med. 2023 Mar 29;15(5):e16877. doi: 10.15252/emmm.202216877 (PMC10165358; doi:10.15252/emmm.202216877)
Supplement: Supplementary file 13 — Source Data for Expanded View [file EMMM-15-e16877-s012.zip › Source Data for Expanded View and Appendix figures/Fig EV4/EV4A/WBs.pptx]

## Slide 1
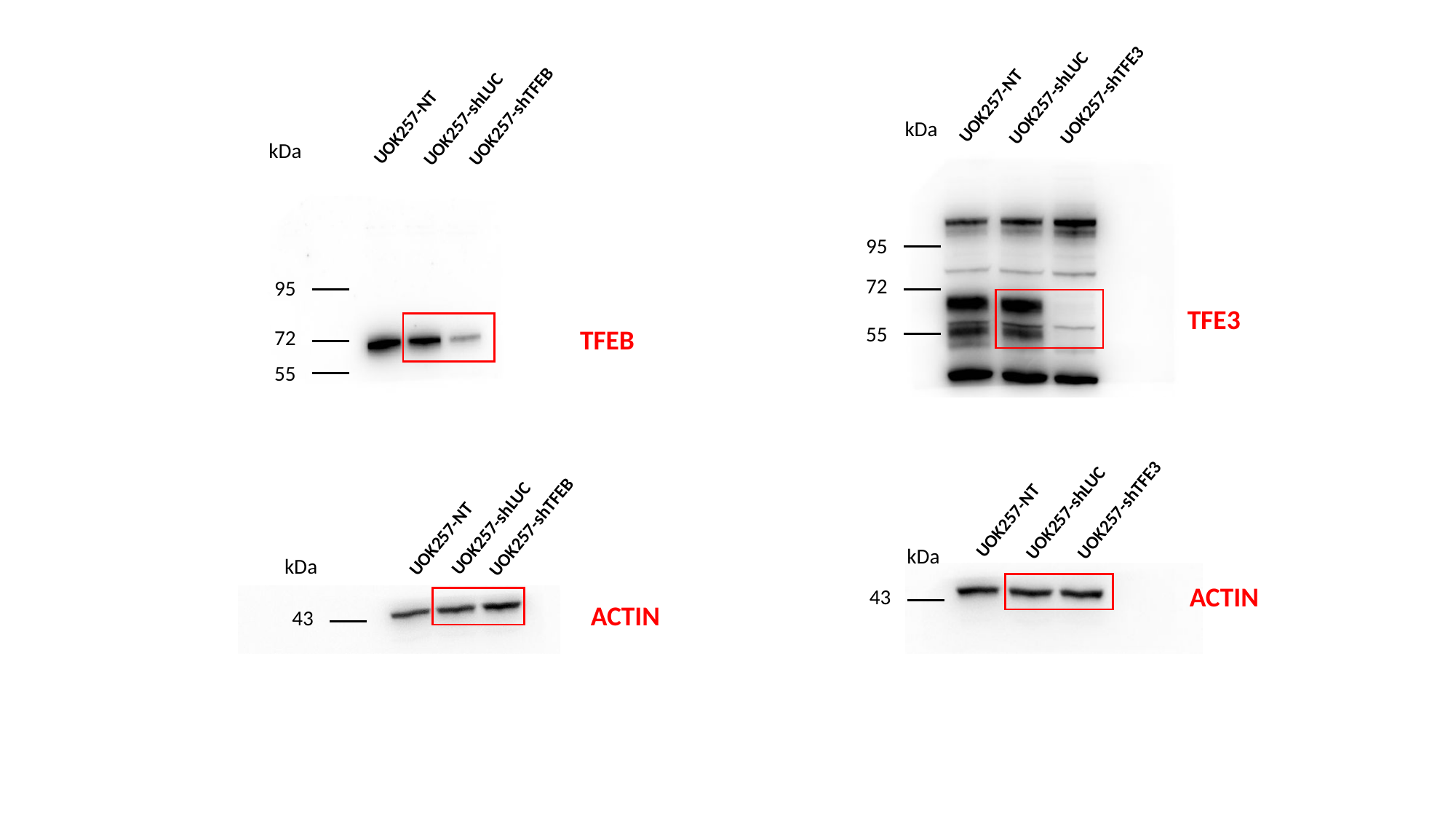

UOK257-NT
UOK257-shTFE3
UOK257-shLUC
UOK257-NT
UOK257-shTFEB
UOK257-shLUC
kDa
kDa
95
72
95
TFE3
55
TFEB
72
55
UOK257-shLUC
UOK257-NT
UOK257-shTFEB
kDa
ACTIN
43
UOK257-NT
UOK257-shTFE3
UOK257-shLUC
kDa
ACTIN
43
kDa
