# Supplementary figures and images for: TFEB and TFE3 drive kidney cystogenesis and tumorigenesis
Source: EMBO Mol Med. 2023 Mar 29;15(5):e16877. doi: 10.15252/emmm.202216877 (PMC10165358; doi:10.15252/emmm.202216877)

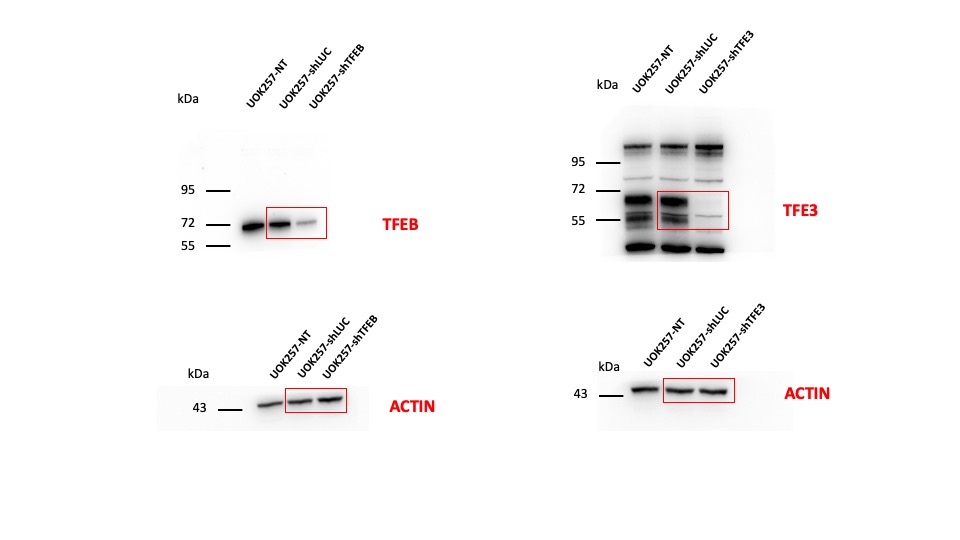

Supplement: Supplementary file 13 — Source Data for Expanded View [file EMMM-15-e16877-s012.zip › Source Data for Expanded View and Appendix figures/Fig EV4/EV4A/WBs.jpeg]

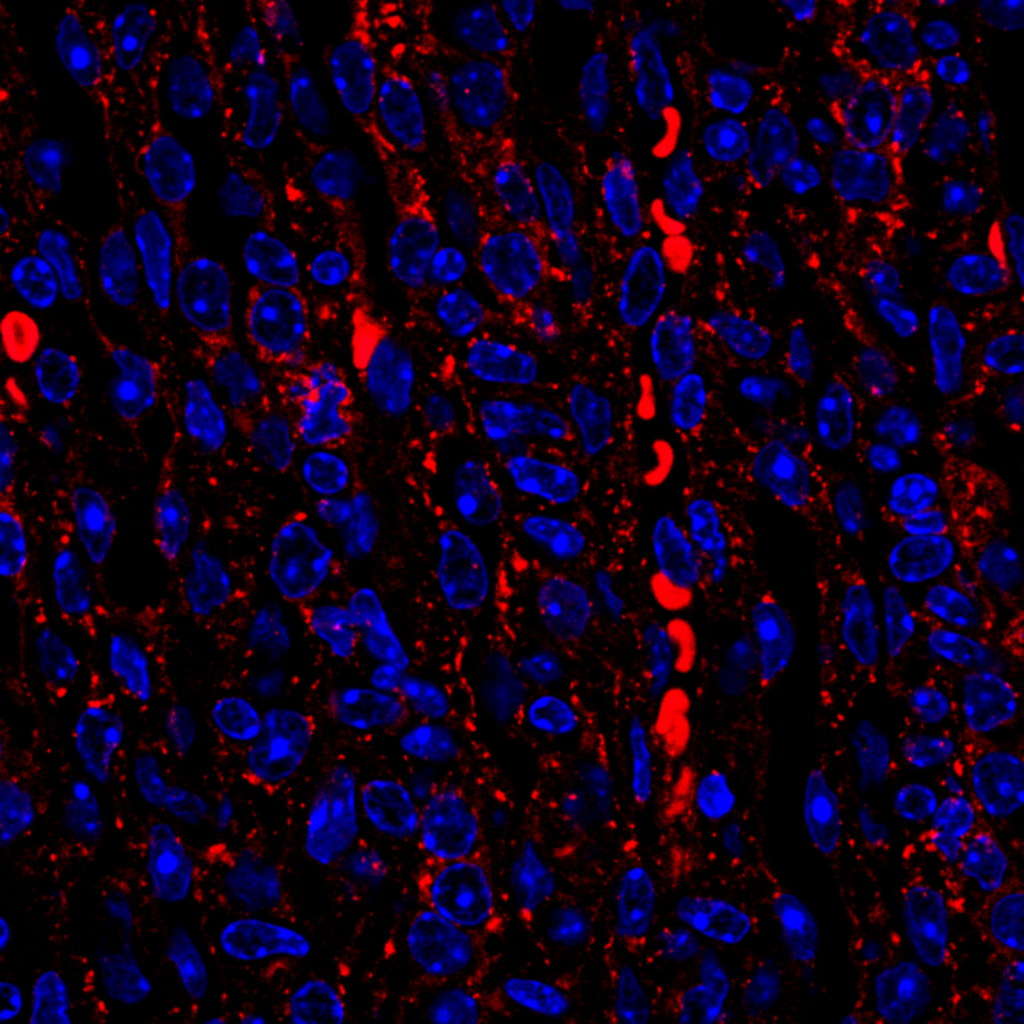

Supplement: Supplementary file 13 — Source Data for Expanded View [file EMMM-15-e16877-s012.zip › Source Data for Expanded View and Appendix figures/Fig EV1/EV1B/TIFF images/Ctrl.tif]

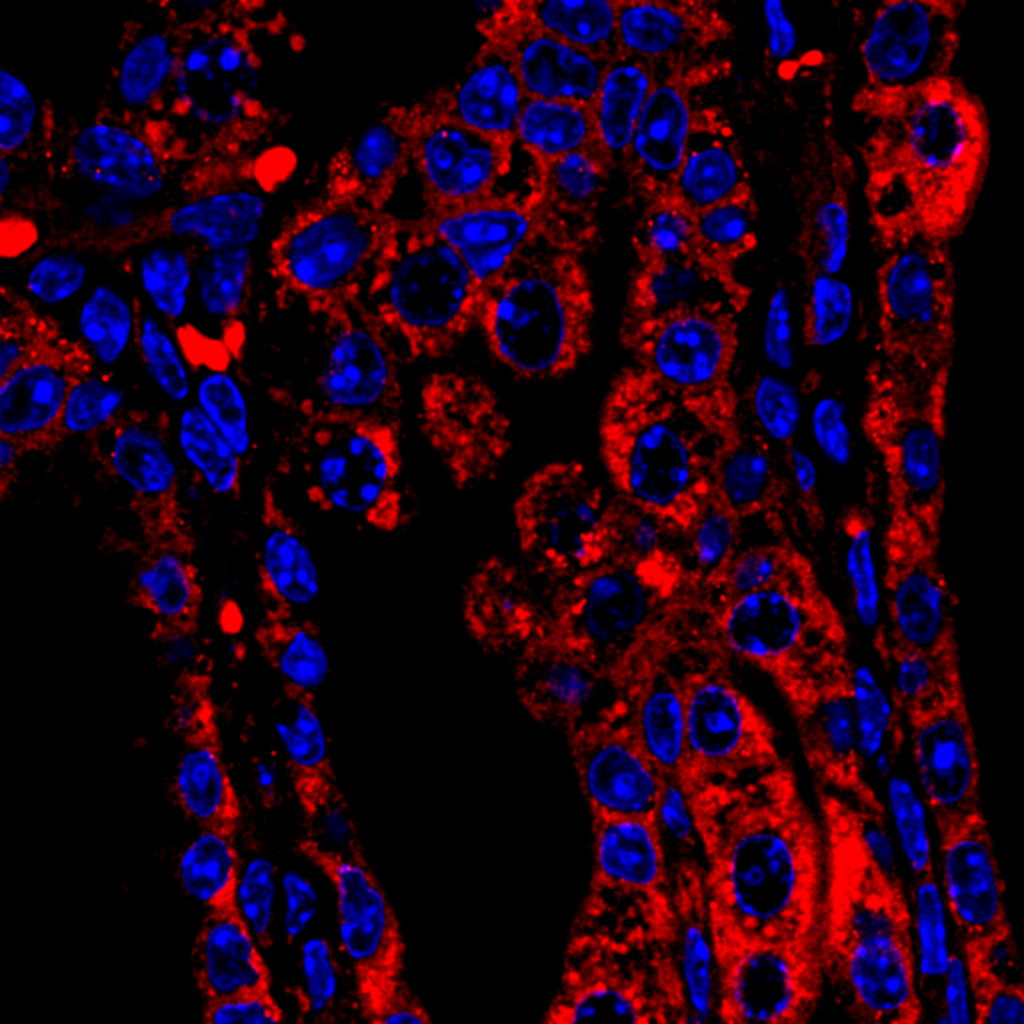

Supplement: Supplementary file 13 — Source Data for Expanded View [file EMMM-15-e16877-s012.zip › Source Data for Expanded View and Appendix figures/Fig EV1/EV1B/TIFF images/Flcn KO.tif]

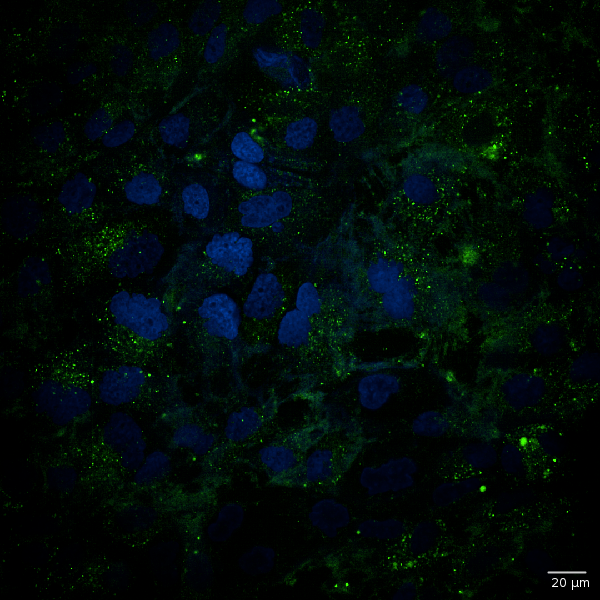

Supplement: Supplementary file 13 — Source Data for Expanded View [file EMMM-15-e16877-s012.zip › Source Data for Expanded View and Appendix figures/Fig EV5/EV5B/TIFF imges/siTFE3.png]

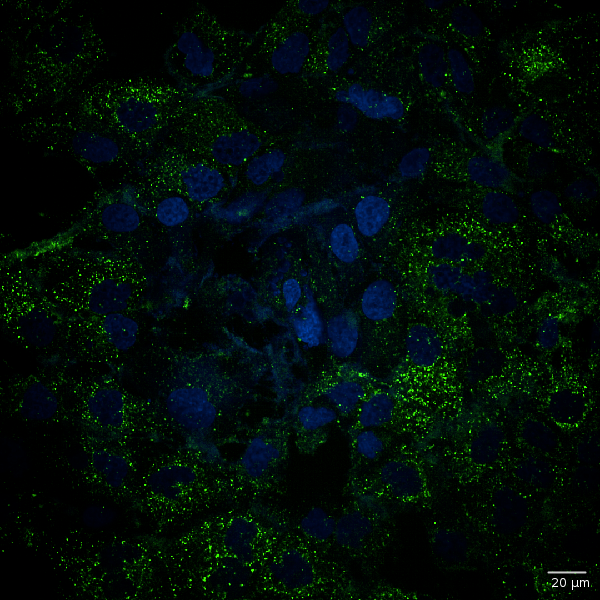

Supplement: Supplementary file 13 — Source Data for Expanded View [file EMMM-15-e16877-s012.zip › Source Data for Expanded View and Appendix figures/Fig EV5/EV5B/TIFF imges/siCTRL.png]

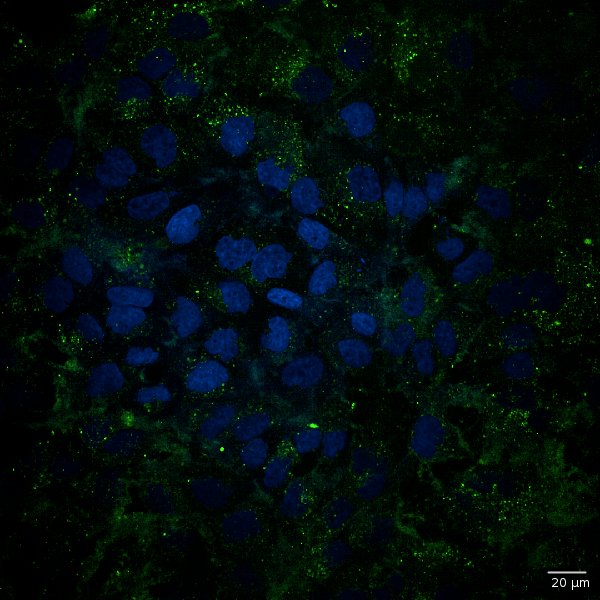

Supplement: Supplementary file 13 — Source Data for Expanded View [file EMMM-15-e16877-s012.zip › Source Data for Expanded View and Appendix figures/Fig EV5/EV5B/TIFF imges/siTFEB.png]

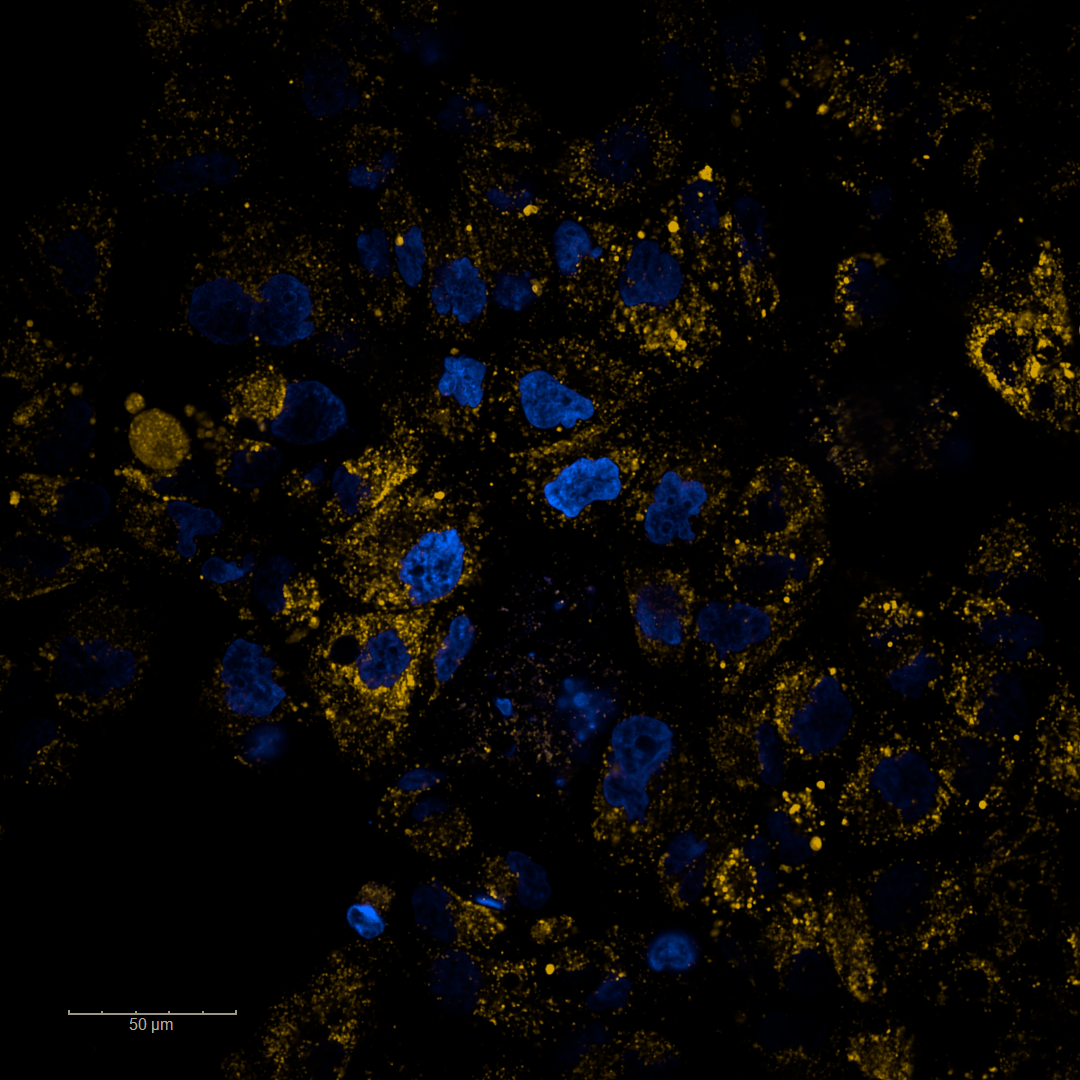

Supplement: Supplementary file 13 — Source Data for Expanded View [file EMMM-15-e16877-s012.zip › Source Data for Expanded View and Appendix figures/Fig EV5/EV5A/TIFF imges/siTFE3.png]

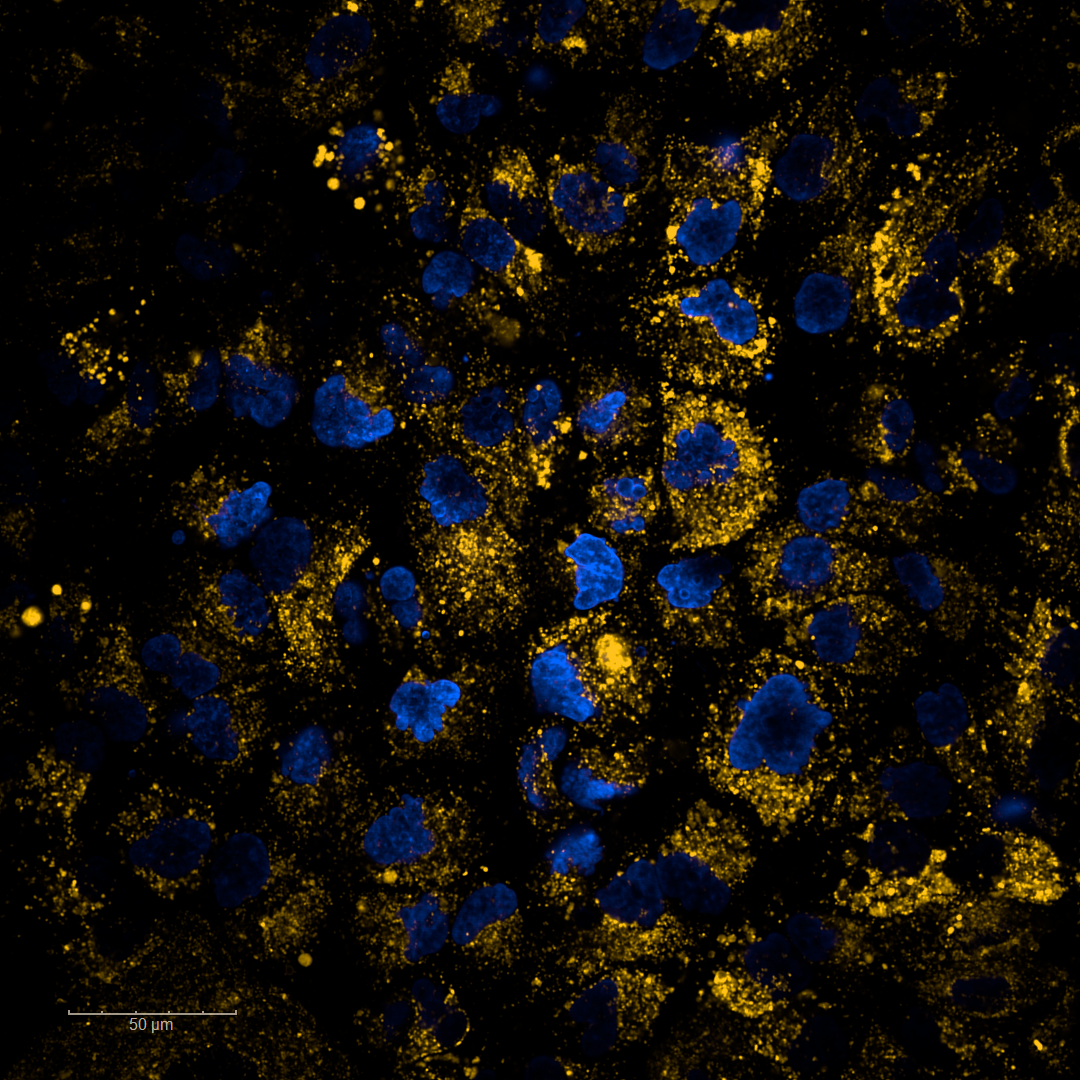

Supplement: Supplementary file 13 — Source Data for Expanded View [file EMMM-15-e16877-s012.zip › Source Data for Expanded View and Appendix figures/Fig EV5/EV5A/TIFF imges/siCTRL.png]

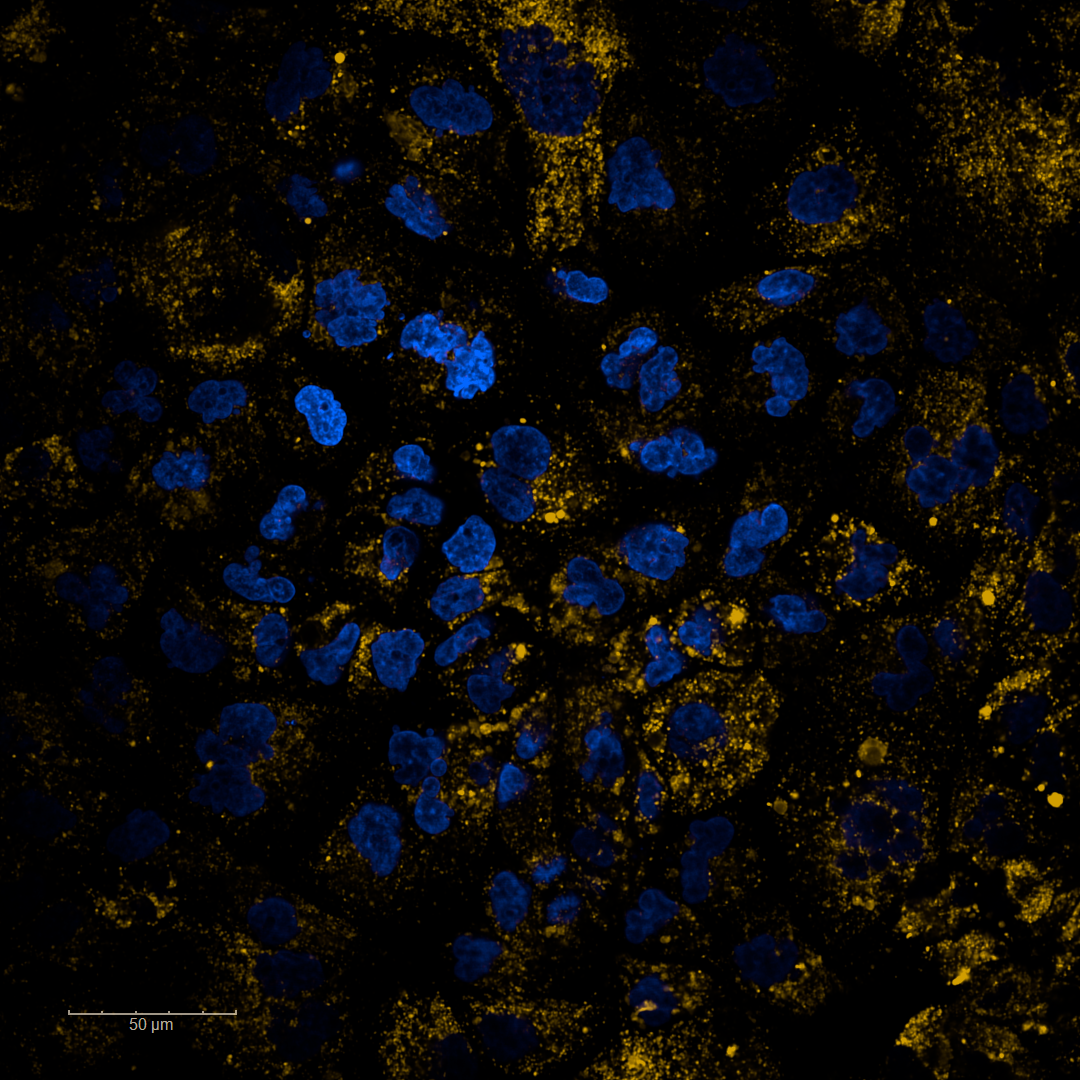

Supplement: Supplementary file 13 — Source Data for Expanded View [file EMMM-15-e16877-s012.zip › Source Data for Expanded View and Appendix figures/Fig EV5/EV5A/TIFF imges/siTFEB.png]

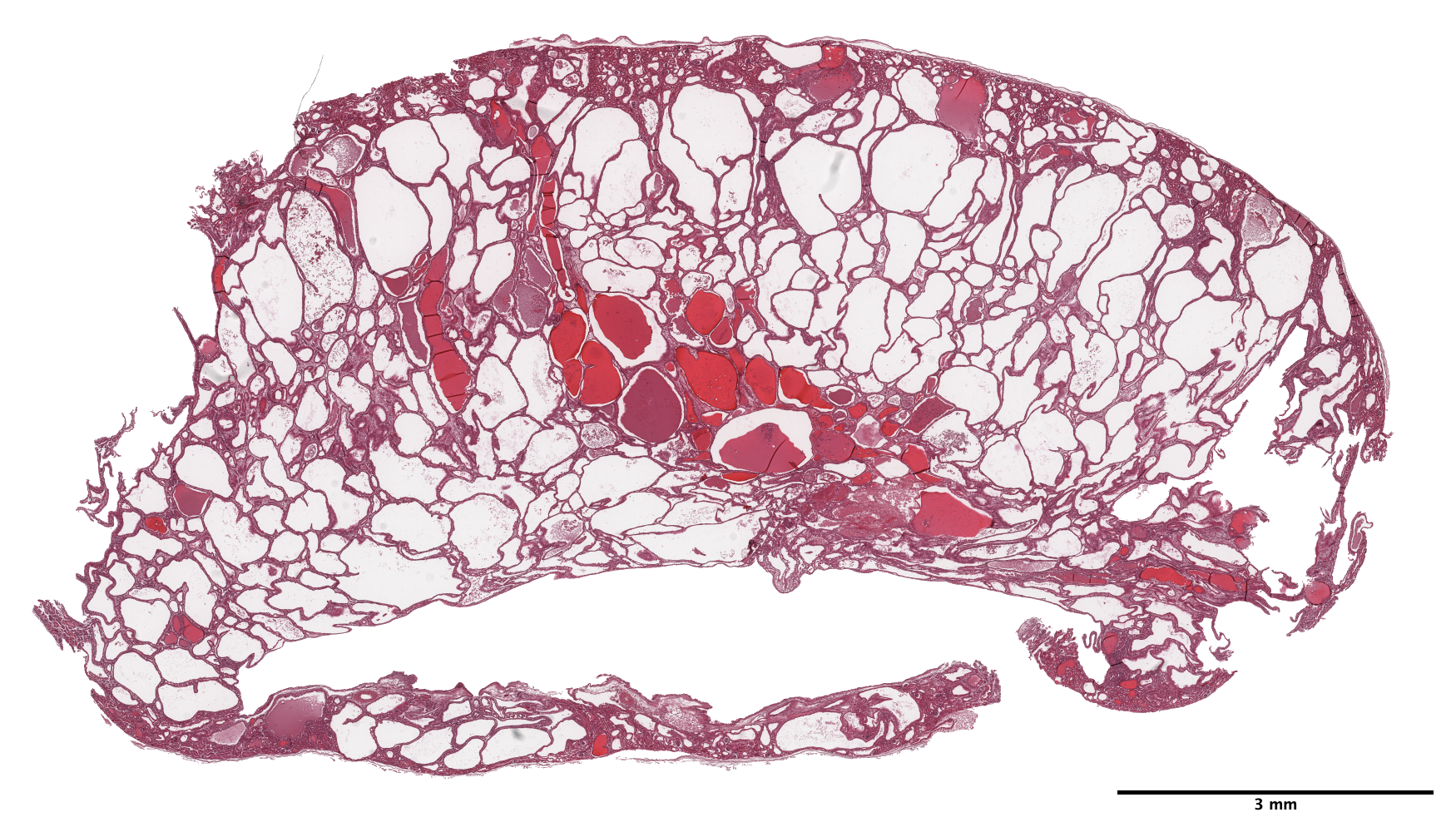

Supplement: Supplementary file 15 — Source Data for Figure 1 [file EMMM-15-e16877-s013.zip › Source_Data_Figure1/1A/Flcn:Tfe3 DKO.tif]

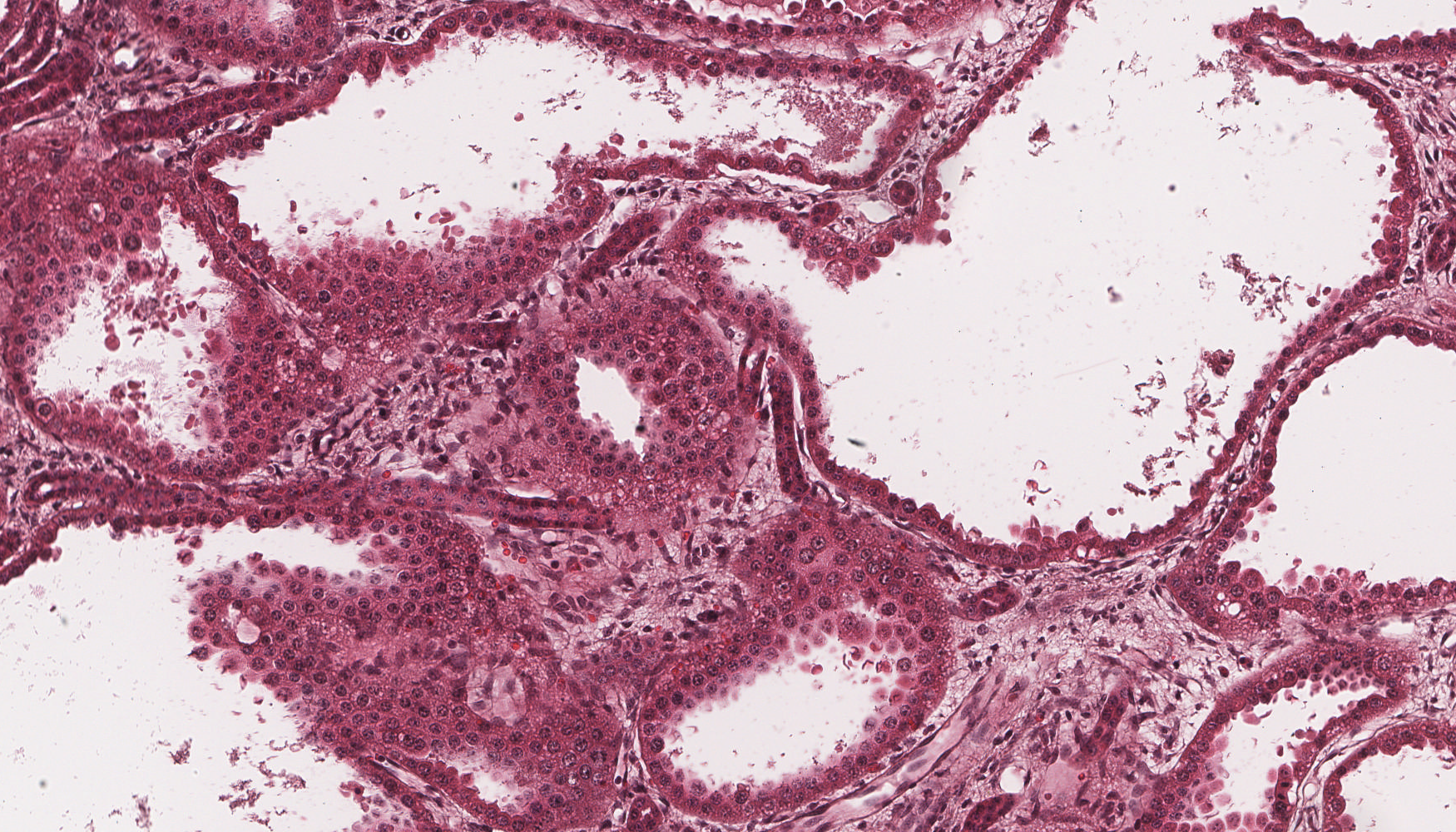

Supplement: Supplementary file 15 — Source Data for Figure 1 [file EMMM-15-e16877-s013.zip › Source_Data_Figure1/1A/Flcn:Tfe3 DKO-enlargement.tif]

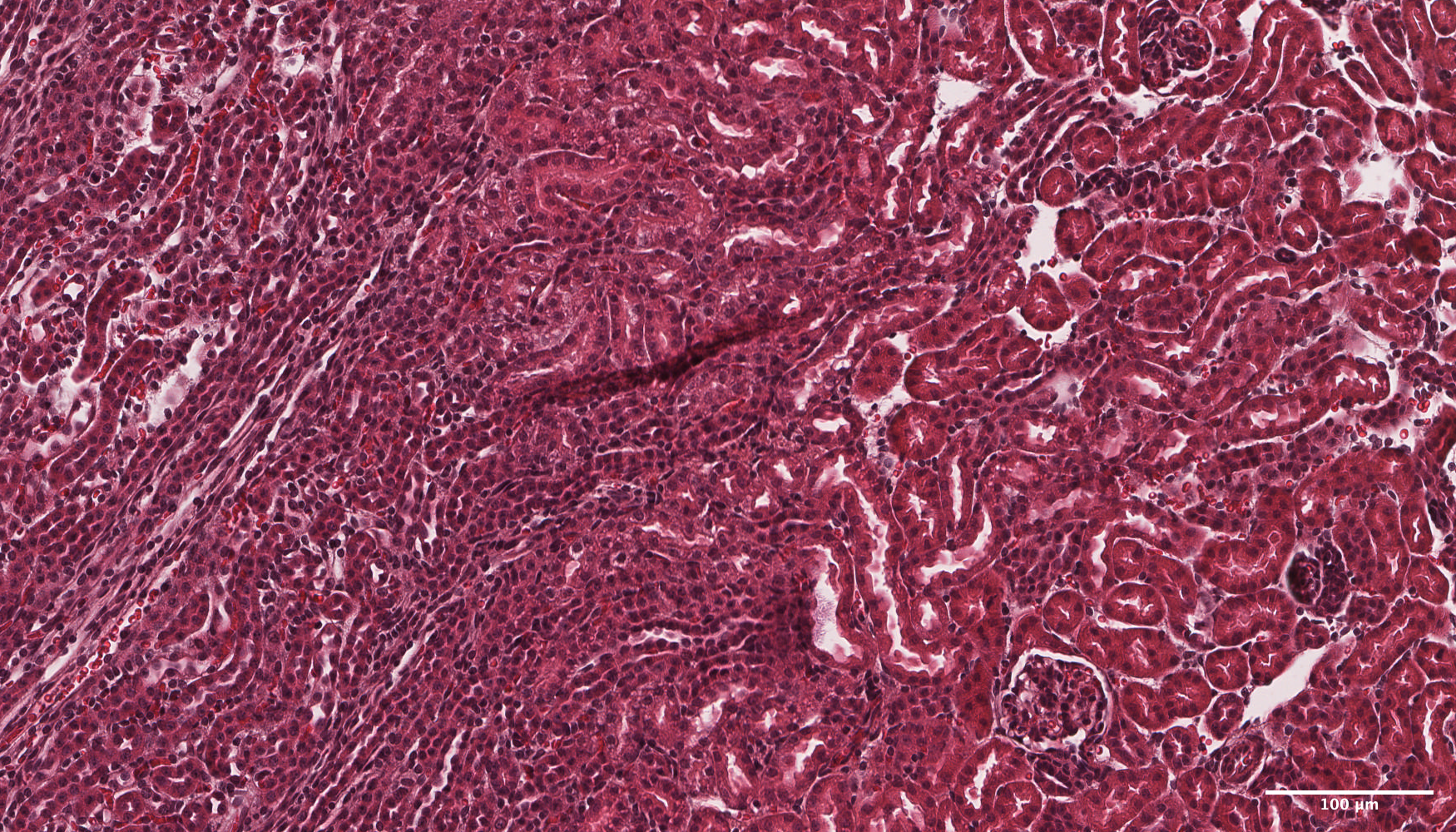

Supplement: Supplementary file 15 — Source Data for Figure 1 [file EMMM-15-e16877-s013.zip › Source_Data_Figure1/1A/Ctrl-enlargement.tif]

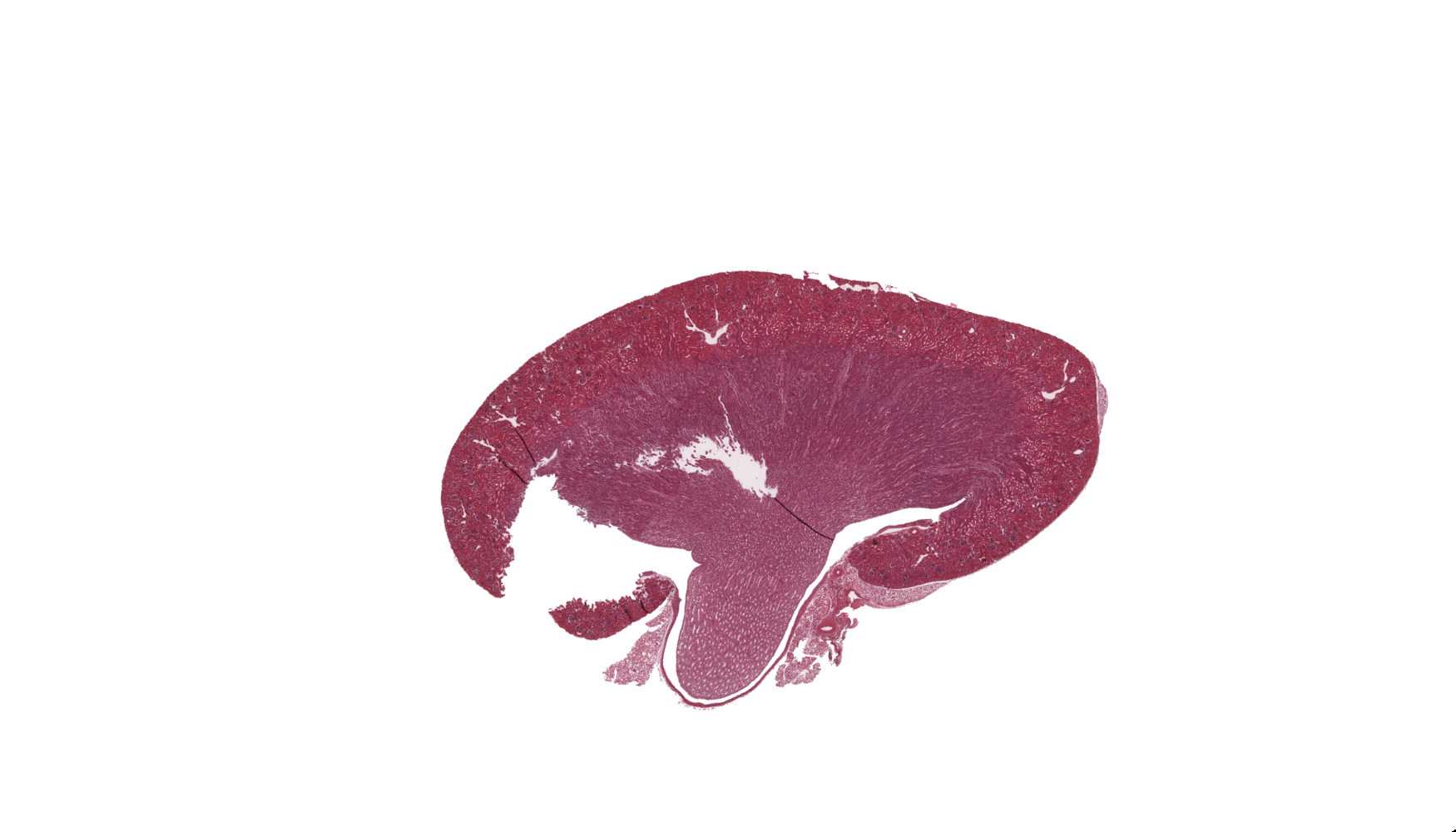

Supplement: Supplementary file 15 — Source Data for Figure 1 [file EMMM-15-e16877-s013.zip › Source_Data_Figure1/1A/Flcn:Tfeb DKO.tif]

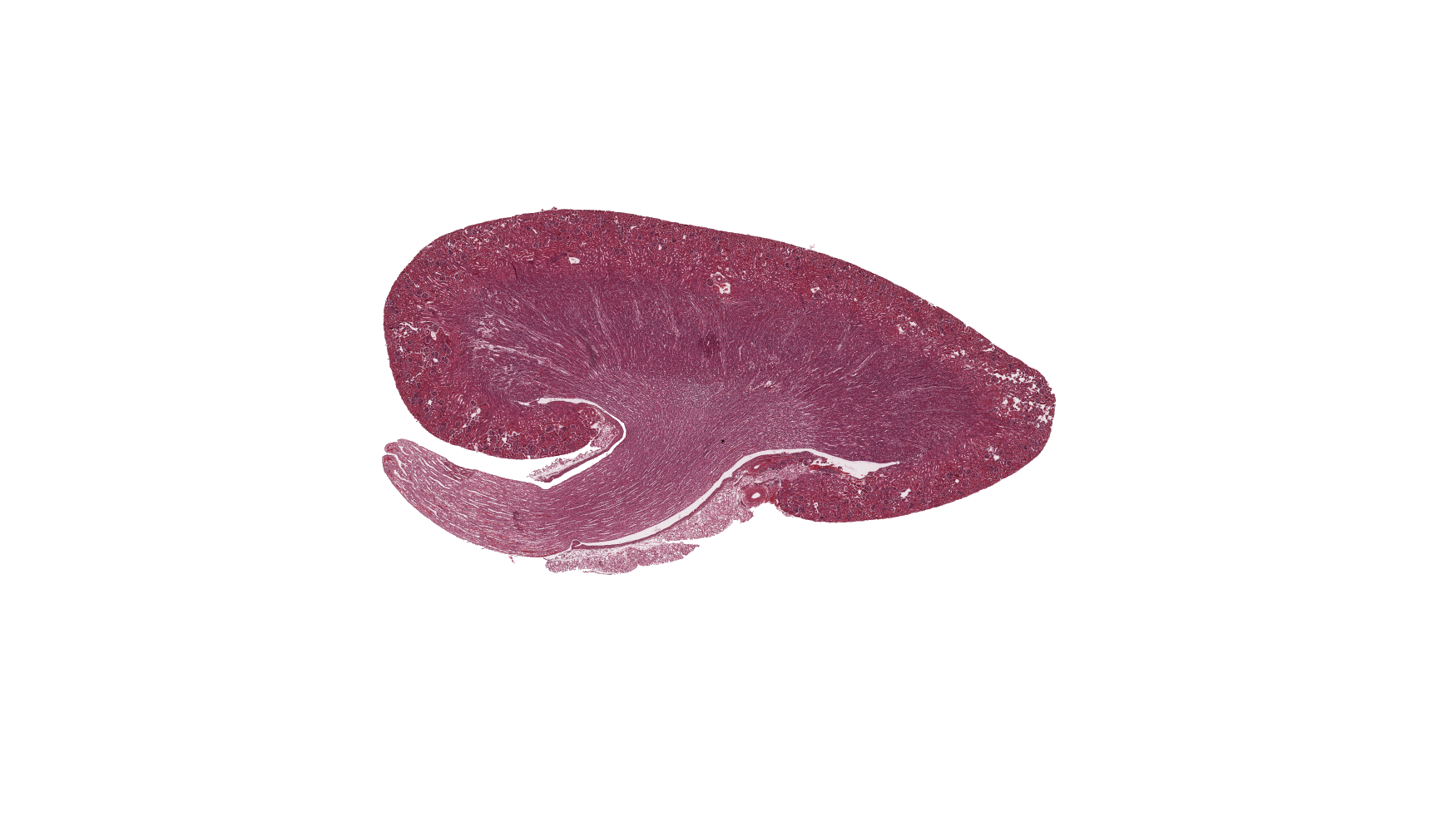

Supplement: Supplementary file 15 — Source Data for Figure 1 [file EMMM-15-e16877-s013.zip › Source_Data_Figure1/1A/Ctrl.tif]

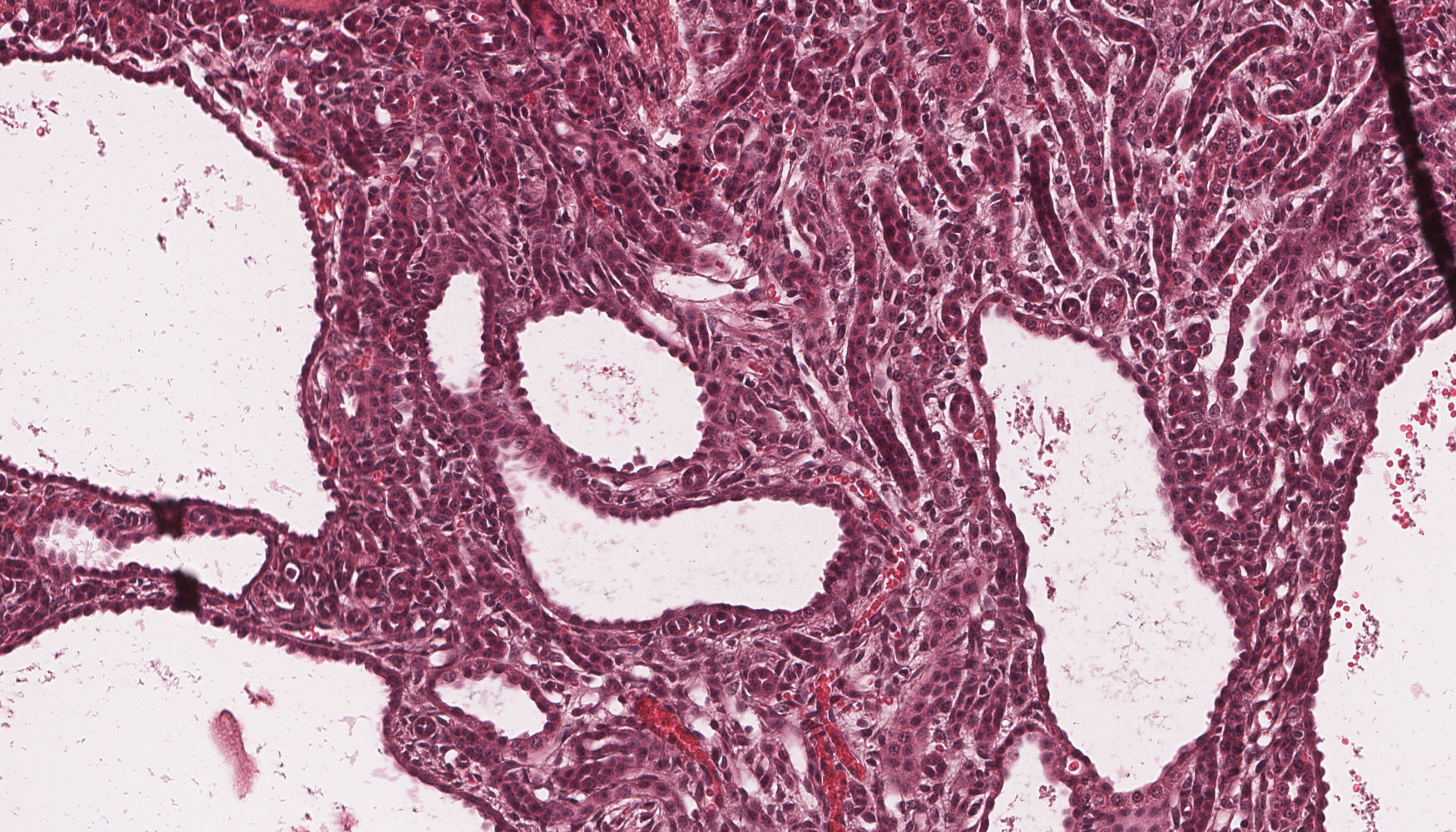

Supplement: Supplementary file 15 — Source Data for Figure 1 [file EMMM-15-e16877-s013.zip › Source_Data_Figure1/1A/Flcn:Tfe3 DKO; Tfeb-HET-enlargement.tif]

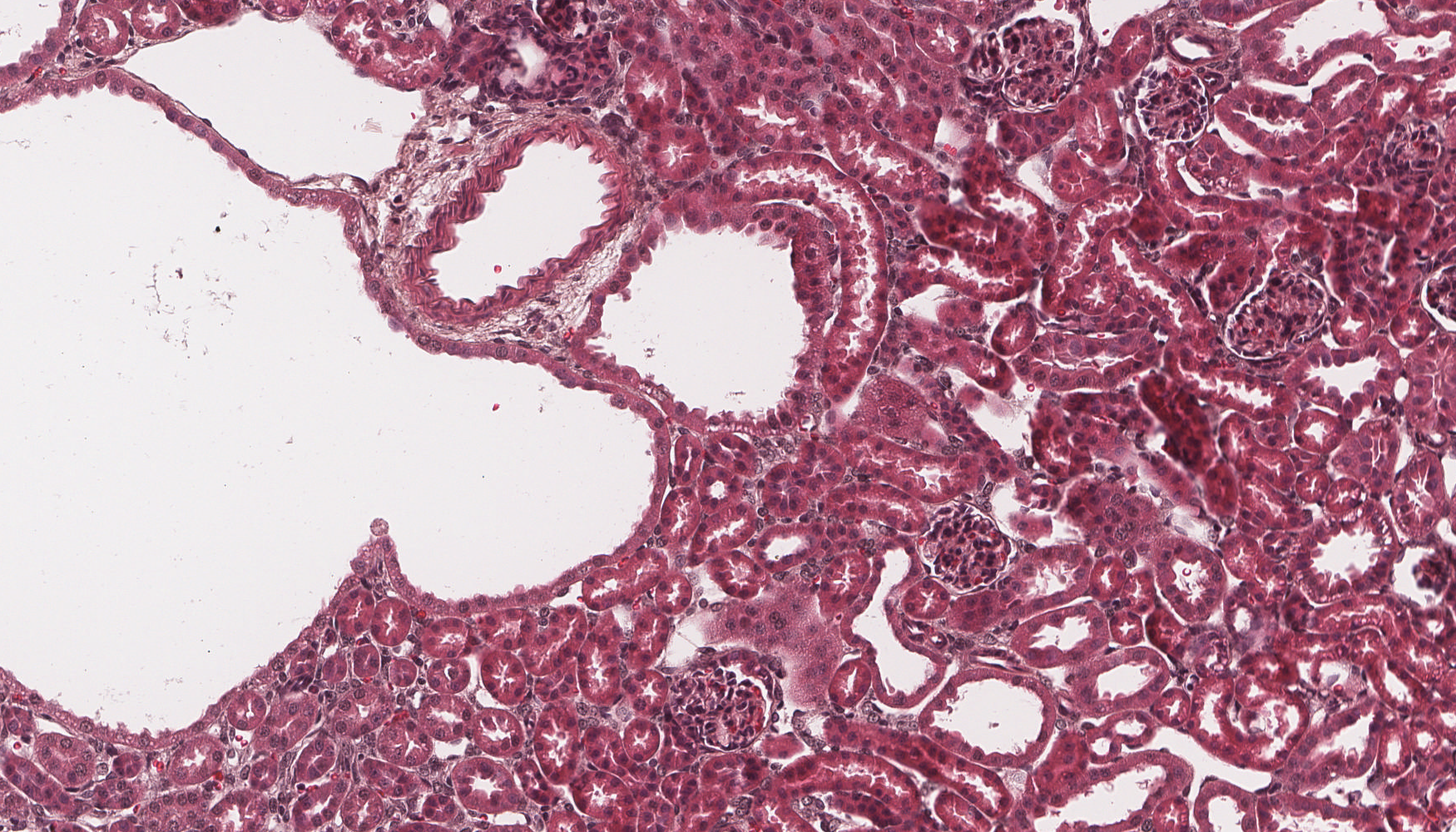

Supplement: Supplementary file 15 — Source Data for Figure 1 [file EMMM-15-e16877-s013.zip › Source_Data_Figure1/1A/Flcn KO; Tfeb-HET-enlargement.tif]

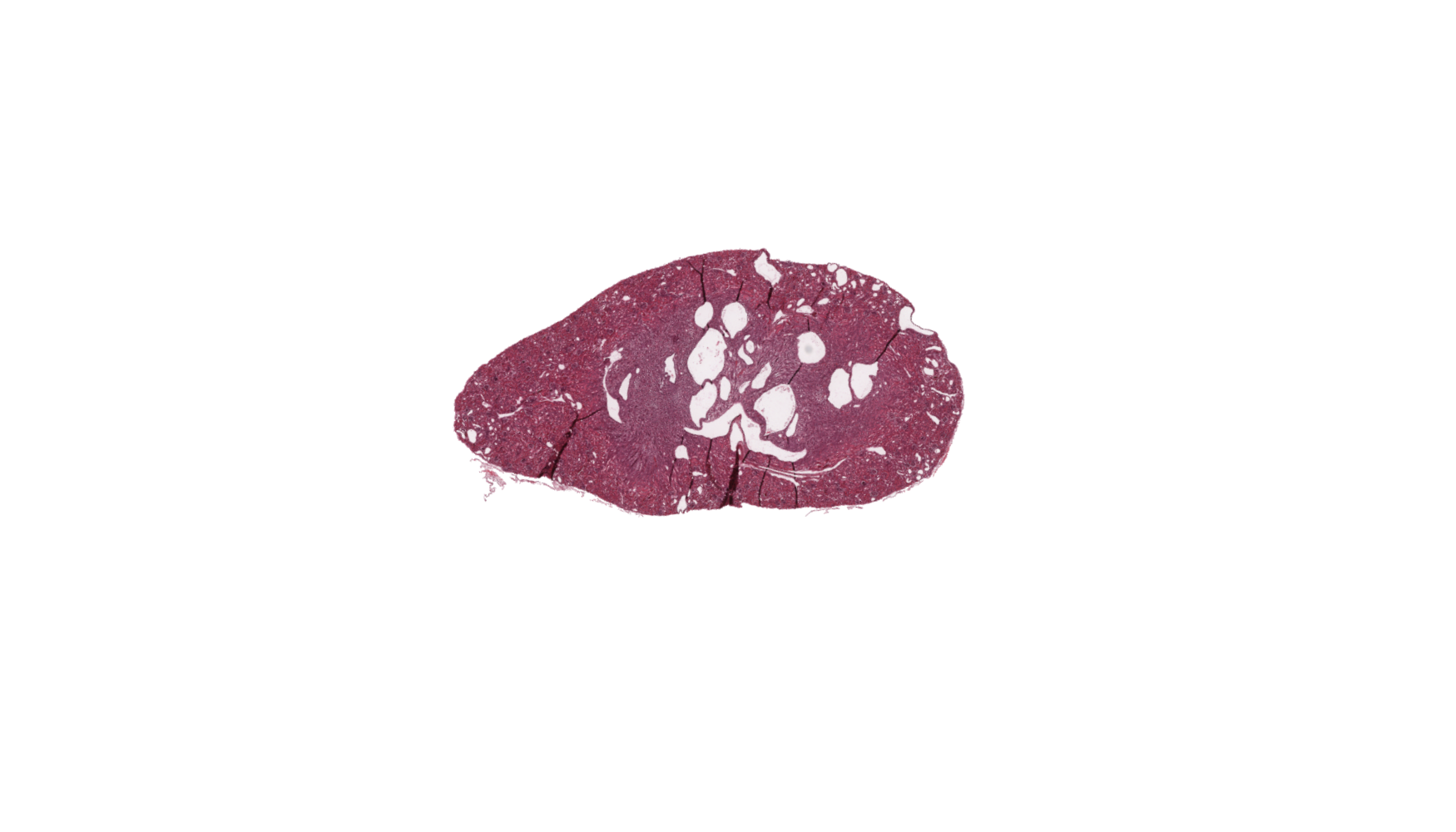

Supplement: Supplementary file 15 — Source Data for Figure 1 [file EMMM-15-e16877-s013.zip › Source_Data_Figure1/1A/Flcn:Tfe3 DKO; Tfeb-HET.tif]

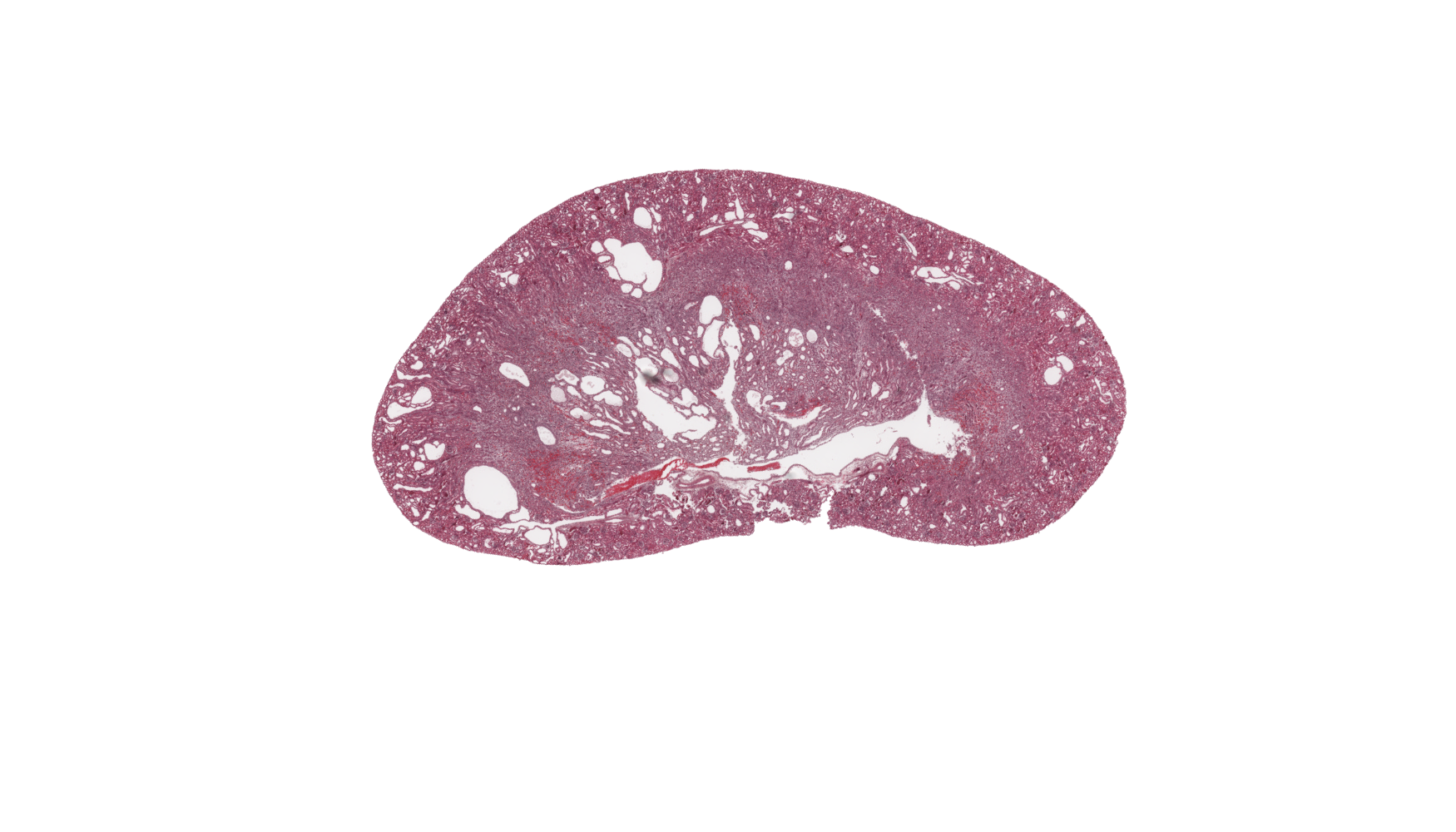

Supplement: Supplementary file 15 — Source Data for Figure 1 [file EMMM-15-e16877-s013.zip › Source_Data_Figure1/1A/Flcn KO; Tfeb-HET.tif]

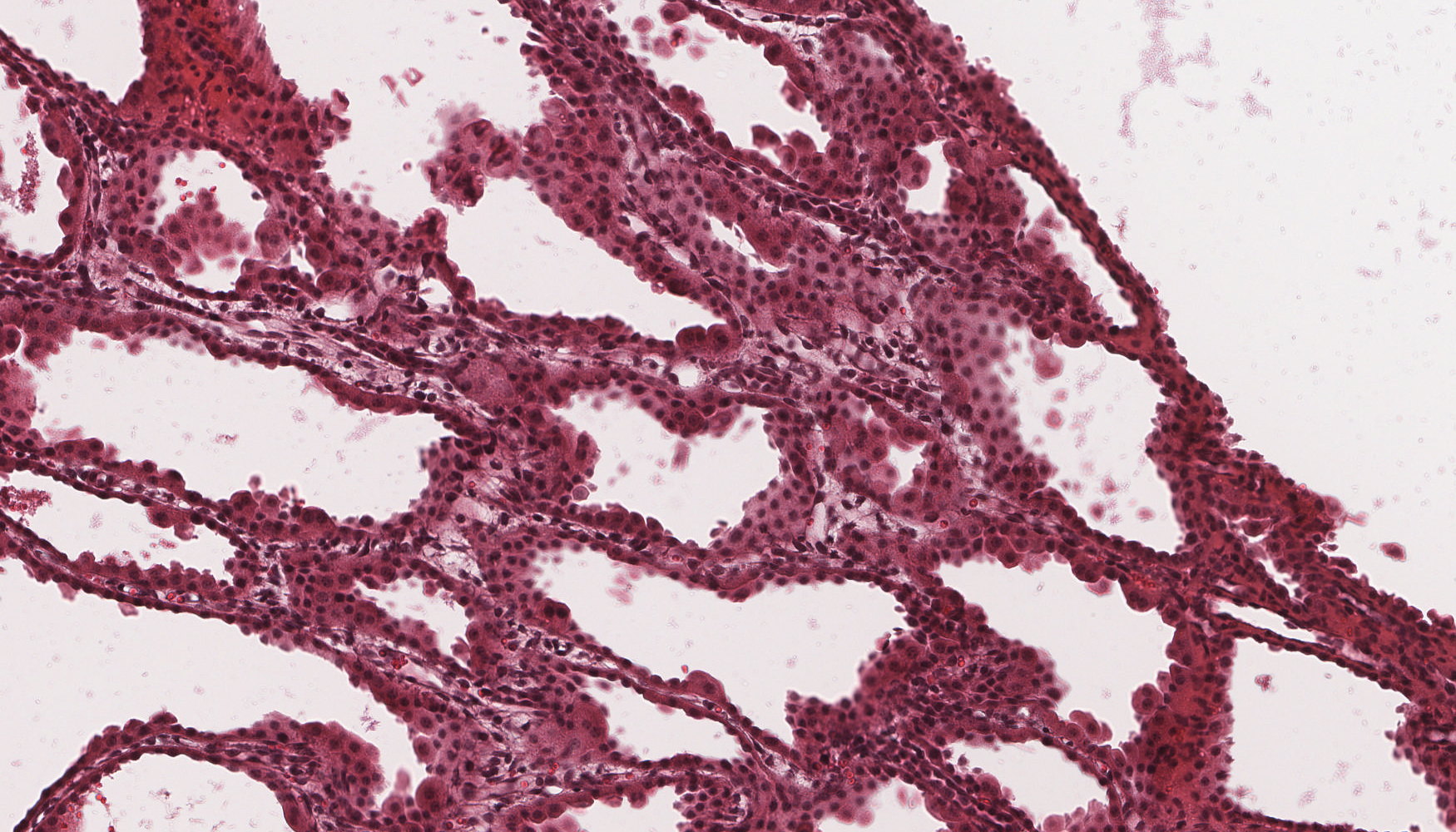

Supplement: Supplementary file 15 — Source Data for Figure 1 [file EMMM-15-e16877-s013.zip › Source_Data_Figure1/1A/Flcn KO-enlargement.tif]

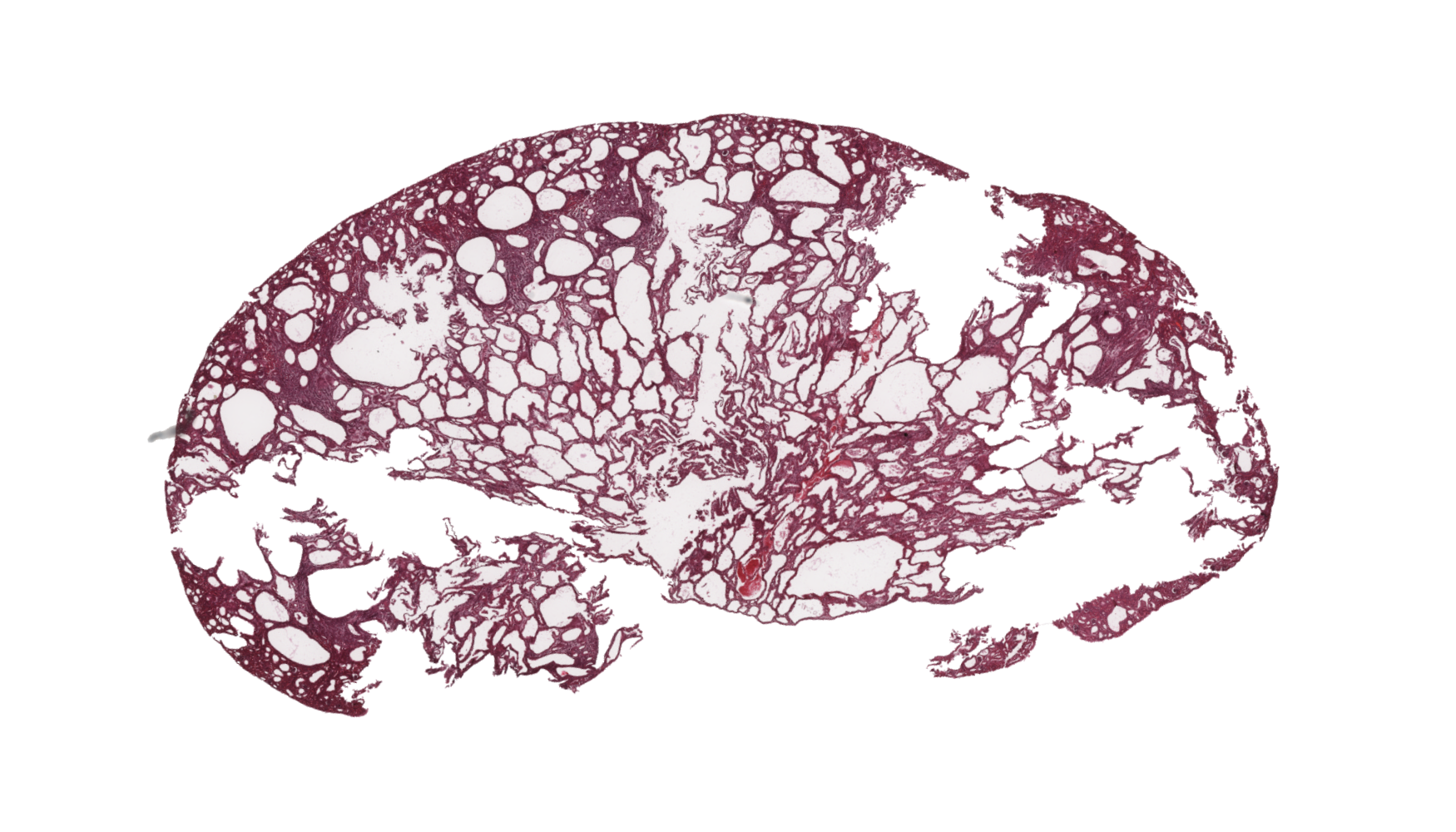

Supplement: Supplementary file 15 — Source Data for Figure 1 [file EMMM-15-e16877-s013.zip › Source_Data_Figure1/1A/Flcn KO.tif]

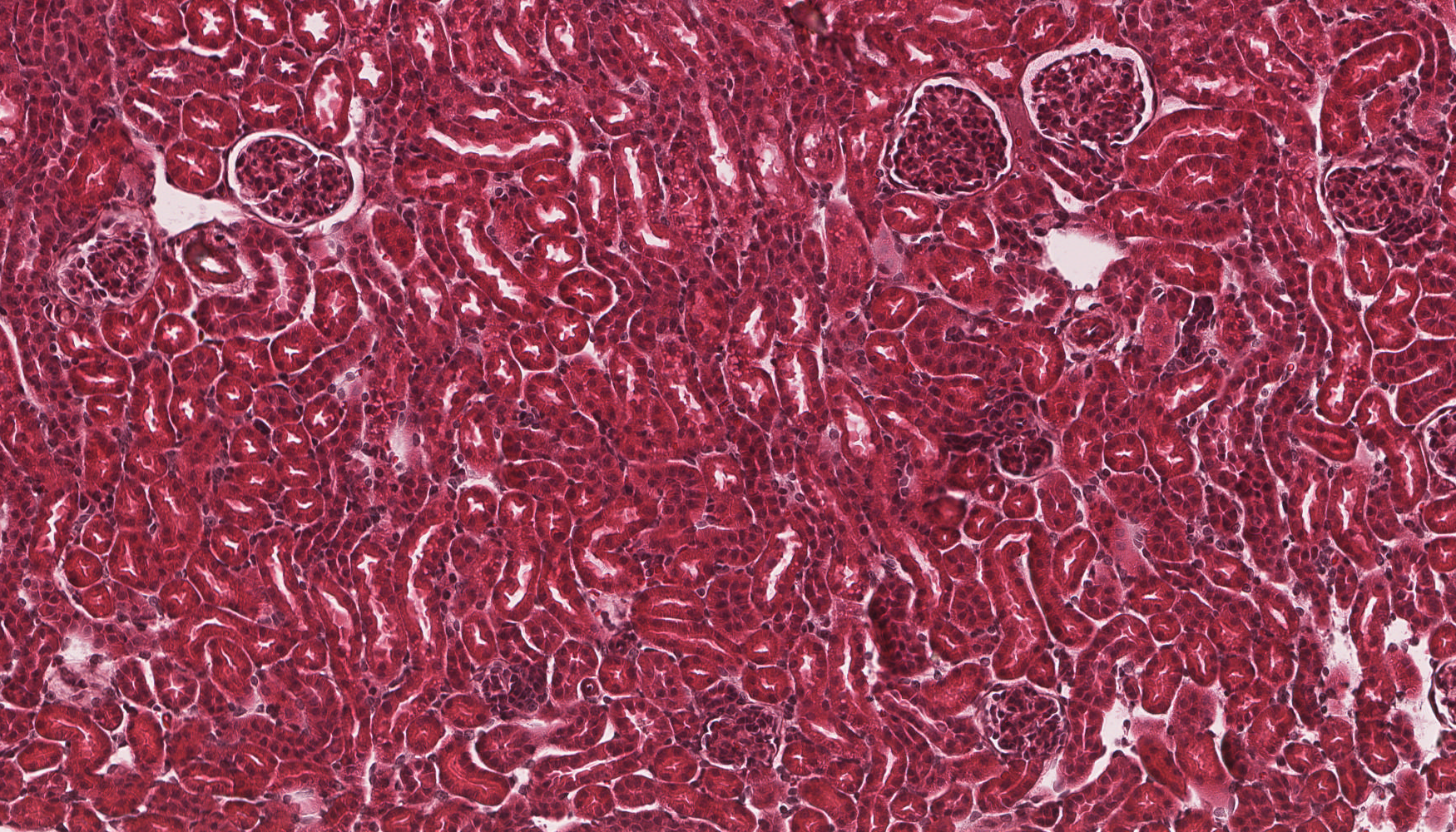

Supplement: Supplementary file 15 — Source Data for Figure 1 [file EMMM-15-e16877-s013.zip › Source_Data_Figure1/1A/Flcn:Tfeb DKO-enlargement.tif]

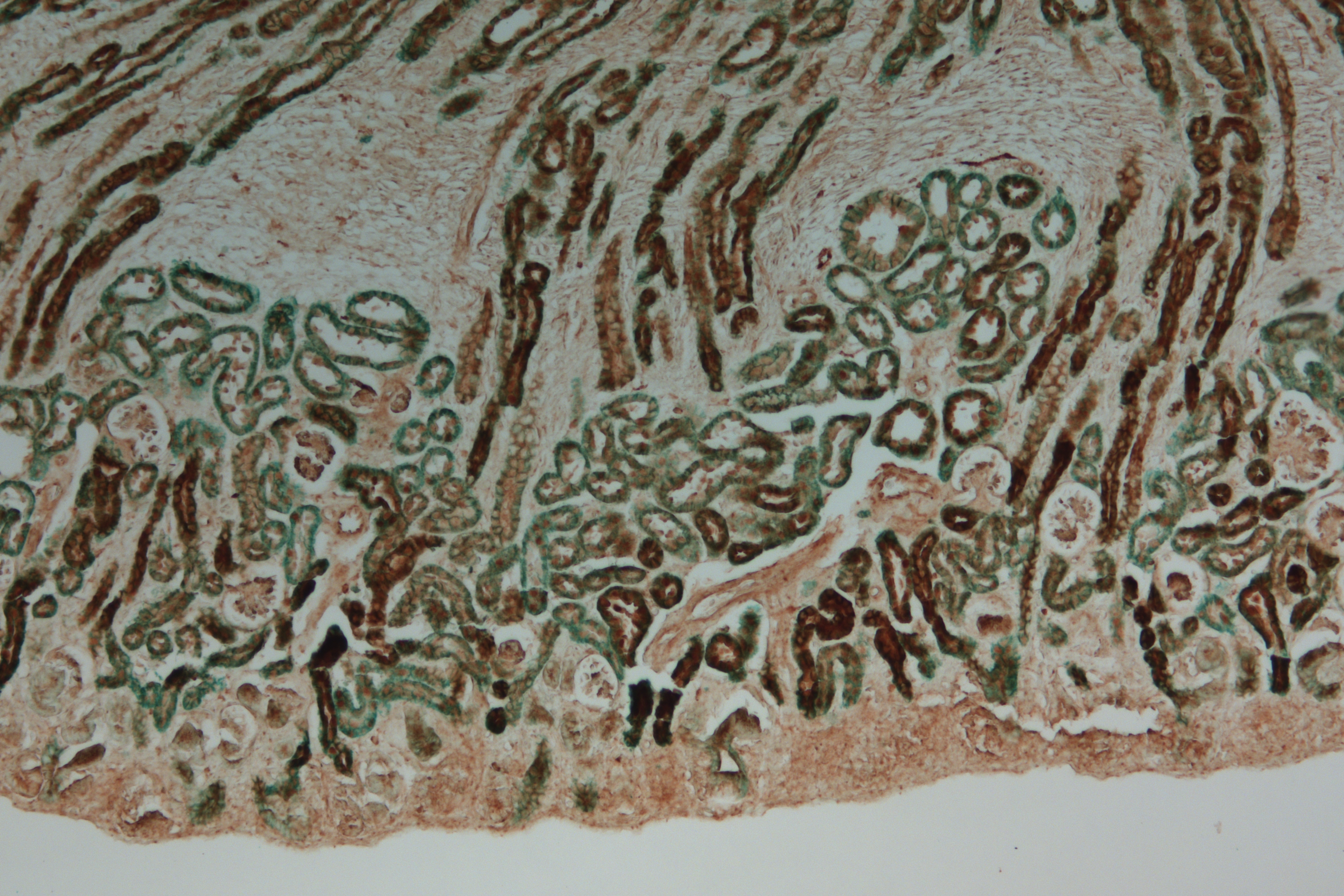

Supplement: Supplementary file 15 — Source Data for Figure 1 [file EMMM-15-e16877-s013.zip › Source_Data_Figure1/1D/8 ko FLCN tfeb CDH16 10x.tif]

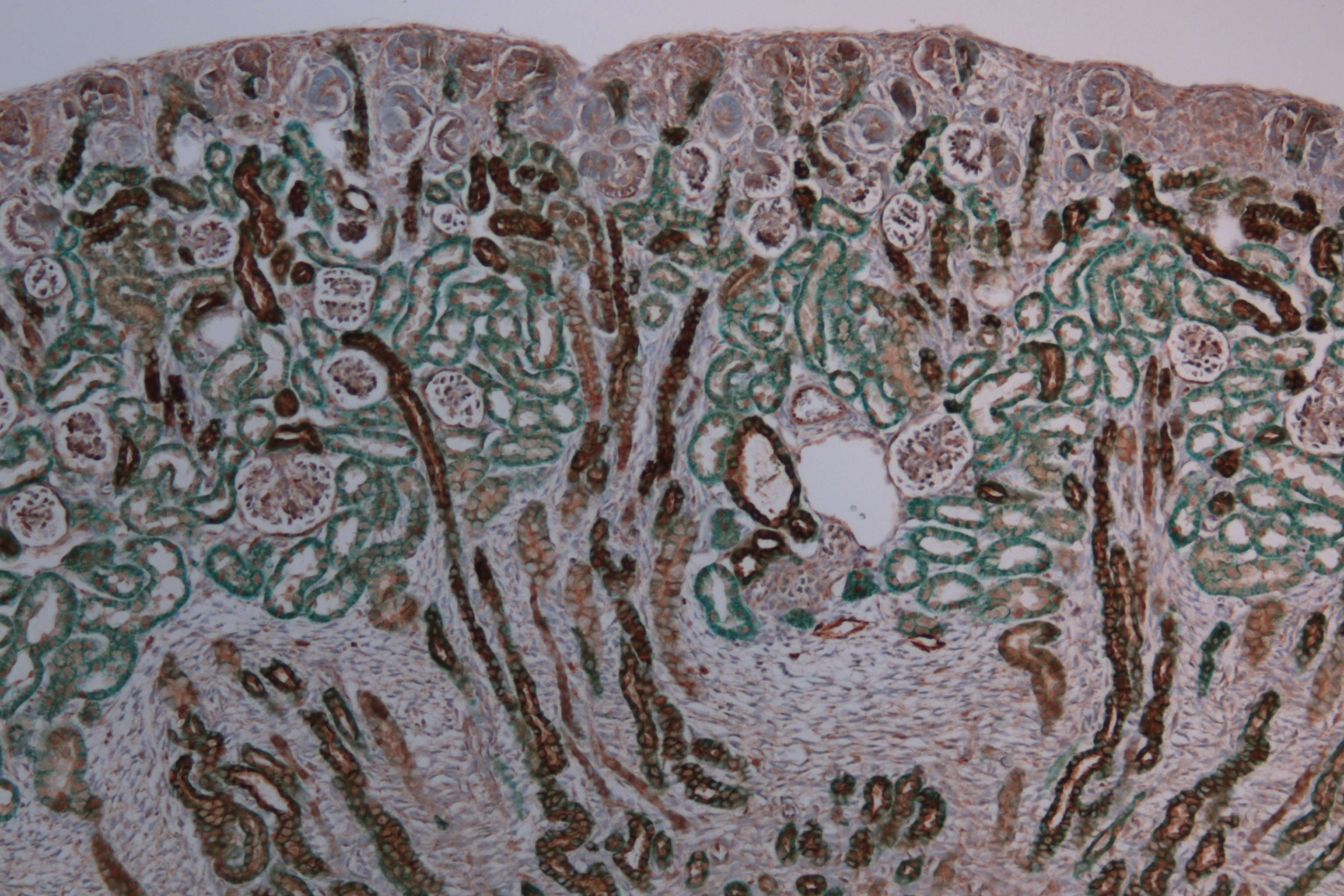

Supplement: Supplementary file 15 — Source Data for Figure 1 [file EMMM-15-e16877-s013.zip › Source_Data_Figure1/1D/6 ctrl tfeb cad 10x_ch00.tif]

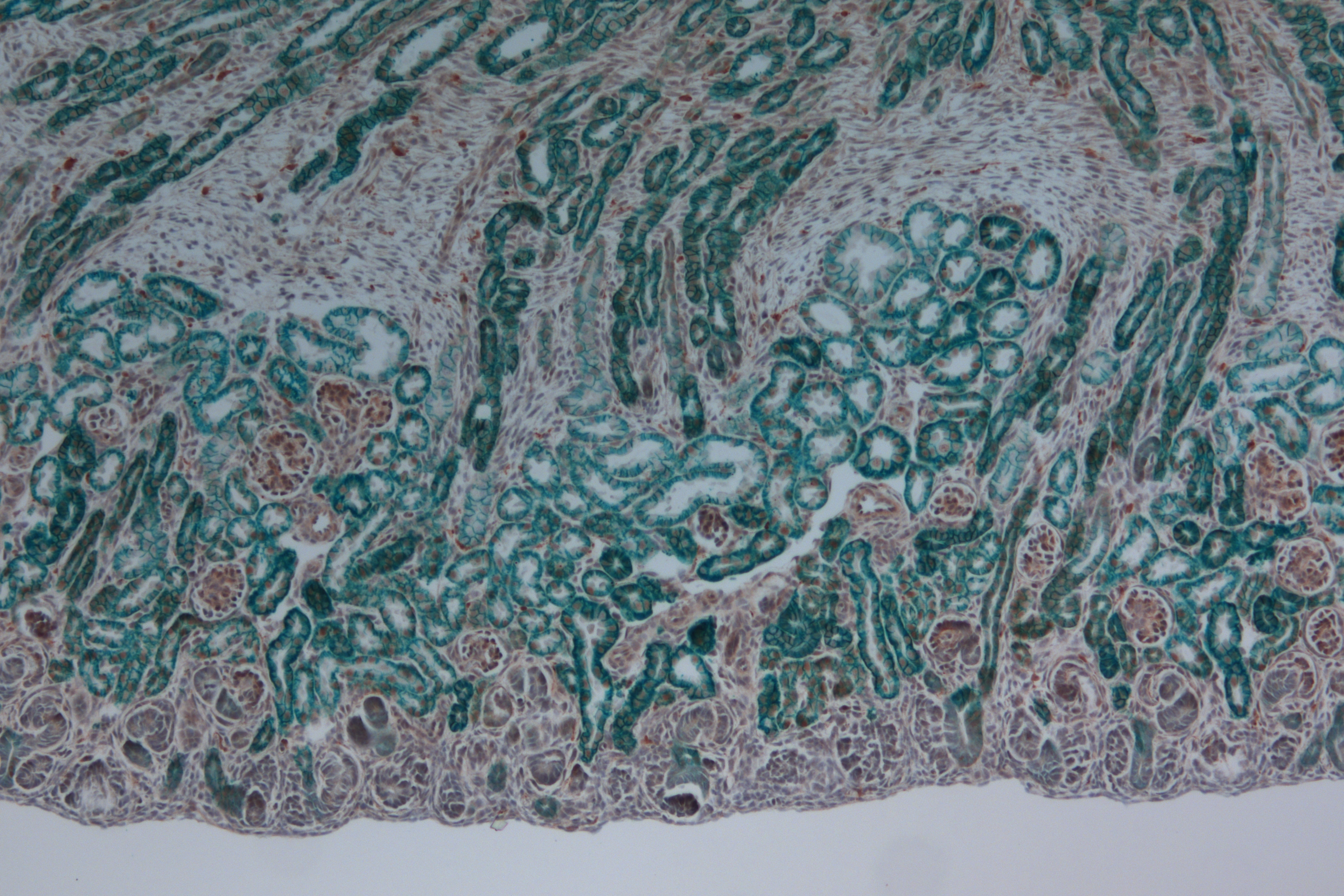

Supplement: Supplementary file 15 — Source Data for Figure 1 [file EMMM-15-e16877-s013.zip › Source_Data_Figure1/1D/8 ko FLCN tfe3 CDH16 10x.tif]

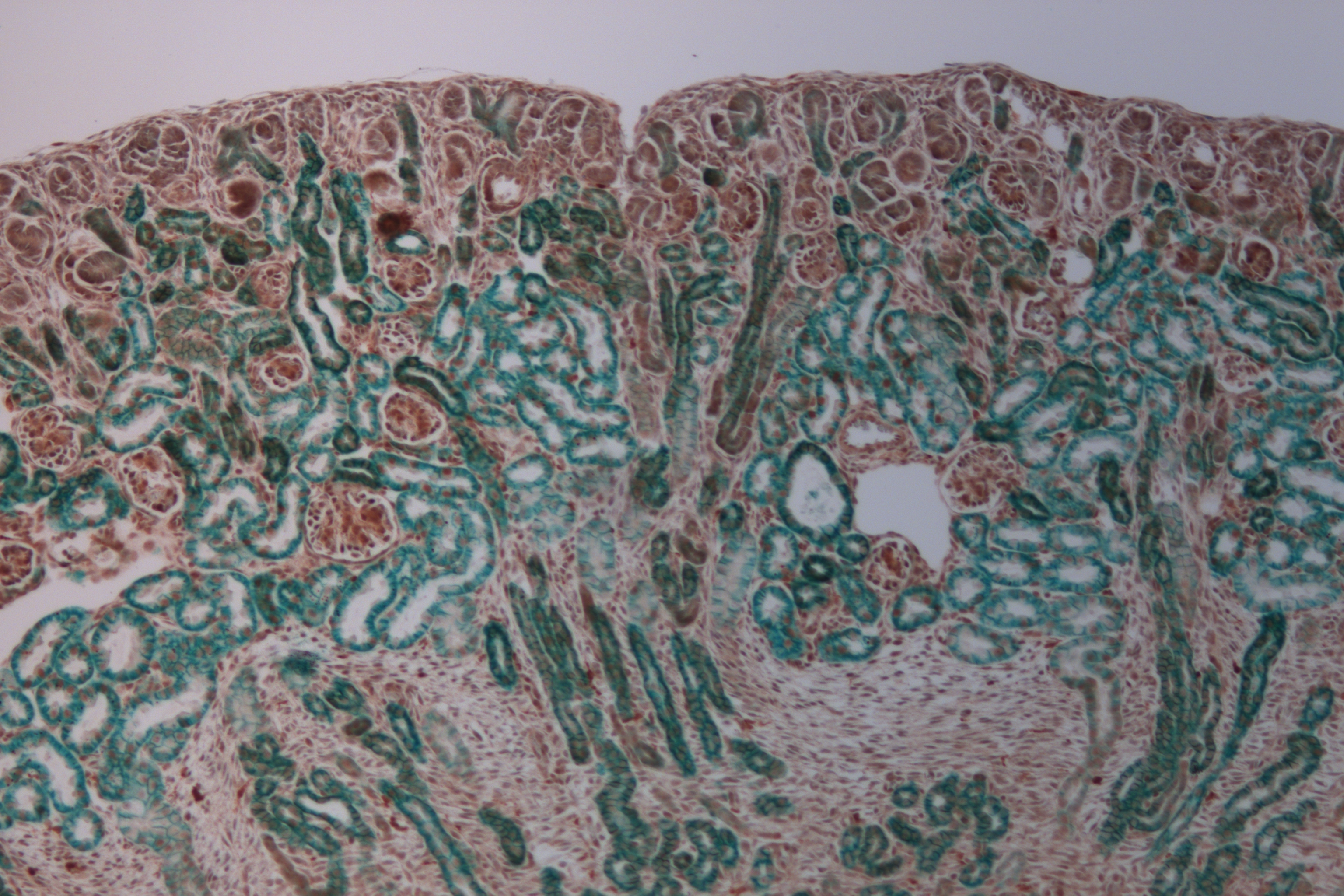

Supplement: Supplementary file 15 — Source Data for Figure 1 [file EMMM-15-e16877-s013.zip › Source_Data_Figure1/1D/6 ctrtfe3 cad 10x_ch00.tif]

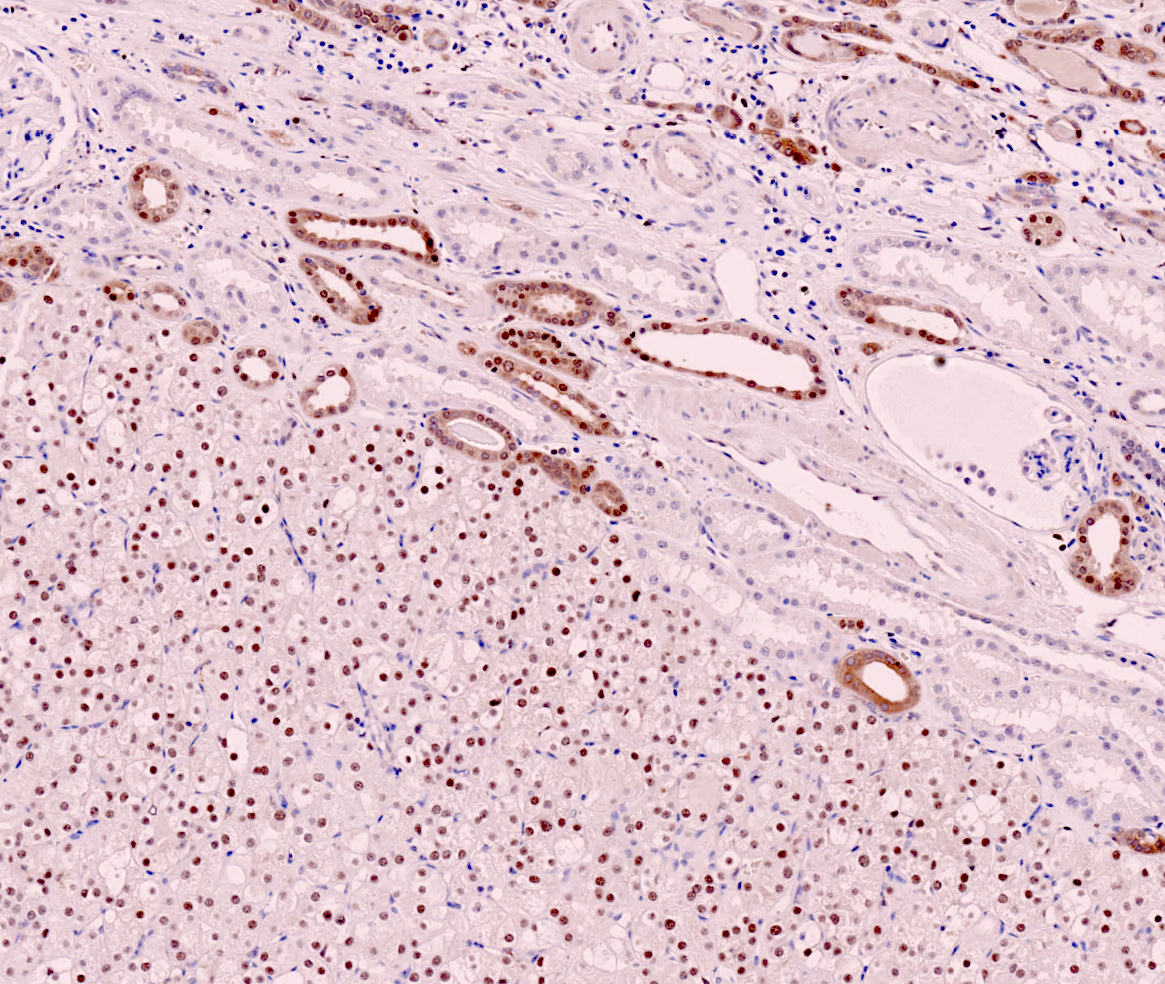

Supplement: Supplementary file 16 — Source Data for Figure 2 [file EMMM-15-e16877-s017.zip › Source_Data_Figure2/2A/TIF images for Figure 2A/BHD Patient 3 Tumor TFEB.tif]

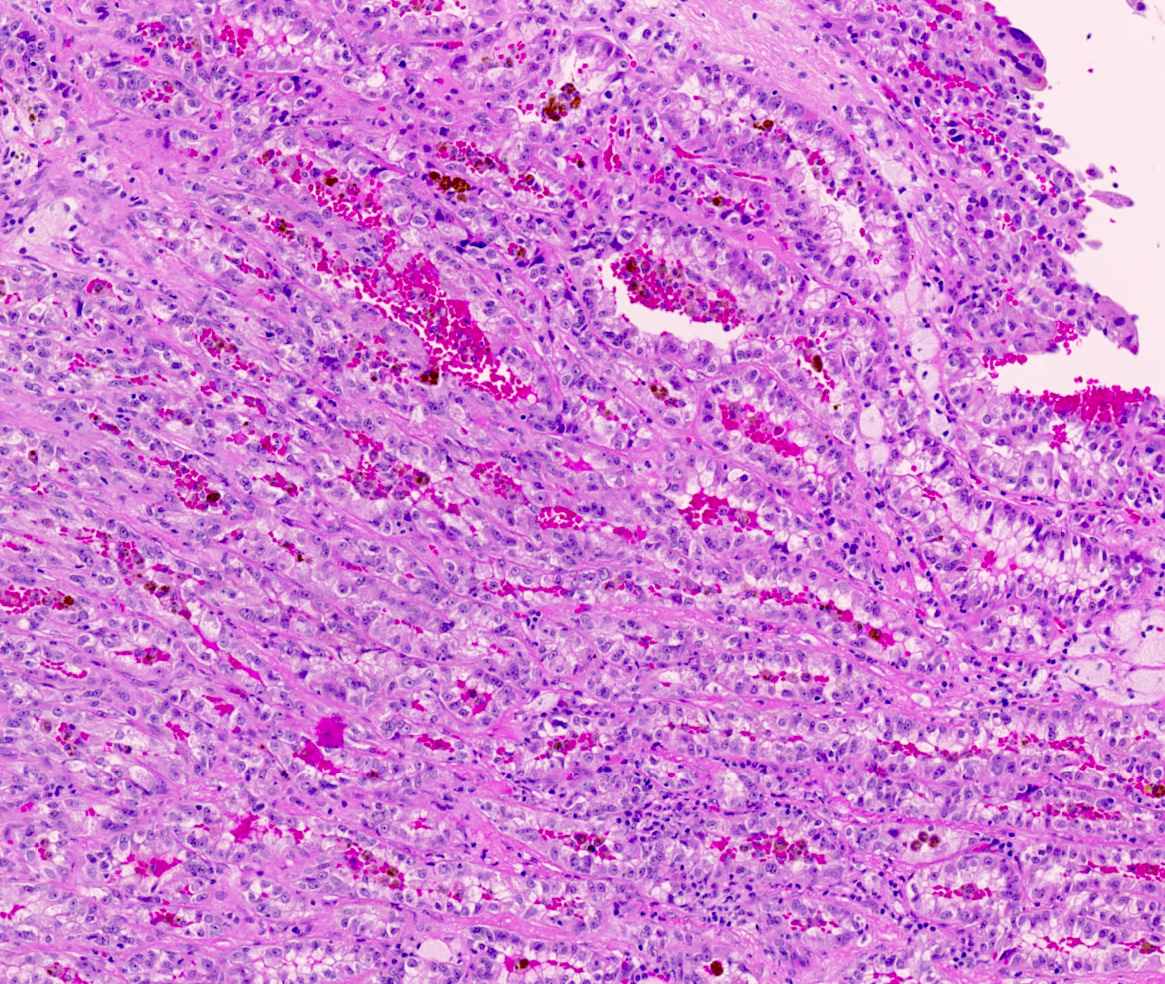

Supplement: Supplementary file 16 — Source Data for Figure 2 [file EMMM-15-e16877-s017.zip › Source_Data_Figure2/2A/TIF images for Figure 2A/BHD Patient 4 H&E.tif]

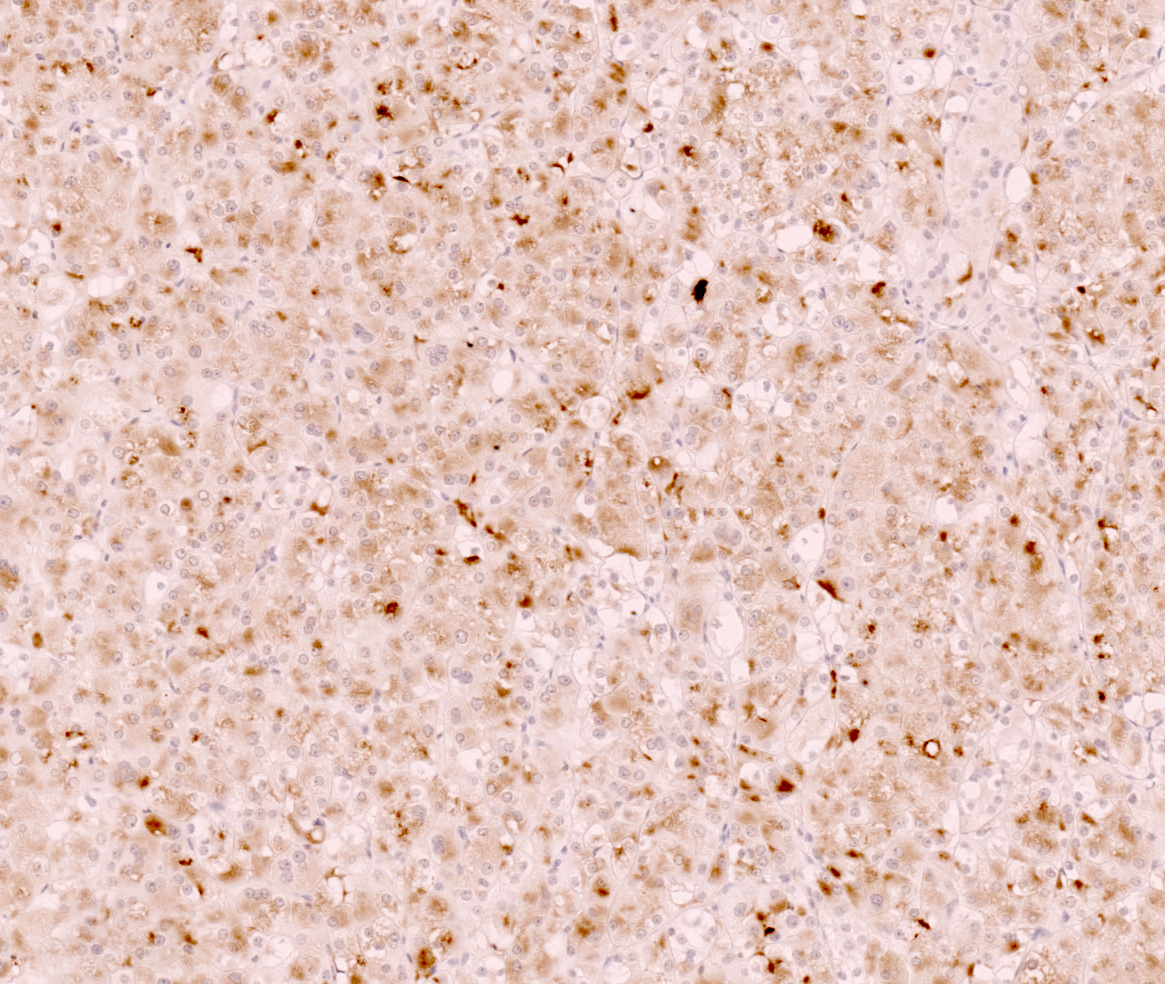

Supplement: Supplementary file 16 — Source Data for Figure 2 [file EMMM-15-e16877-s017.zip › Source_Data_Figure2/2A/TIF images for Figure 2A/BHD Patient 1 NPC1.tif]

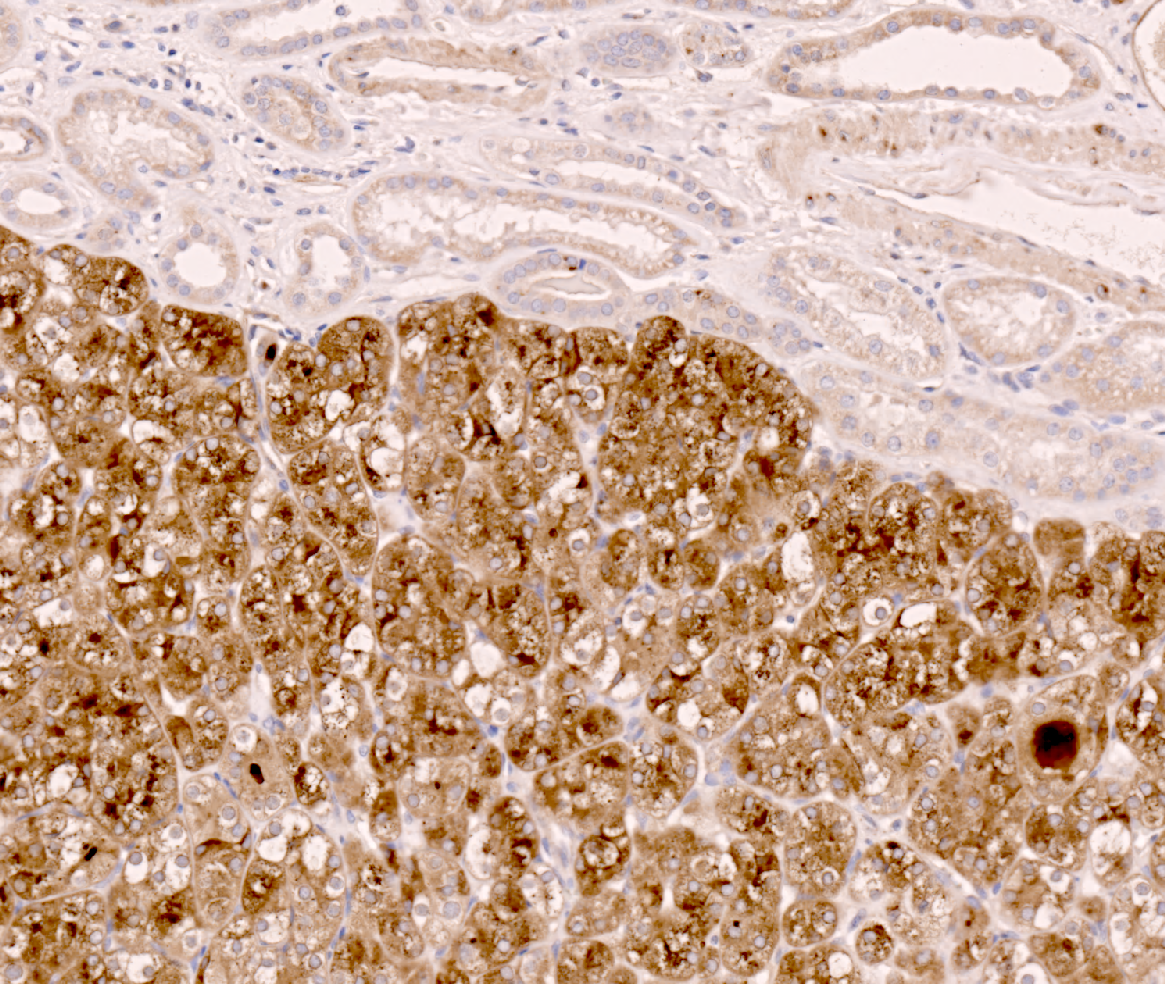

Supplement: Supplementary file 16 — Source Data for Figure 2 [file EMMM-15-e16877-s017.zip › Source_Data_Figure2/2A/TIF images for Figure 2A/BHD Patient 3 Tumor GPNMB.tif]

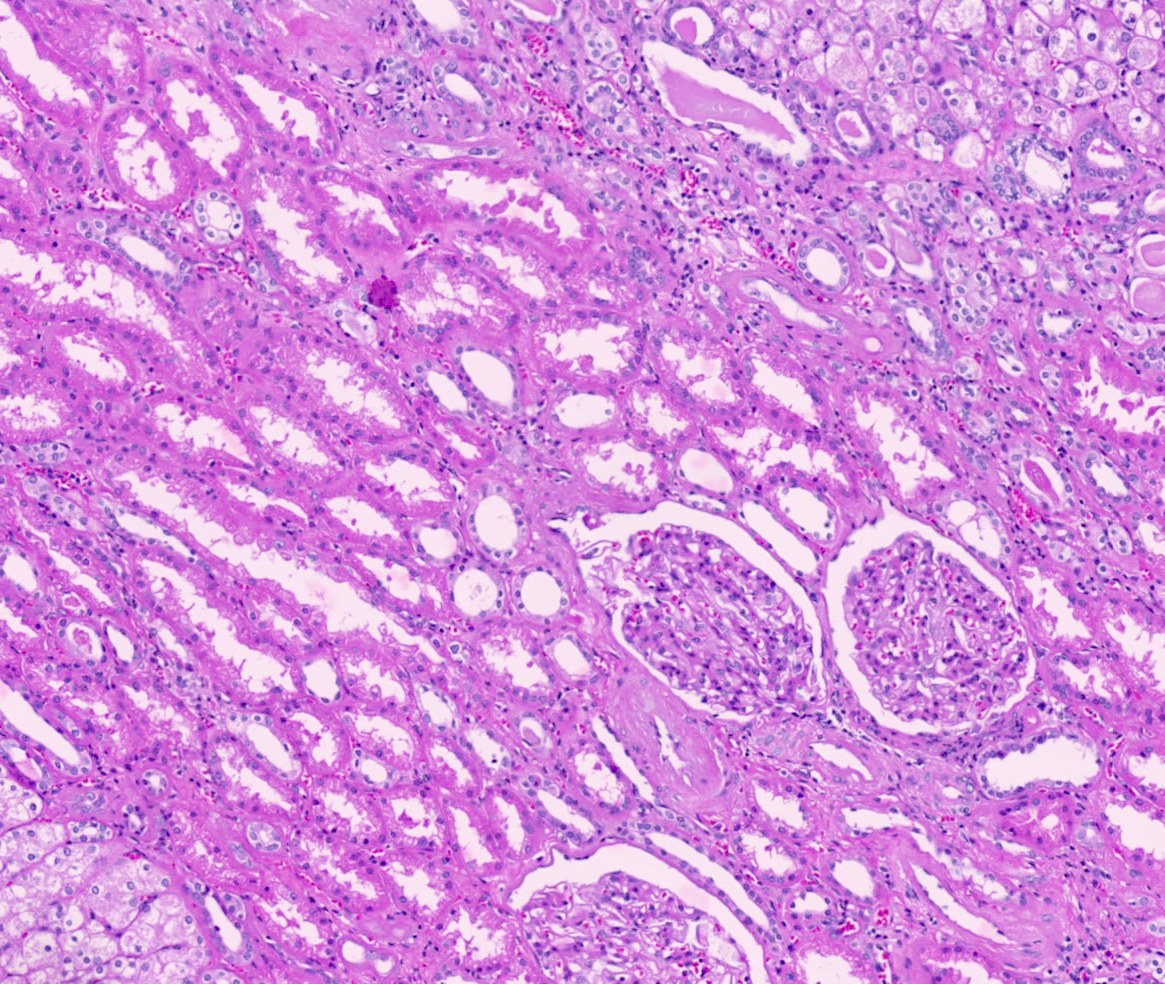

Supplement: Supplementary file 16 — Source Data for Figure 2 [file EMMM-15-e16877-s017.zip › Source_Data_Figure2/2A/TIF images for Figure 2A/BHD Patient 3 Normal H&E.tif]

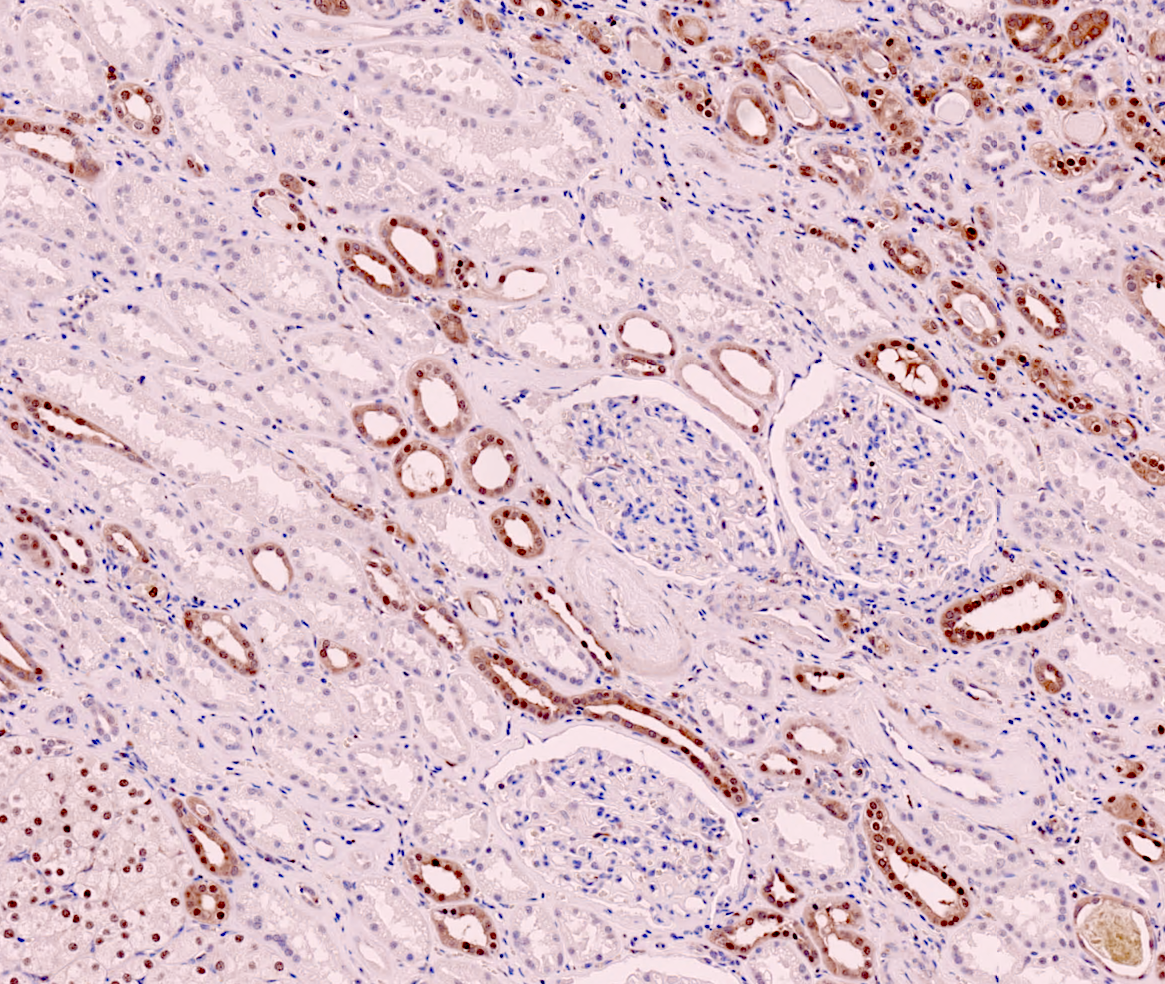

Supplement: Supplementary file 16 — Source Data for Figure 2 [file EMMM-15-e16877-s017.zip › Source_Data_Figure2/2A/TIF images for Figure 2A/BHD Patient 3 Normal TFEB.tif]

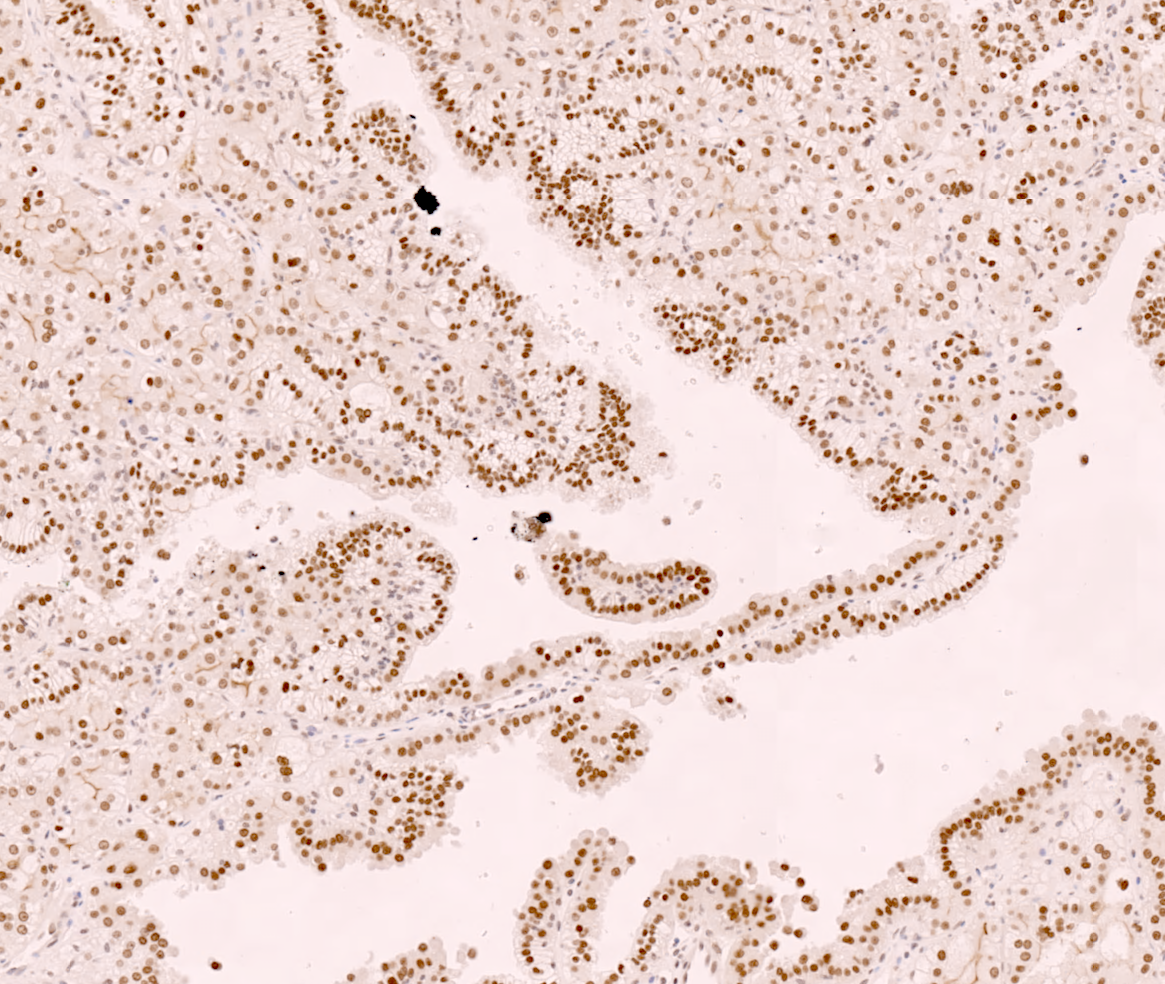

Supplement: Supplementary file 16 — Source Data for Figure 2 [file EMMM-15-e16877-s017.zip › Source_Data_Figure2/2A/TIF images for Figure 2A/BHD Patient 5 TFE3.tif]

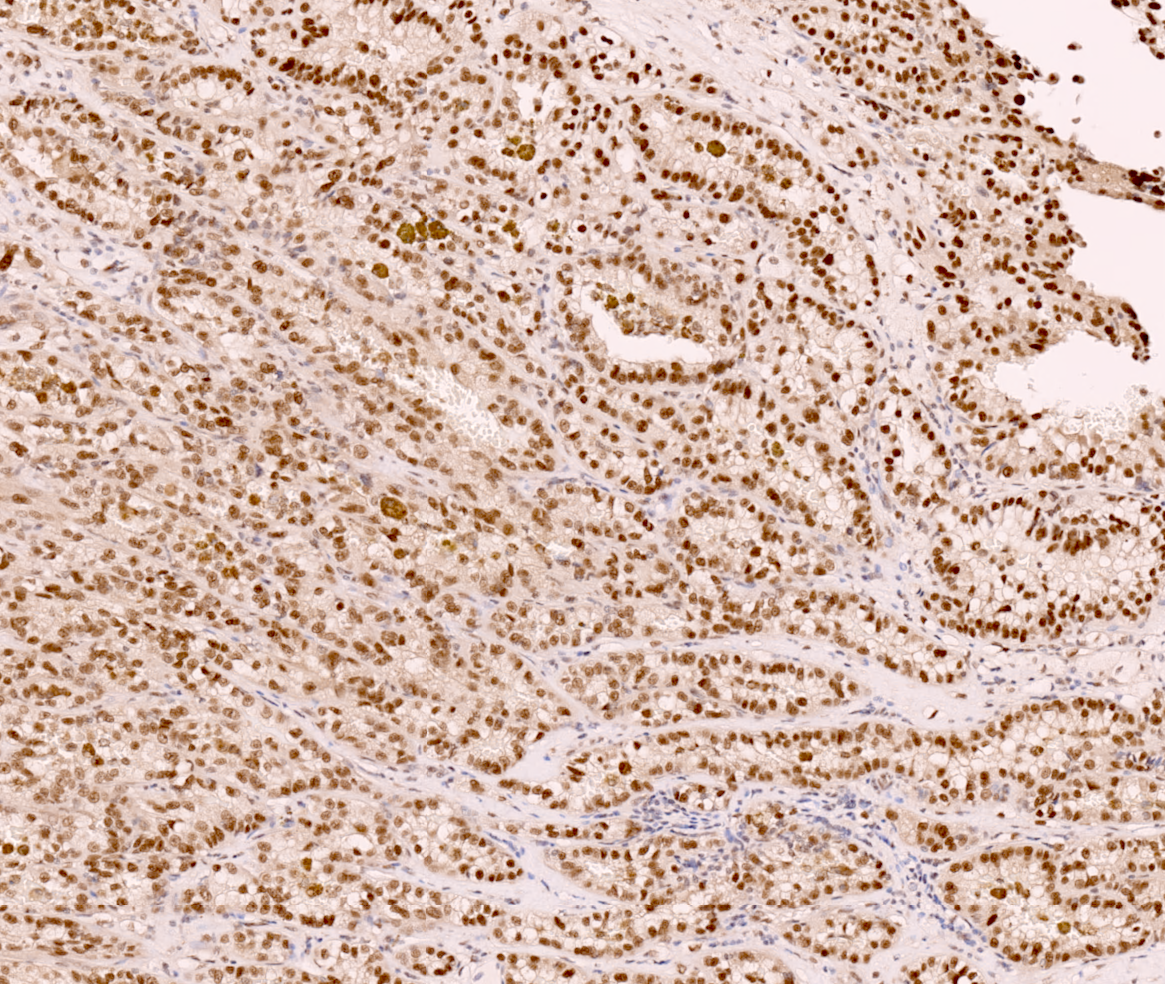

Supplement: Supplementary file 16 — Source Data for Figure 2 [file EMMM-15-e16877-s017.zip › Source_Data_Figure2/2A/TIF images for Figure 2A/BHD Patient 4 TFE3.tif]

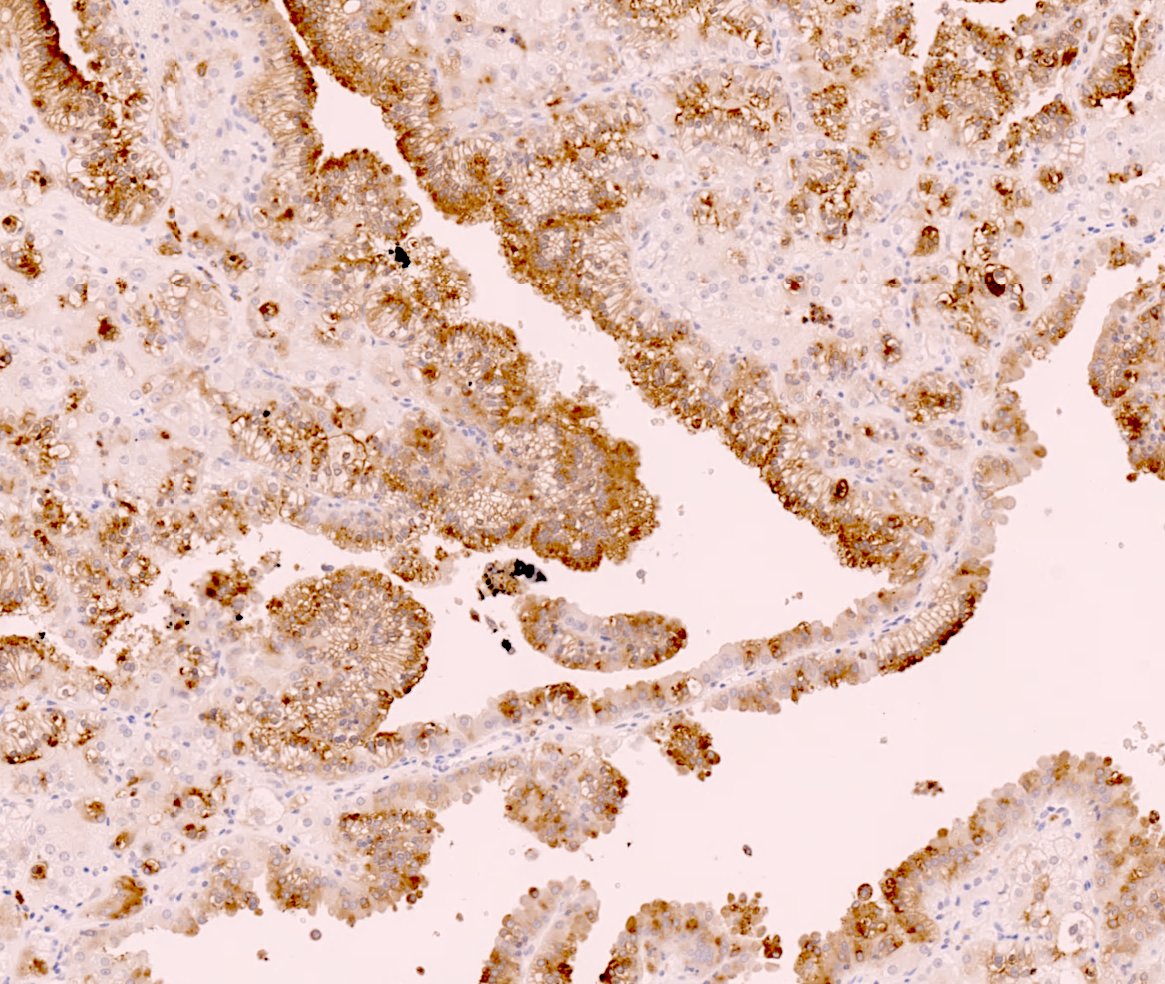

Supplement: Supplementary file 16 — Source Data for Figure 2 [file EMMM-15-e16877-s017.zip › Source_Data_Figure2/2A/TIF images for Figure 2A/BHD Patient 5 GPNMB.tif]

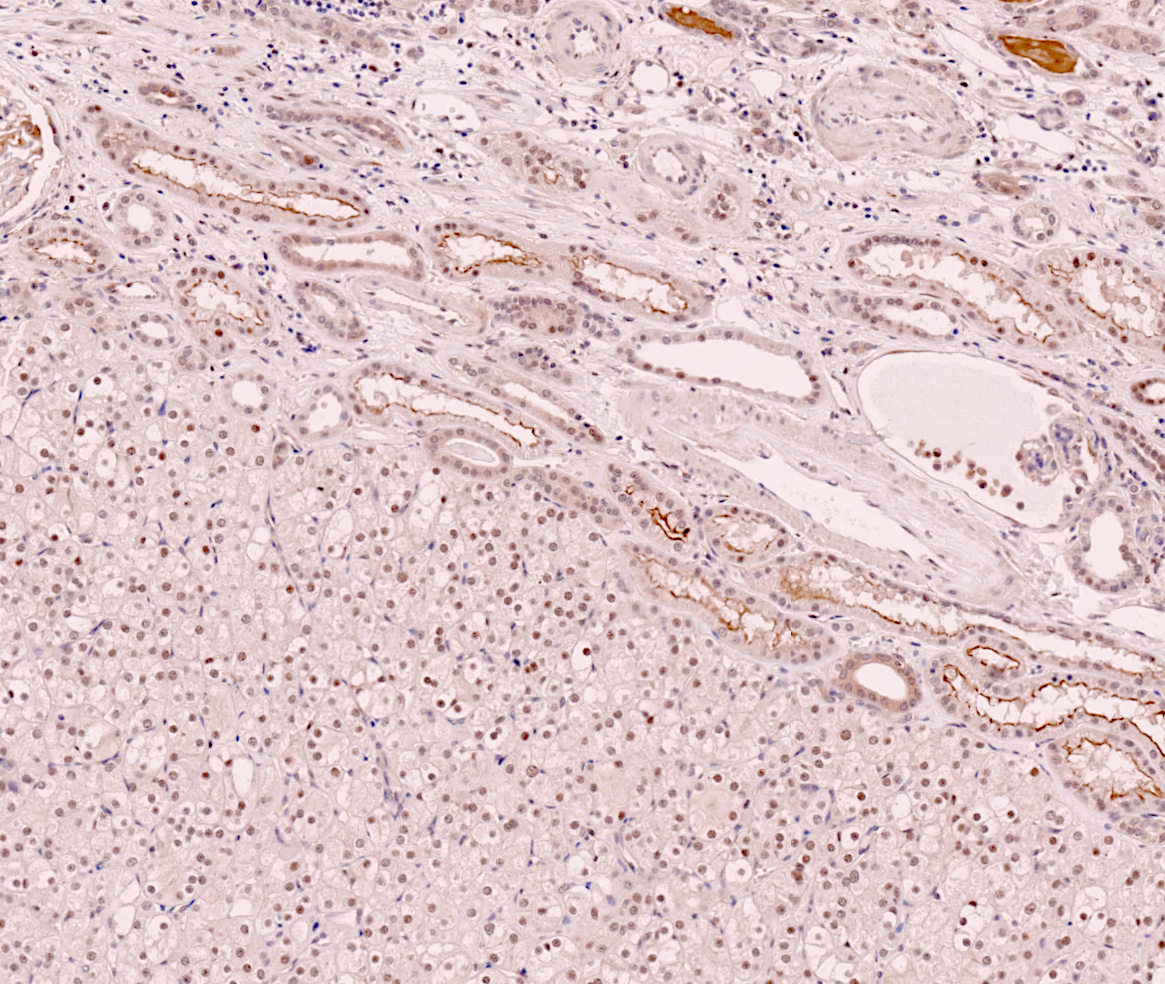

Supplement: Supplementary file 16 — Source Data for Figure 2 [file EMMM-15-e16877-s017.zip › Source_Data_Figure2/2A/TIF images for Figure 2A/BHD Patient 3 Tumor TFE3.tif]

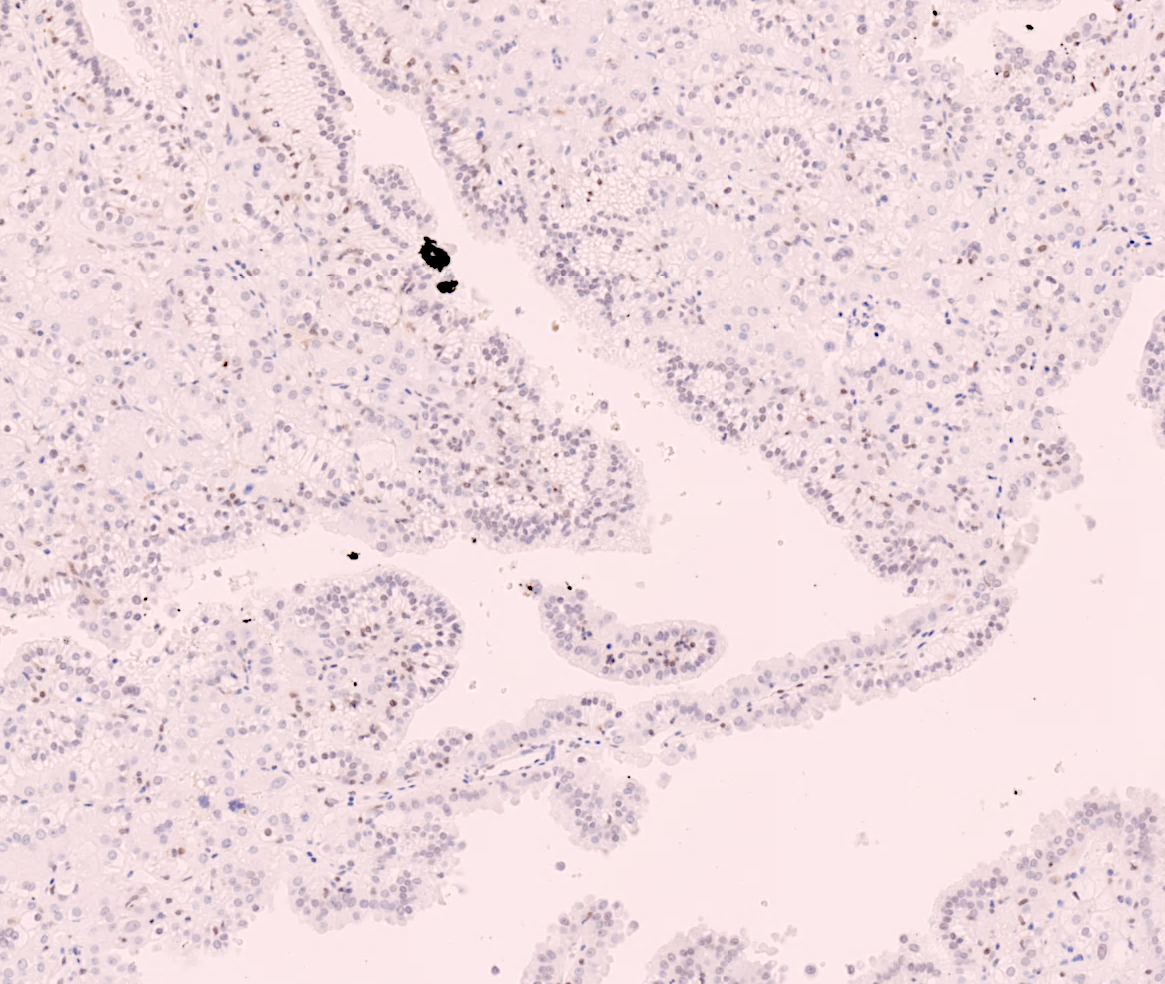

Supplement: Supplementary file 16 — Source Data for Figure 2 [file EMMM-15-e16877-s017.zip › Source_Data_Figure2/2A/TIF images for Figure 2A/BHD Patient 5 TFEB.tif]

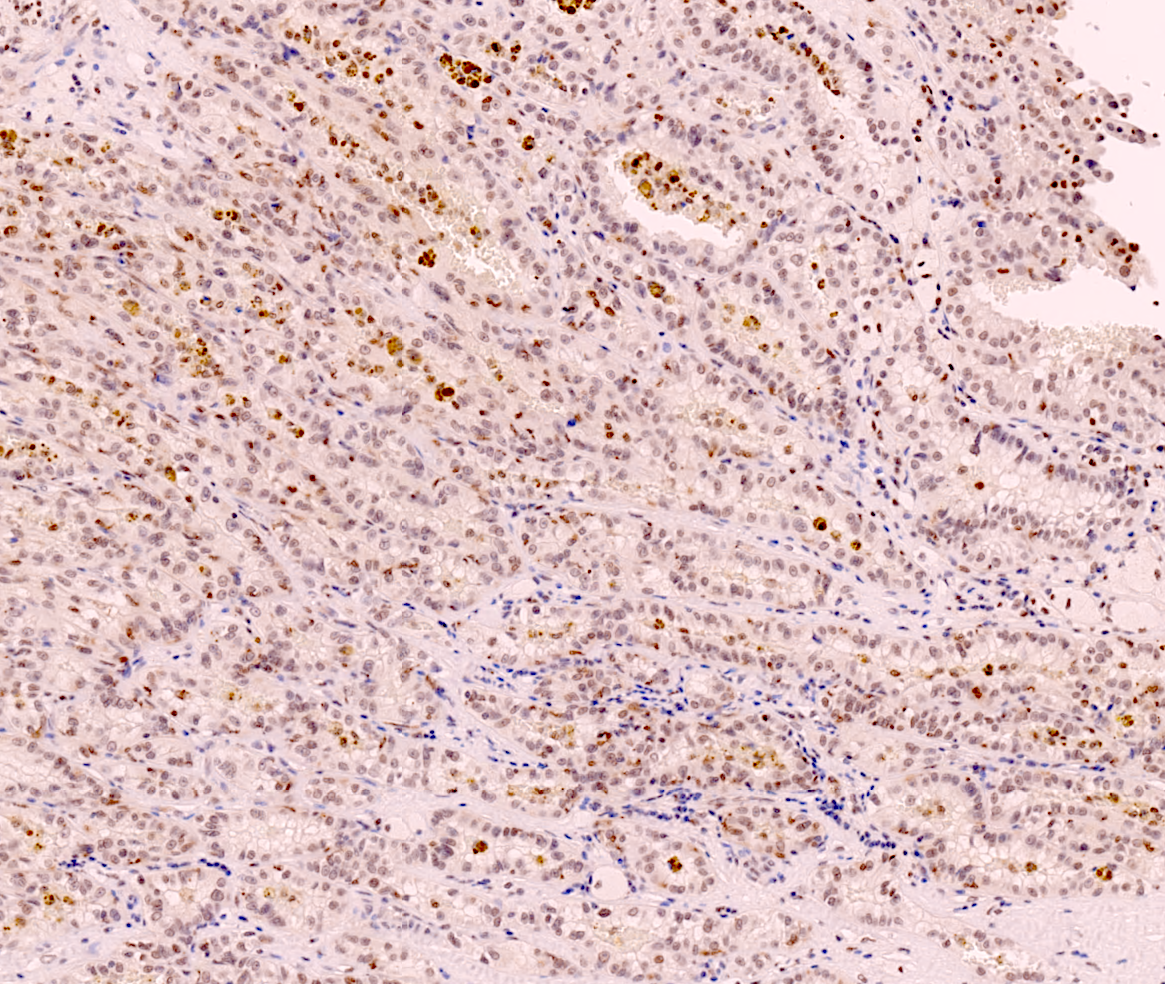

Supplement: Supplementary file 16 — Source Data for Figure 2 [file EMMM-15-e16877-s017.zip › Source_Data_Figure2/2A/TIF images for Figure 2A/BHD Patient 4 TFEB.tif]

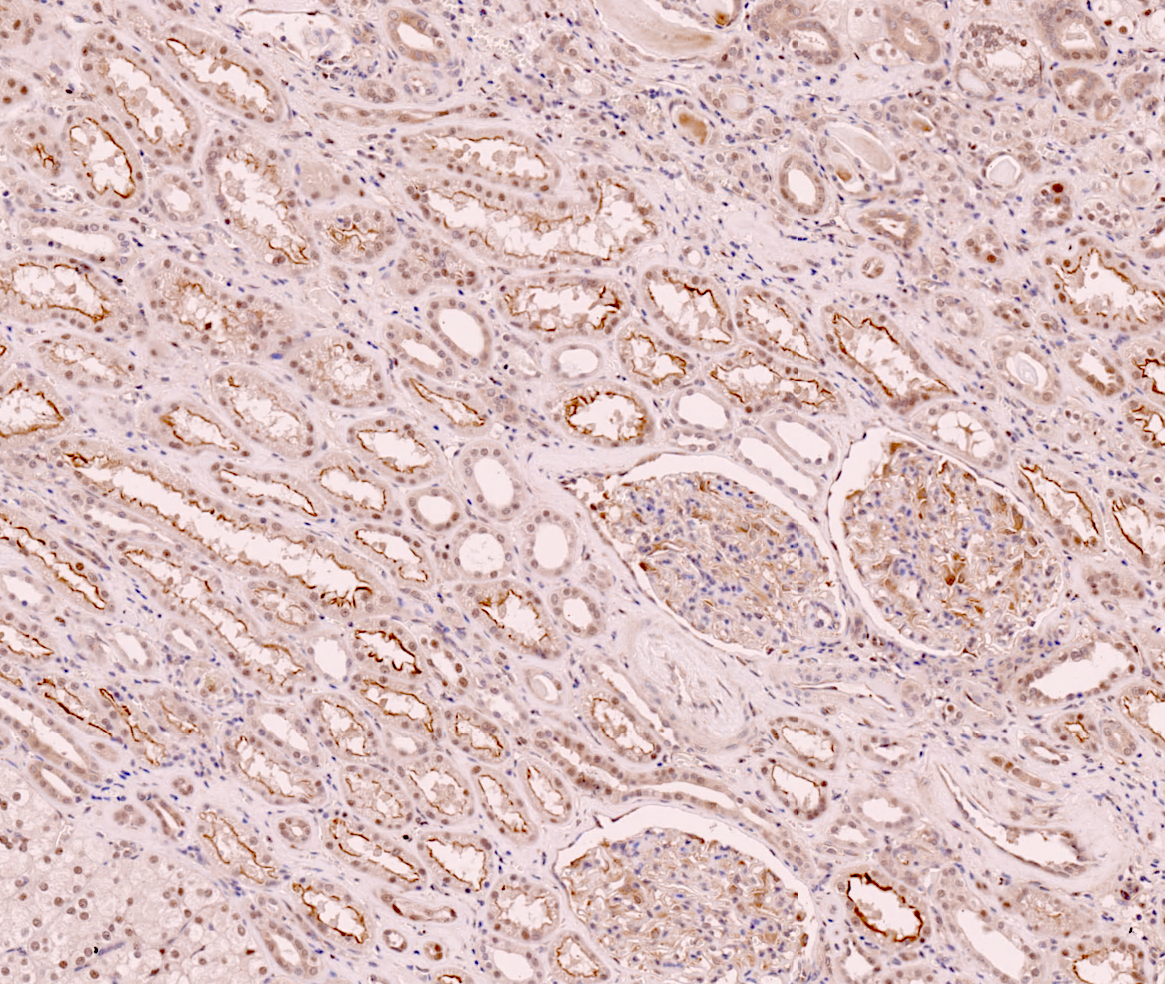

Supplement: Supplementary file 16 — Source Data for Figure 2 [file EMMM-15-e16877-s017.zip › Source_Data_Figure2/2A/TIF images for Figure 2A/BHD Patient 3 Normal TFE3.tif]

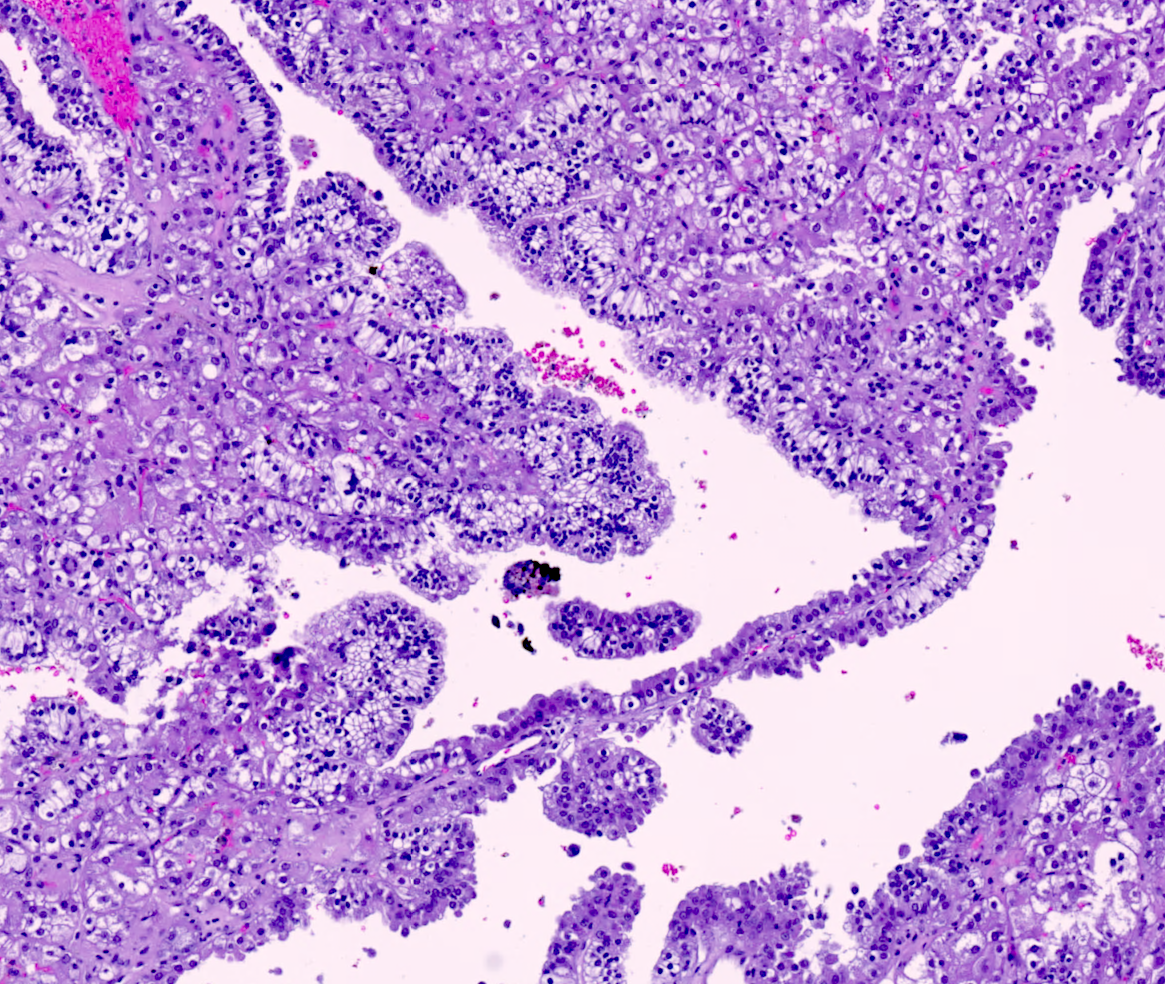

Supplement: Supplementary file 16 — Source Data for Figure 2 [file EMMM-15-e16877-s017.zip › Source_Data_Figure2/2A/TIF images for Figure 2A/BHD Patient 5 H&E.tif]

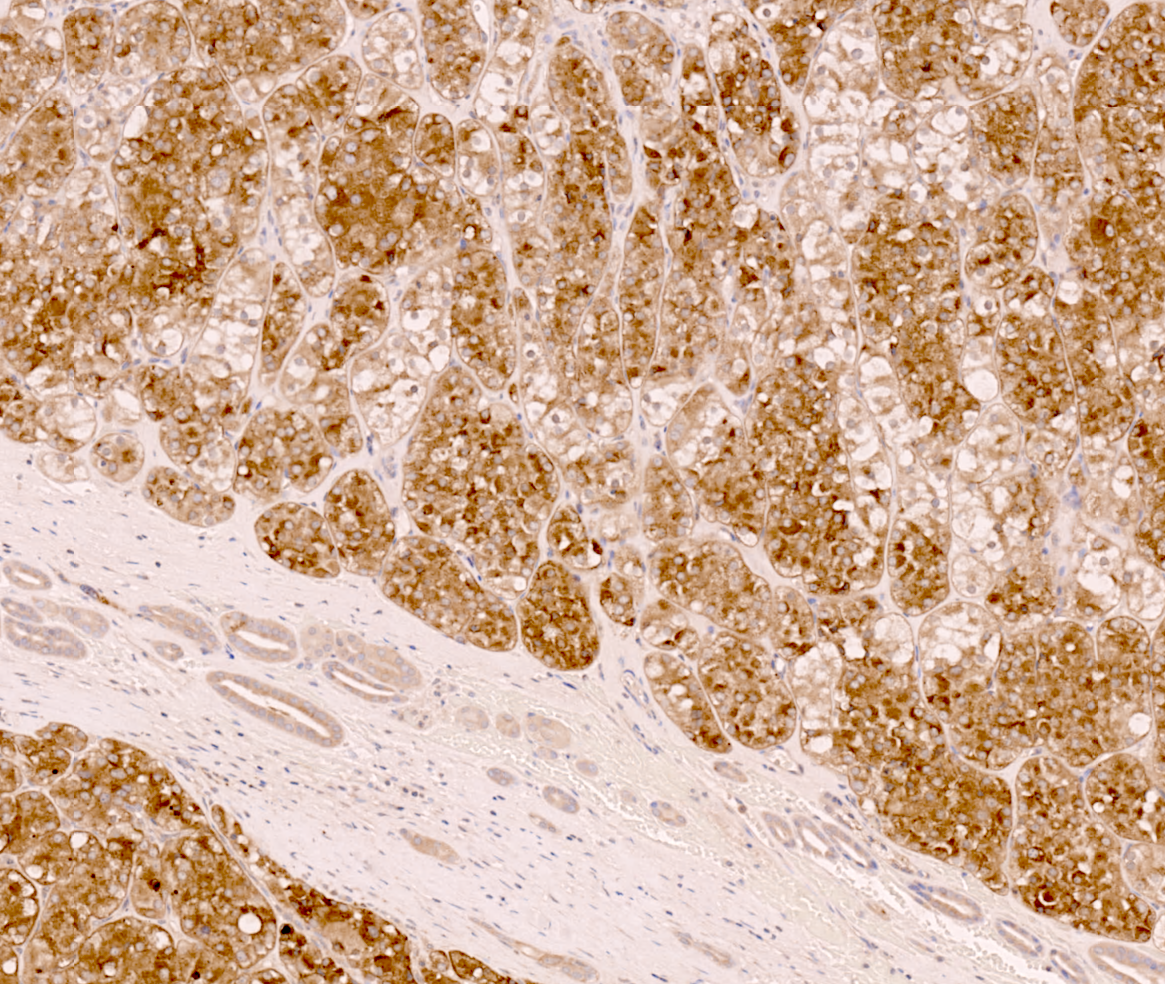

Supplement: Supplementary file 16 — Source Data for Figure 2 [file EMMM-15-e16877-s017.zip › Source_Data_Figure2/2A/TIF images for Figure 2A/BHD Patient 2 GPNMB.tif]

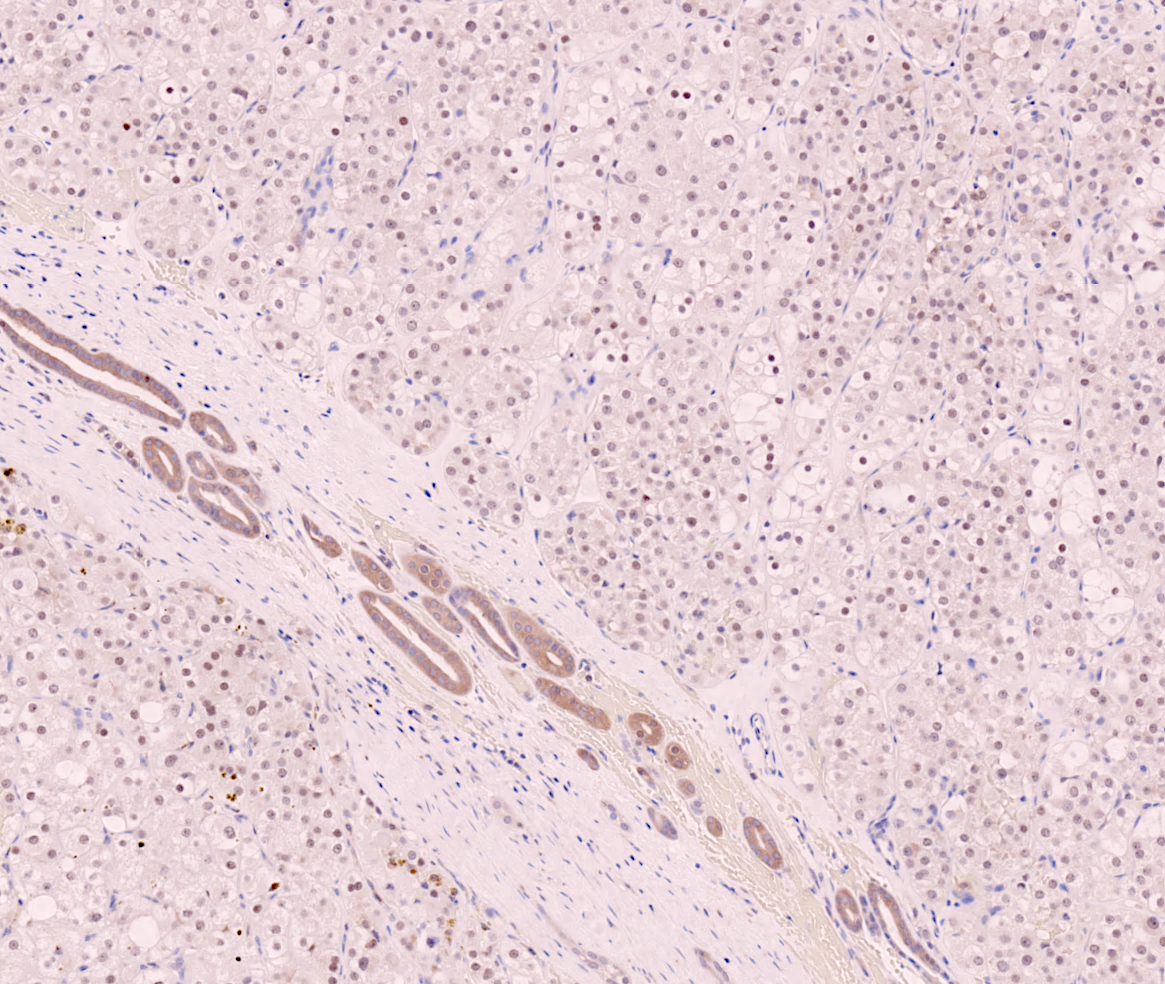

Supplement: Supplementary file 16 — Source Data for Figure 2 [file EMMM-15-e16877-s017.zip › Source_Data_Figure2/2A/TIF images for Figure 2A/BHD Patient 2 TFEB.tif]

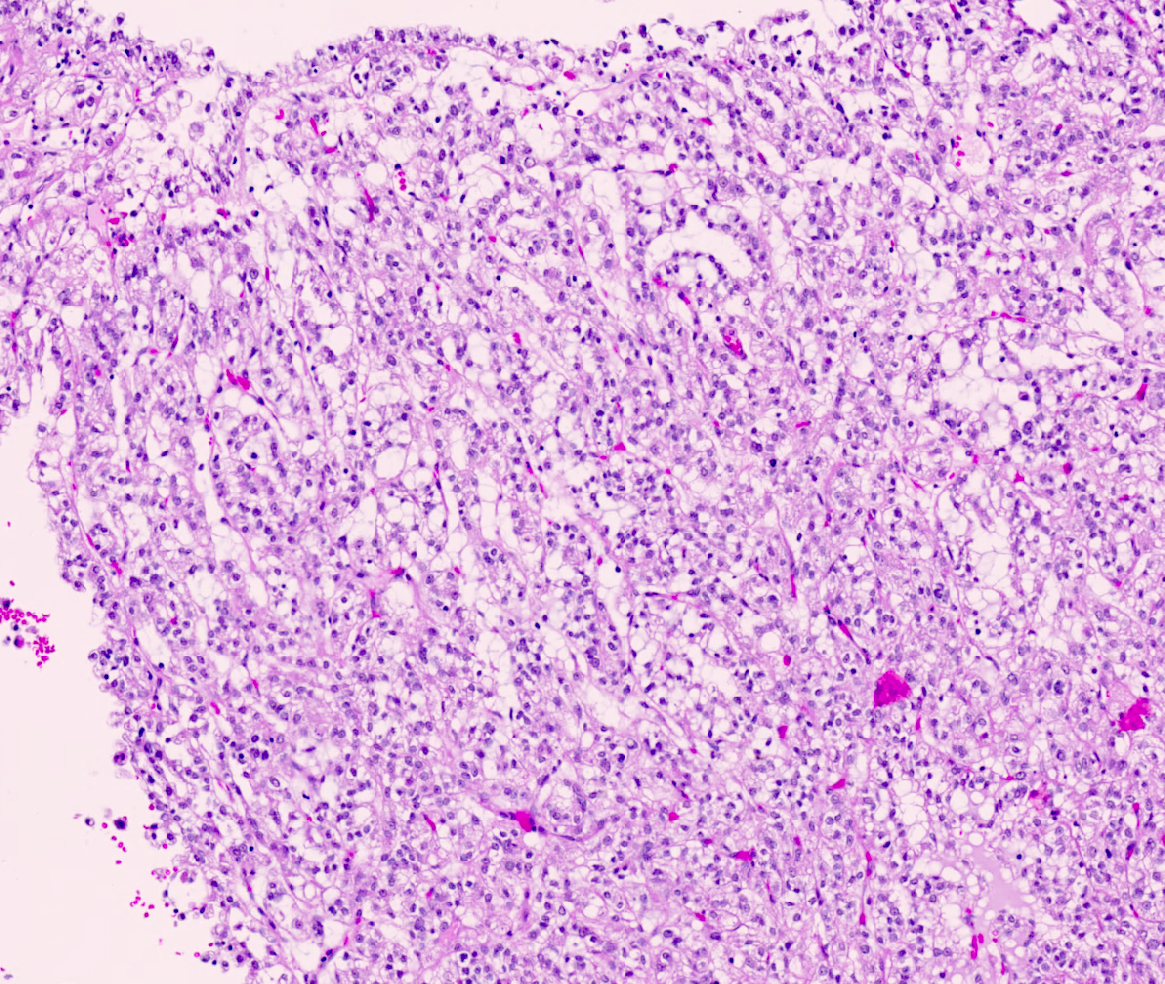

Supplement: Supplementary file 16 — Source Data for Figure 2 [file EMMM-15-e16877-s017.zip › Source_Data_Figure2/2A/TIF images for Figure 2A/BHD Patient 7 H&E.tif]

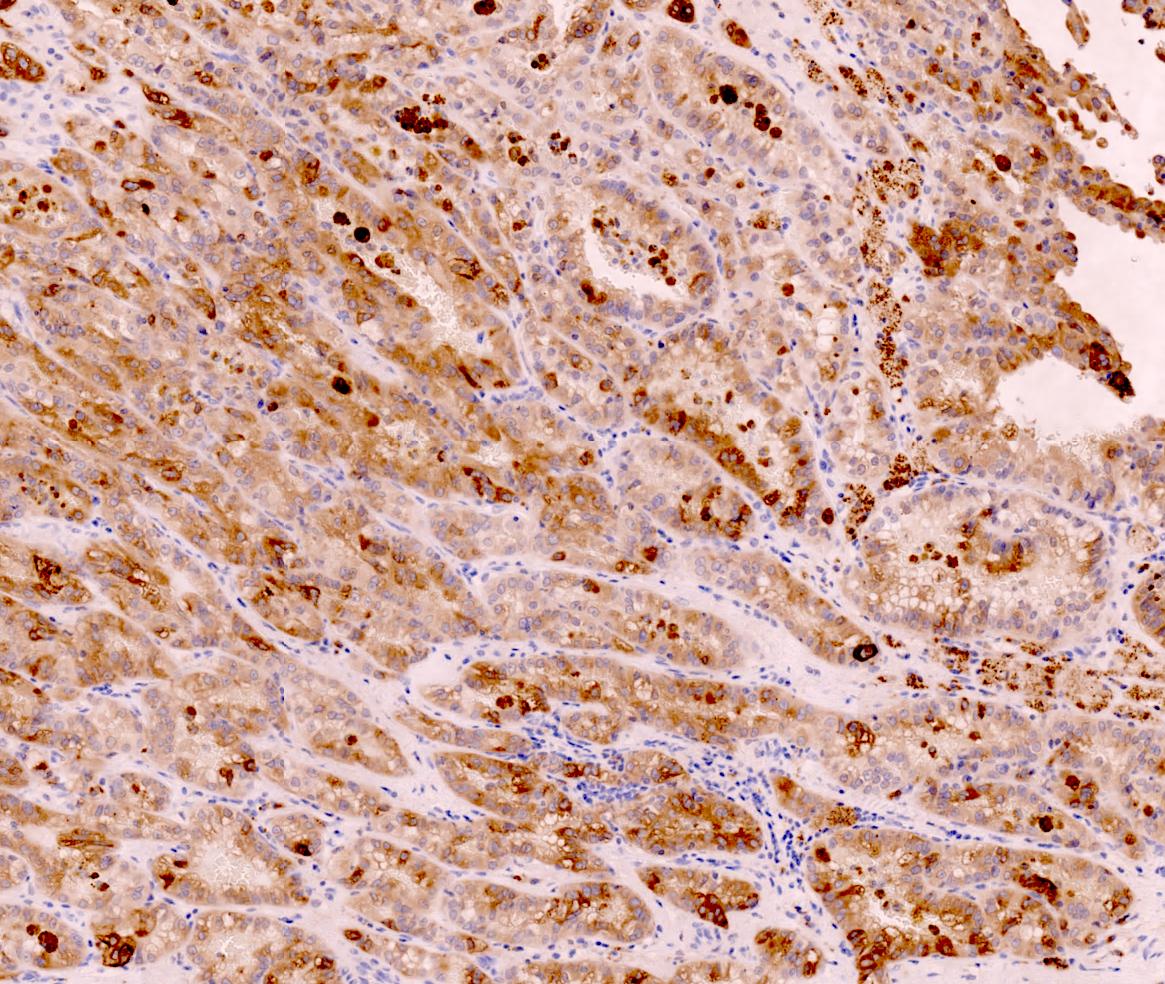

Supplement: Supplementary file 16 — Source Data for Figure 2 [file EMMM-15-e16877-s017.zip › Source_Data_Figure2/2A/TIF images for Figure 2A/BHD Patient 4 GPNMB.tif]

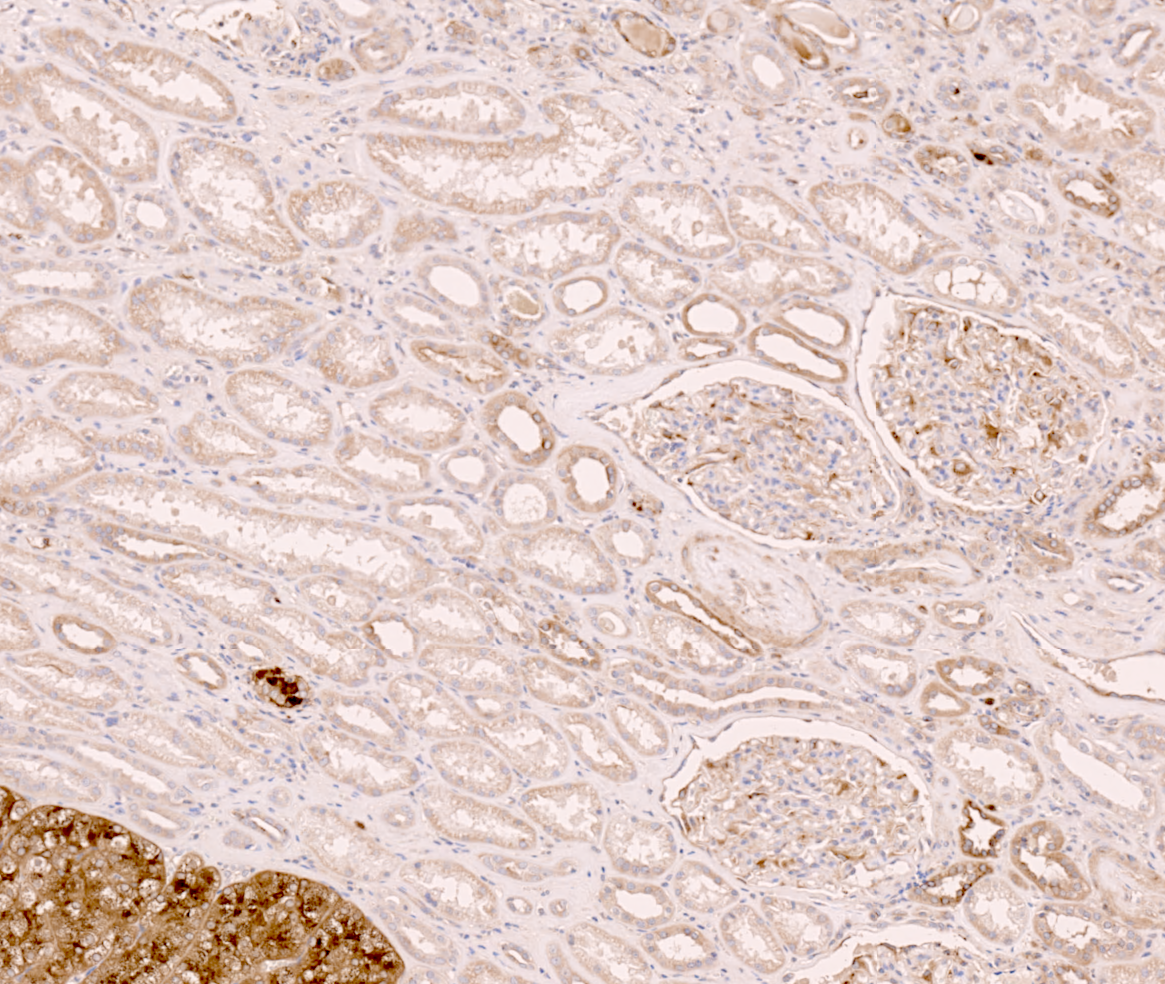

Supplement: Supplementary file 16 — Source Data for Figure 2 [file EMMM-15-e16877-s017.zip › Source_Data_Figure2/2A/TIF images for Figure 2A/BHD Patient 3 Normal GPNMB.tif]

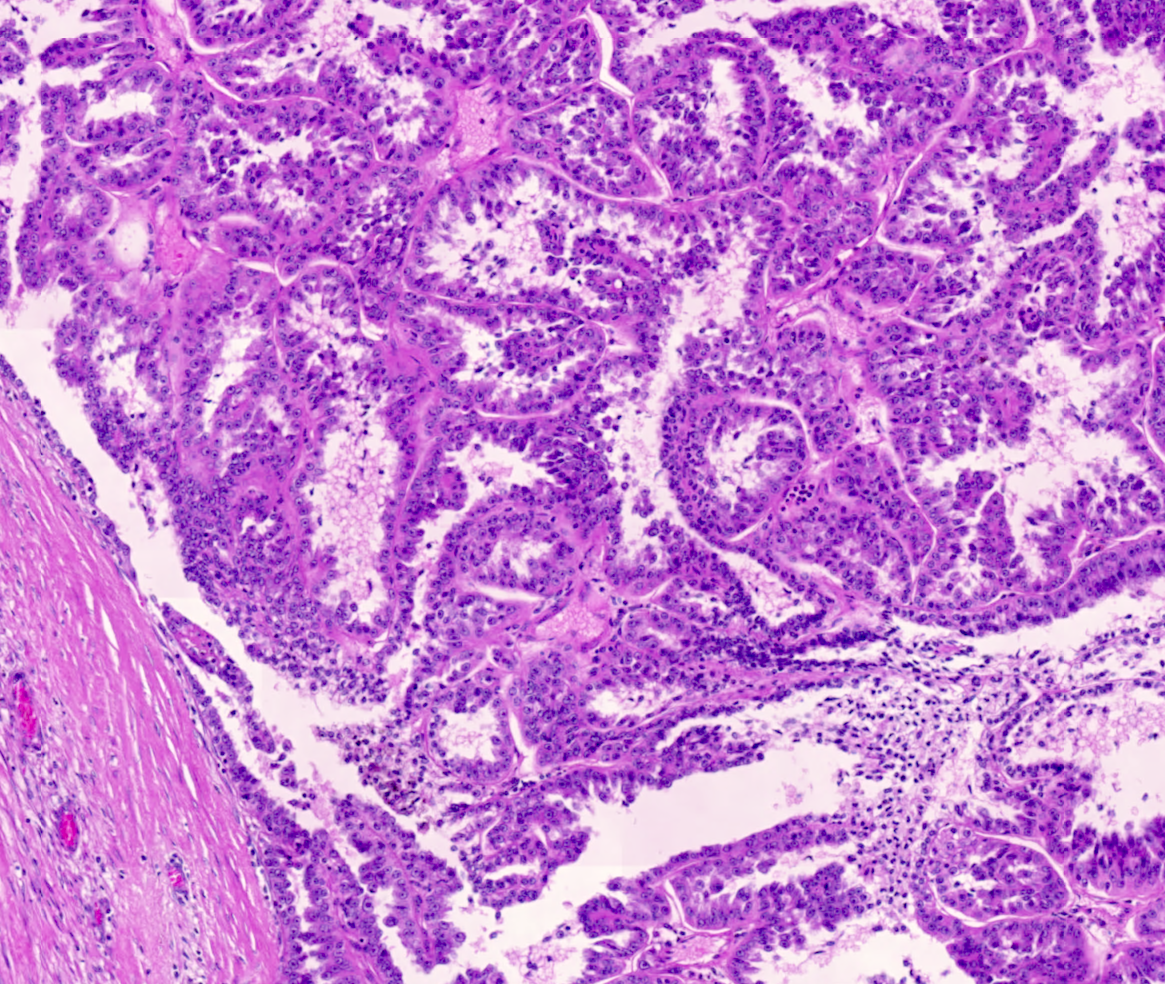

Supplement: Supplementary file 16 — Source Data for Figure 2 [file EMMM-15-e16877-s017.zip › Source_Data_Figure2/2A/TIF images for Figure 2A/BHD Patient 6 H&E.tif]

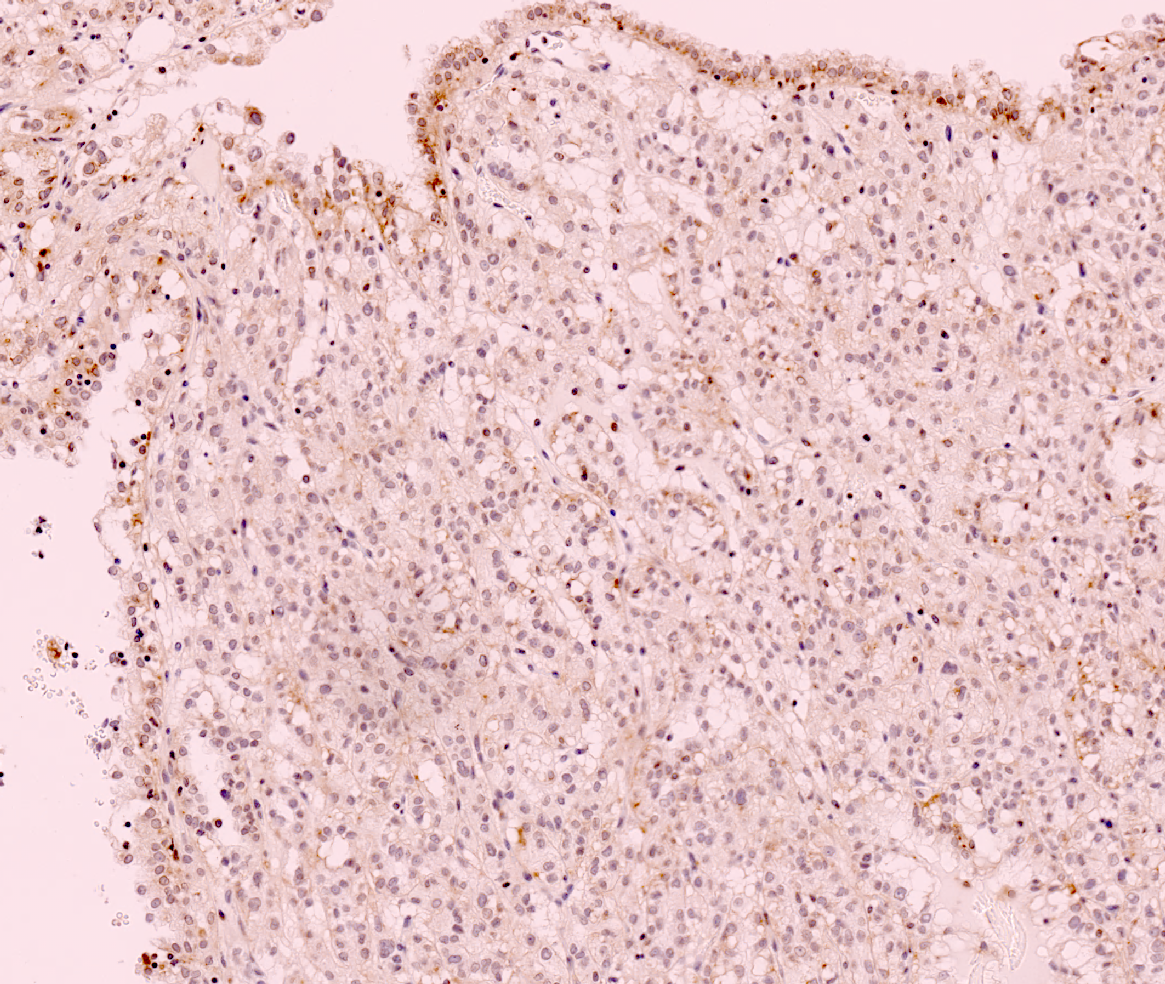

Supplement: Supplementary file 16 — Source Data for Figure 2 [file EMMM-15-e16877-s017.zip › Source_Data_Figure2/2A/TIF images for Figure 2A/BHD Patient 7 NPC1.tif]

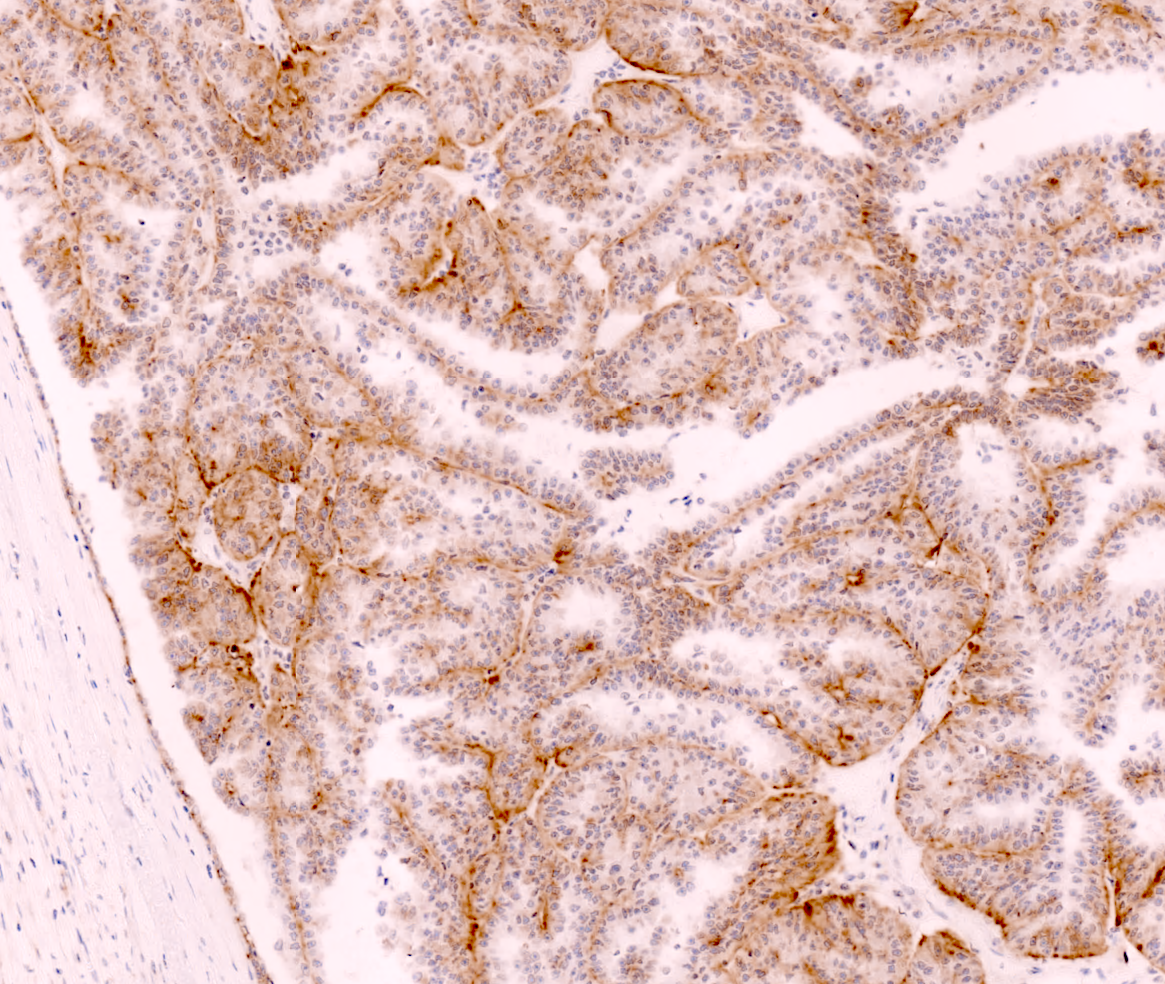

Supplement: Supplementary file 16 — Source Data for Figure 2 [file EMMM-15-e16877-s017.zip › Source_Data_Figure2/2A/TIF images for Figure 2A/BHD Patient 6 NPC1.tif]

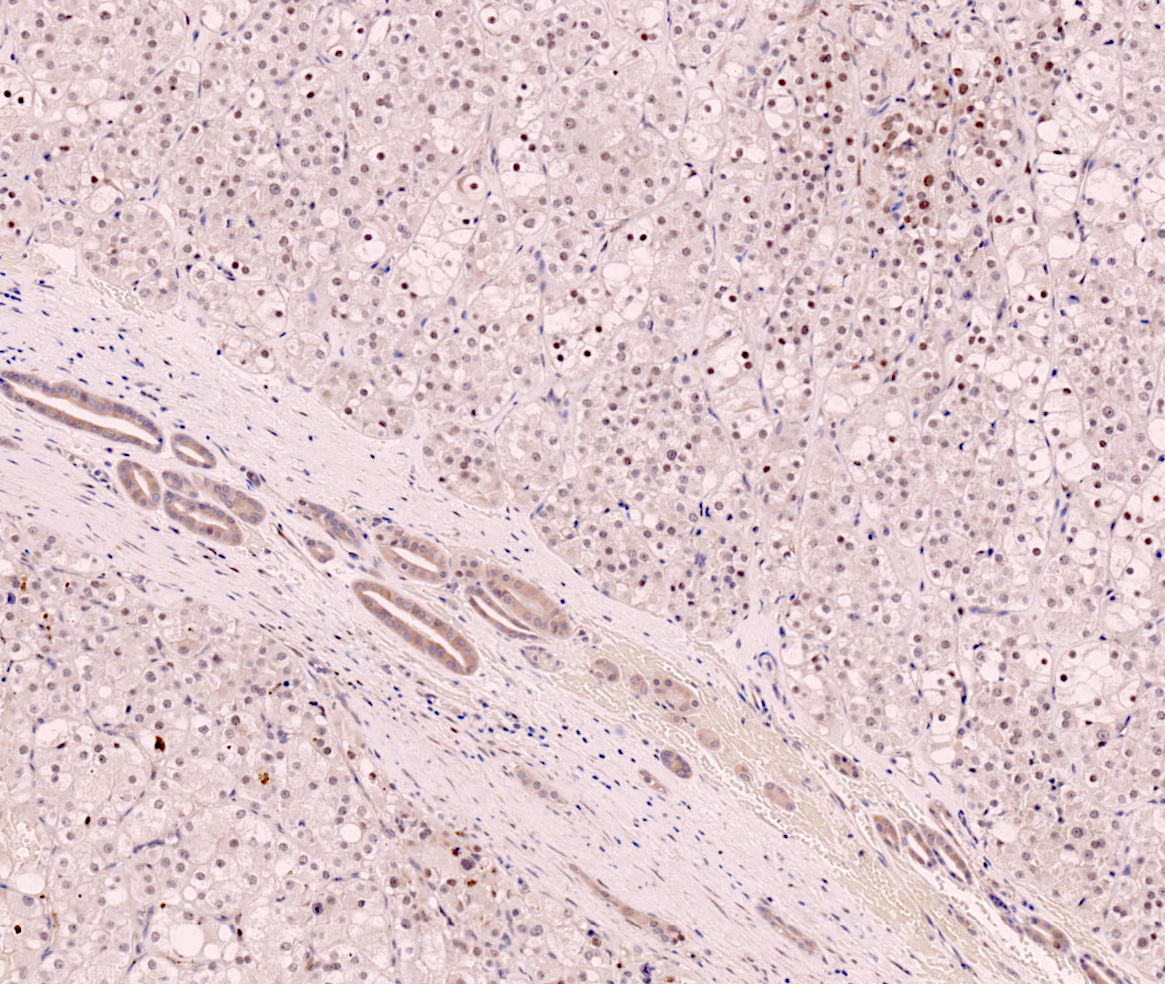

Supplement: Supplementary file 16 — Source Data for Figure 2 [file EMMM-15-e16877-s017.zip › Source_Data_Figure2/2A/TIF images for Figure 2A/BHD Patient 2 TFE3.tif]

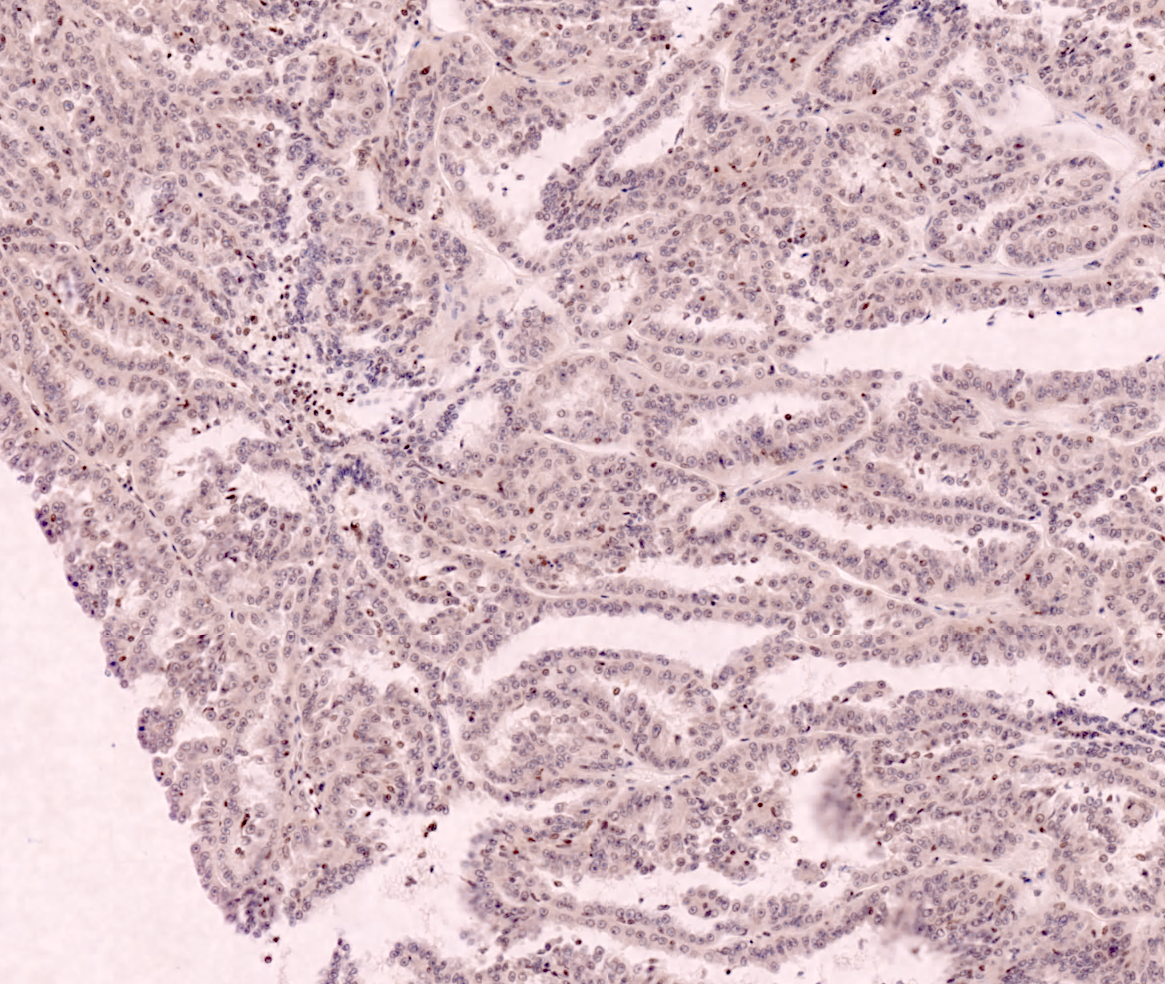

Supplement: Supplementary file 16 — Source Data for Figure 2 [file EMMM-15-e16877-s017.zip › Source_Data_Figure2/2A/TIF images for Figure 2A/BHD Patient 6 TFEB.tif]

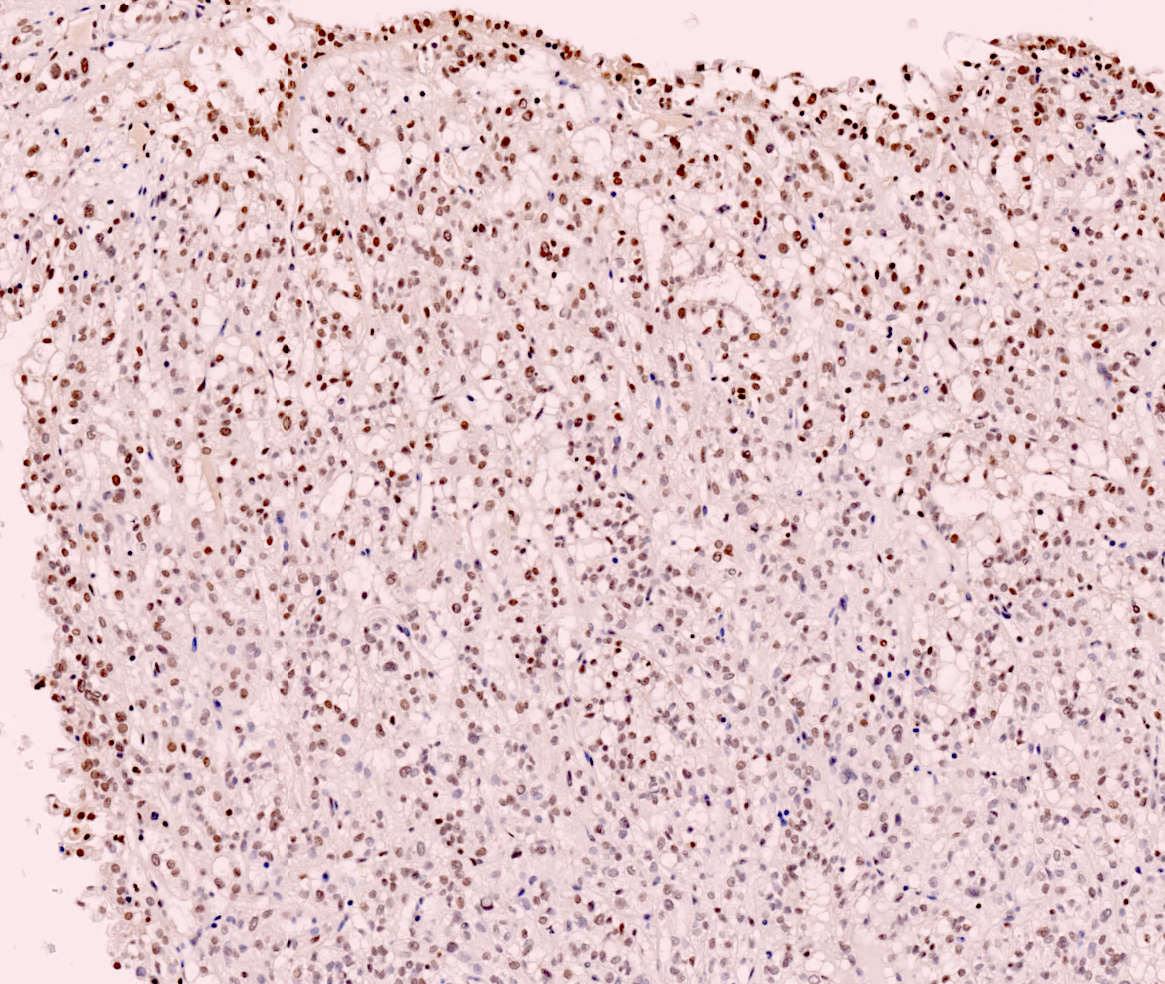

Supplement: Supplementary file 16 — Source Data for Figure 2 [file EMMM-15-e16877-s017.zip › Source_Data_Figure2/2A/TIF images for Figure 2A/BHD Patient 7 TFEB.tif]

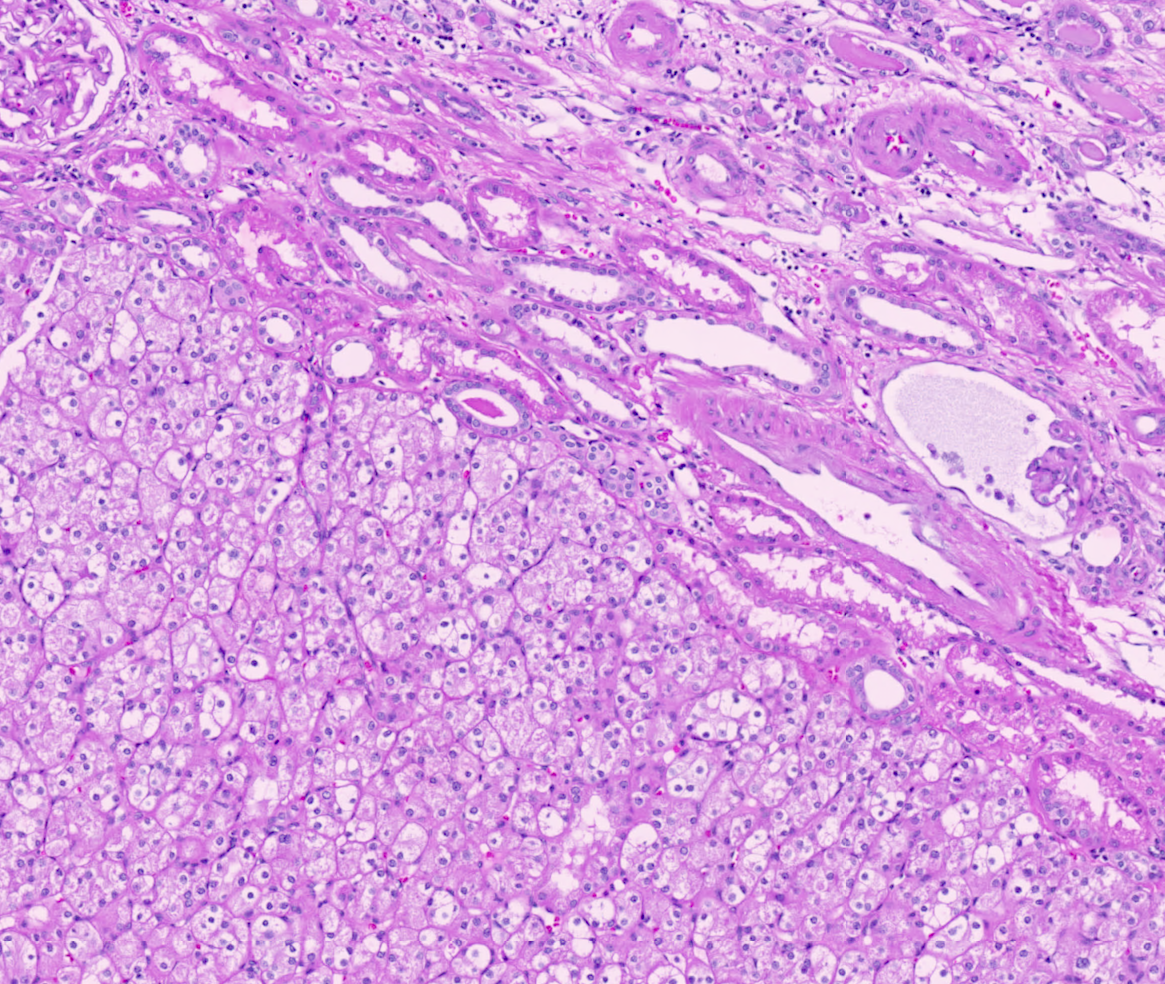

Supplement: Supplementary file 16 — Source Data for Figure 2 [file EMMM-15-e16877-s017.zip › Source_Data_Figure2/2A/TIF images for Figure 2A/BHD Patient 3 Tumor H&E.tif]

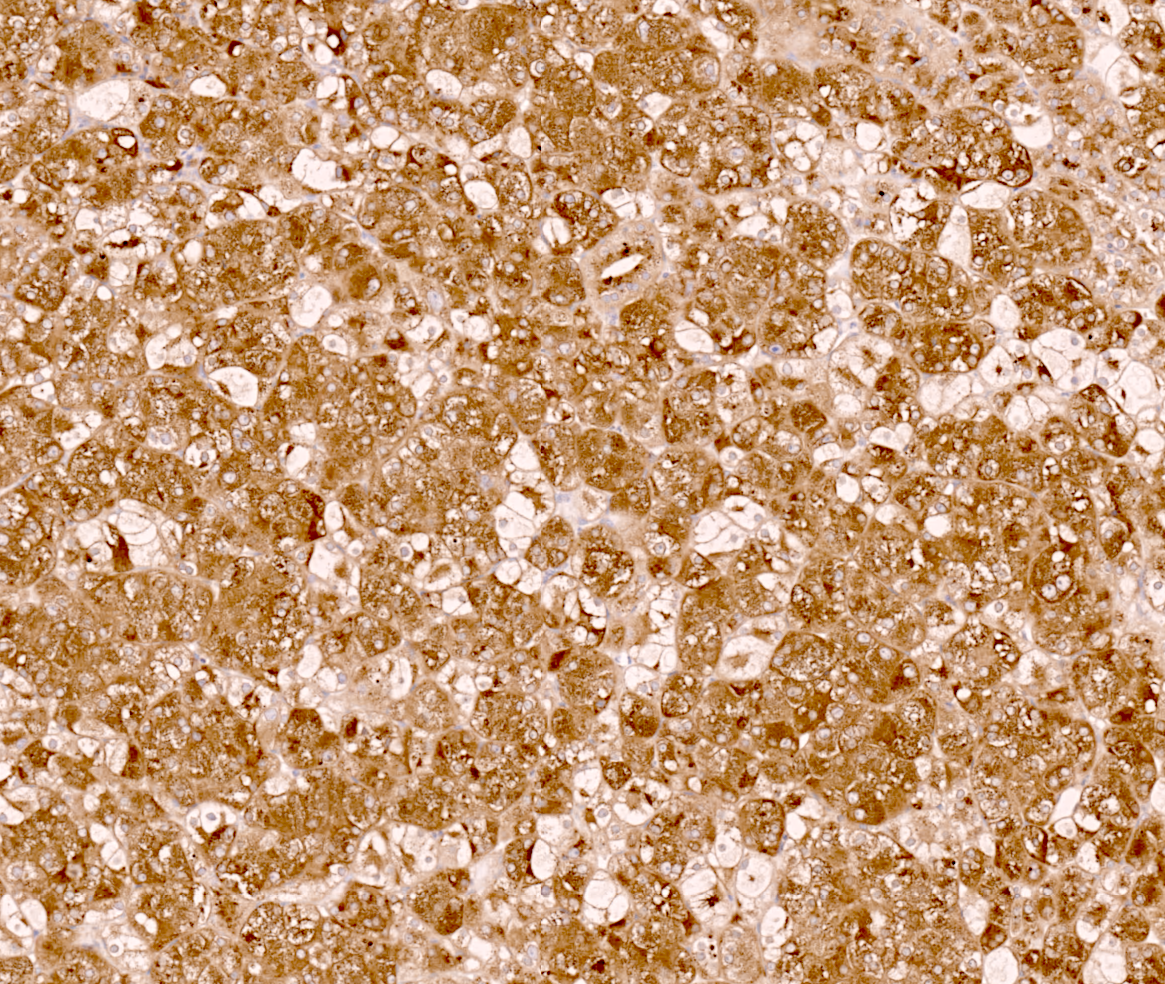

Supplement: Supplementary file 16 — Source Data for Figure 2 [file EMMM-15-e16877-s017.zip › Source_Data_Figure2/2A/TIF images for Figure 2A/BHD Patient 1 GPNMB.tif]

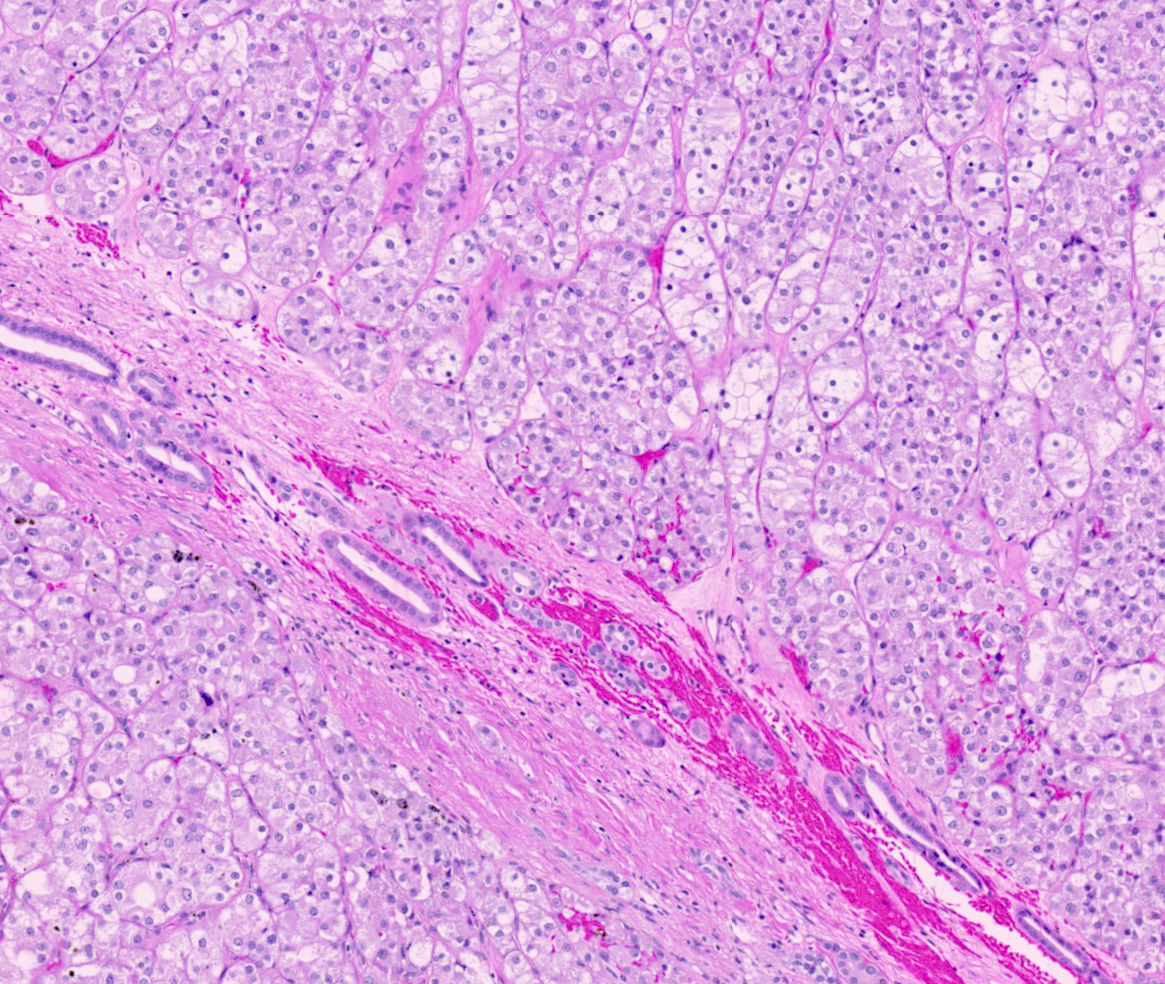

Supplement: Supplementary file 16 — Source Data for Figure 2 [file EMMM-15-e16877-s017.zip › Source_Data_Figure2/2A/TIF images for Figure 2A/BHD Patient 2 H&E.tif]

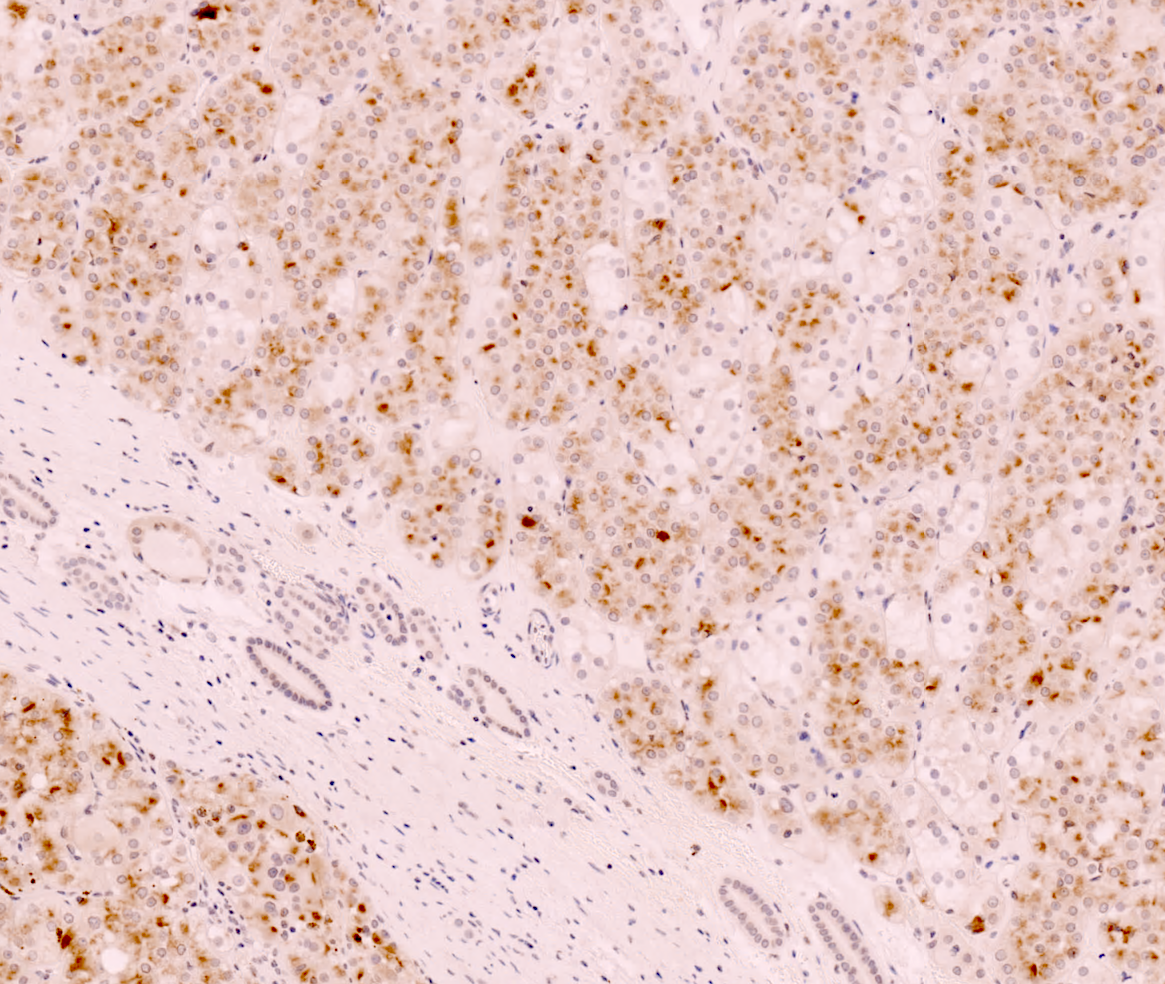

Supplement: Supplementary file 16 — Source Data for Figure 2 [file EMMM-15-e16877-s017.zip › Source_Data_Figure2/2A/TIF images for Figure 2A/BHD Patient 2 NPC1.tif]

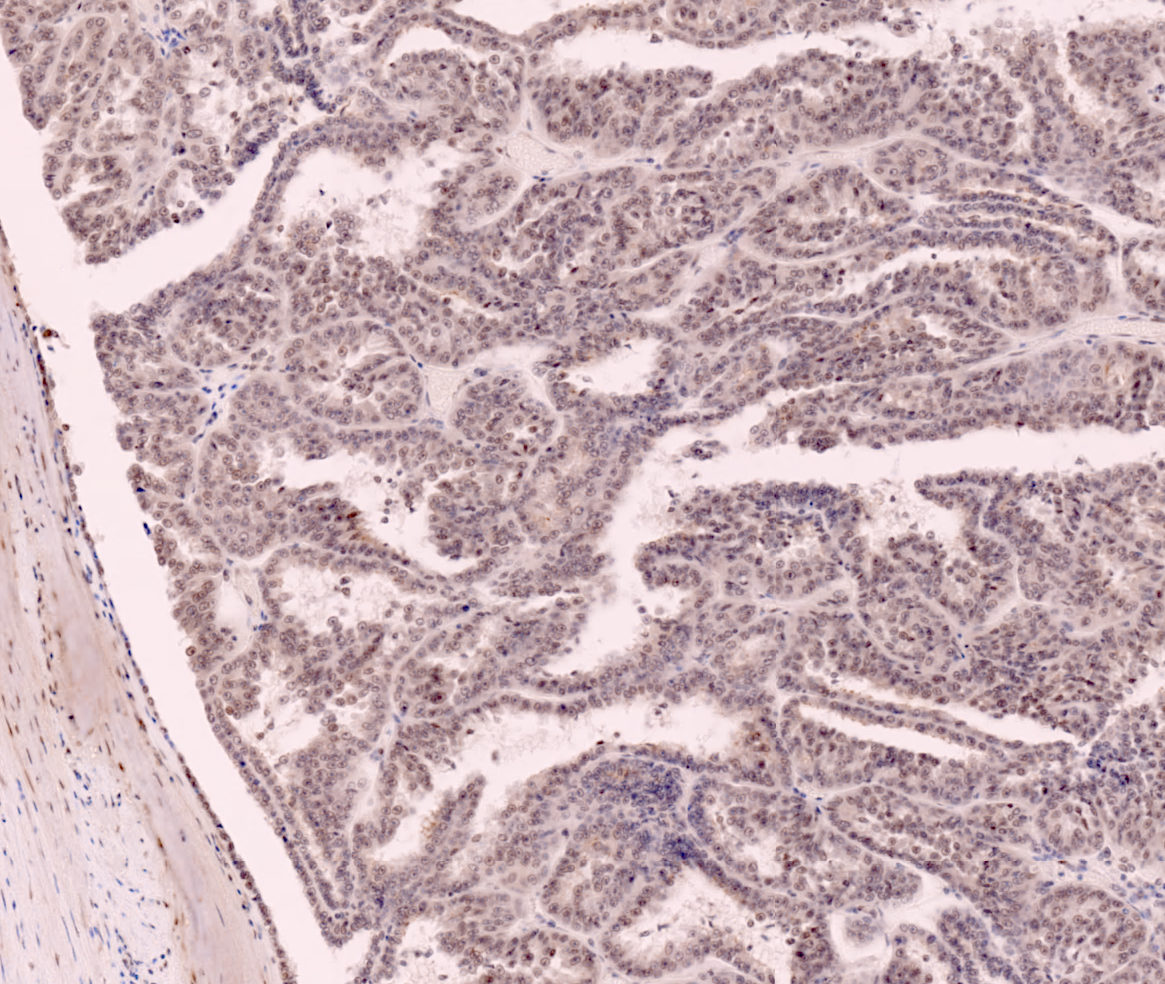

Supplement: Supplementary file 16 — Source Data for Figure 2 [file EMMM-15-e16877-s017.zip › Source_Data_Figure2/2A/TIF images for Figure 2A/BHD Patient 6 TFE3.tif]

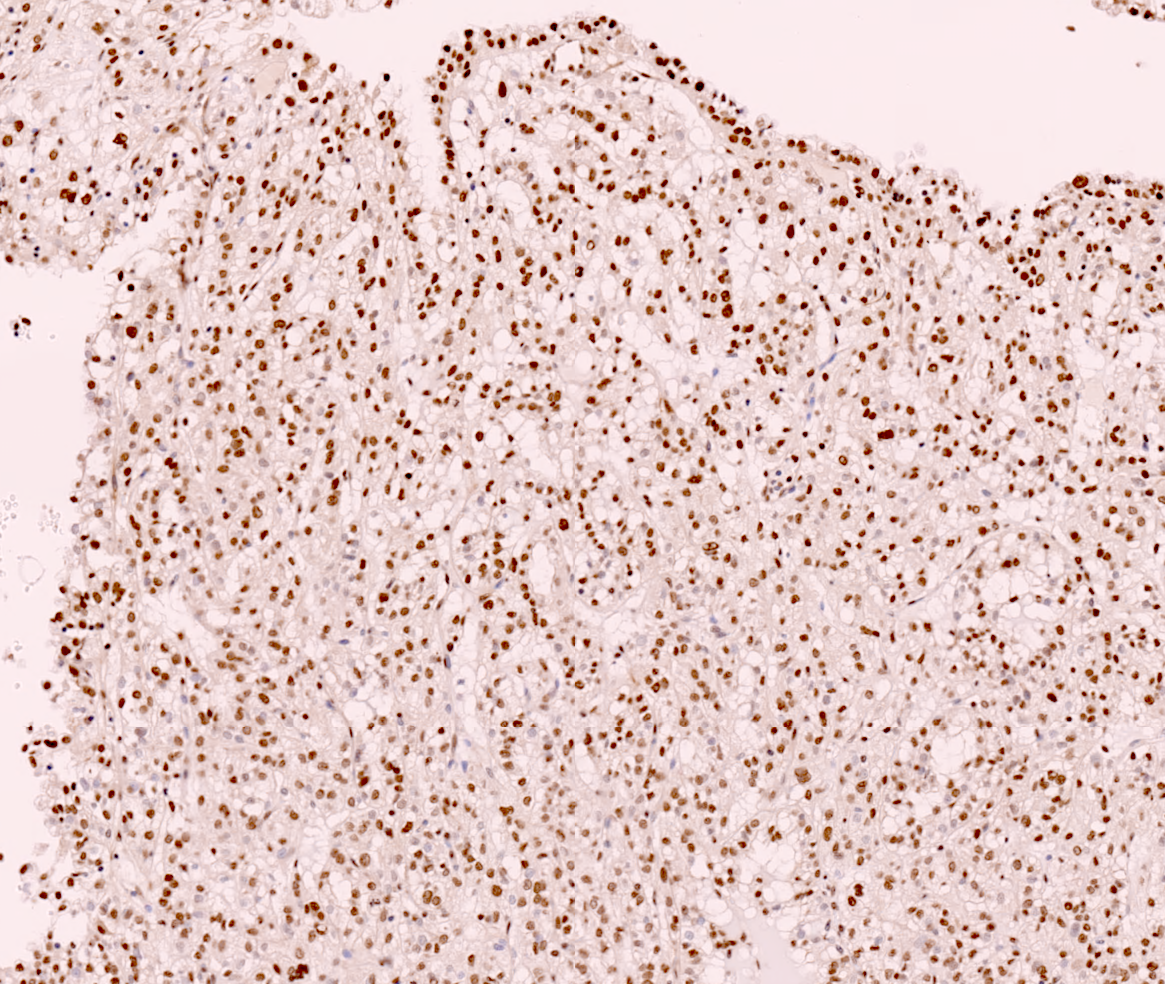

Supplement: Supplementary file 16 — Source Data for Figure 2 [file EMMM-15-e16877-s017.zip › Source_Data_Figure2/2A/TIF images for Figure 2A/BHD Patient 7 TFE3.tif]

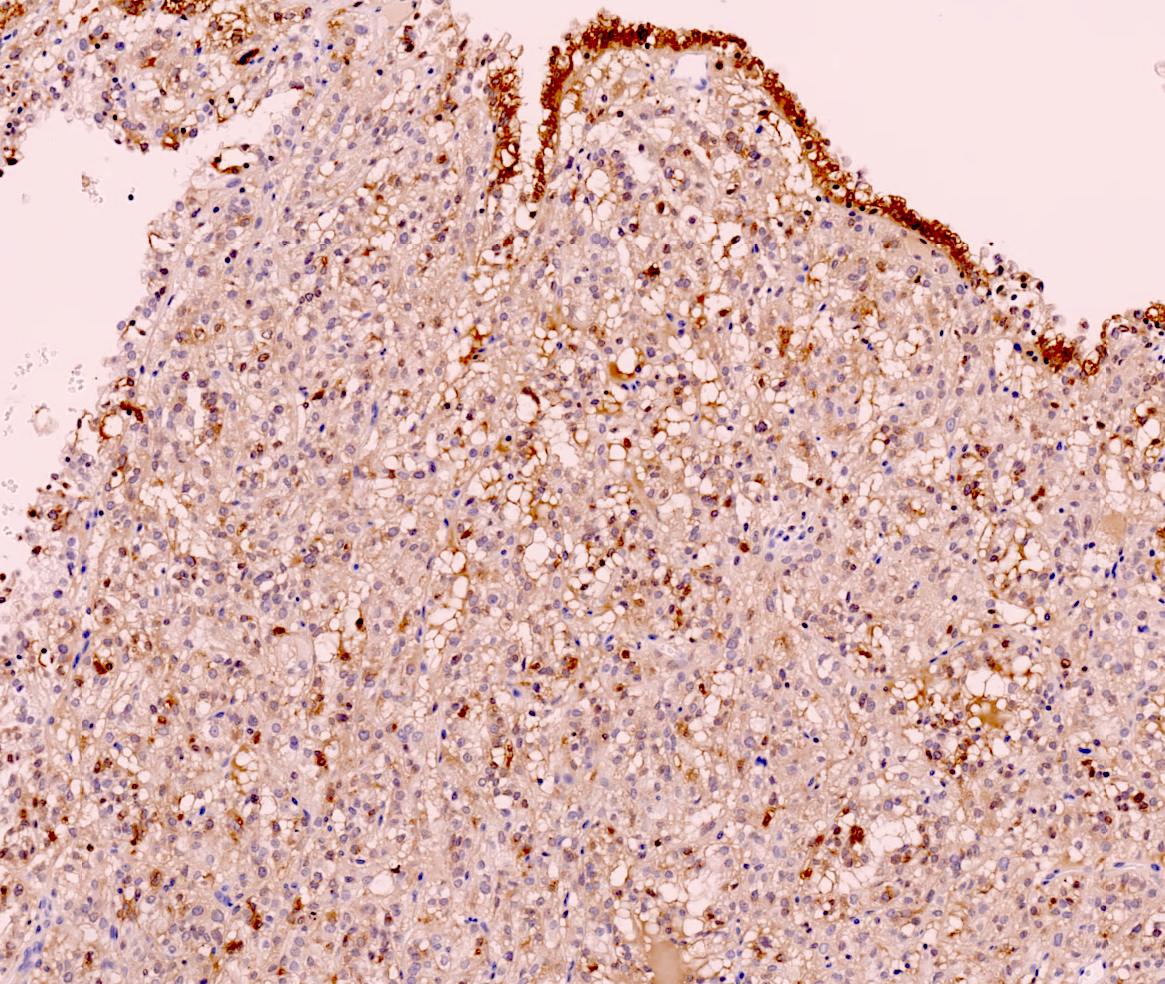

Supplement: Supplementary file 16 — Source Data for Figure 2 [file EMMM-15-e16877-s017.zip › Source_Data_Figure2/2A/TIF images for Figure 2A/BHD Patient 7 GPNMB.tif]

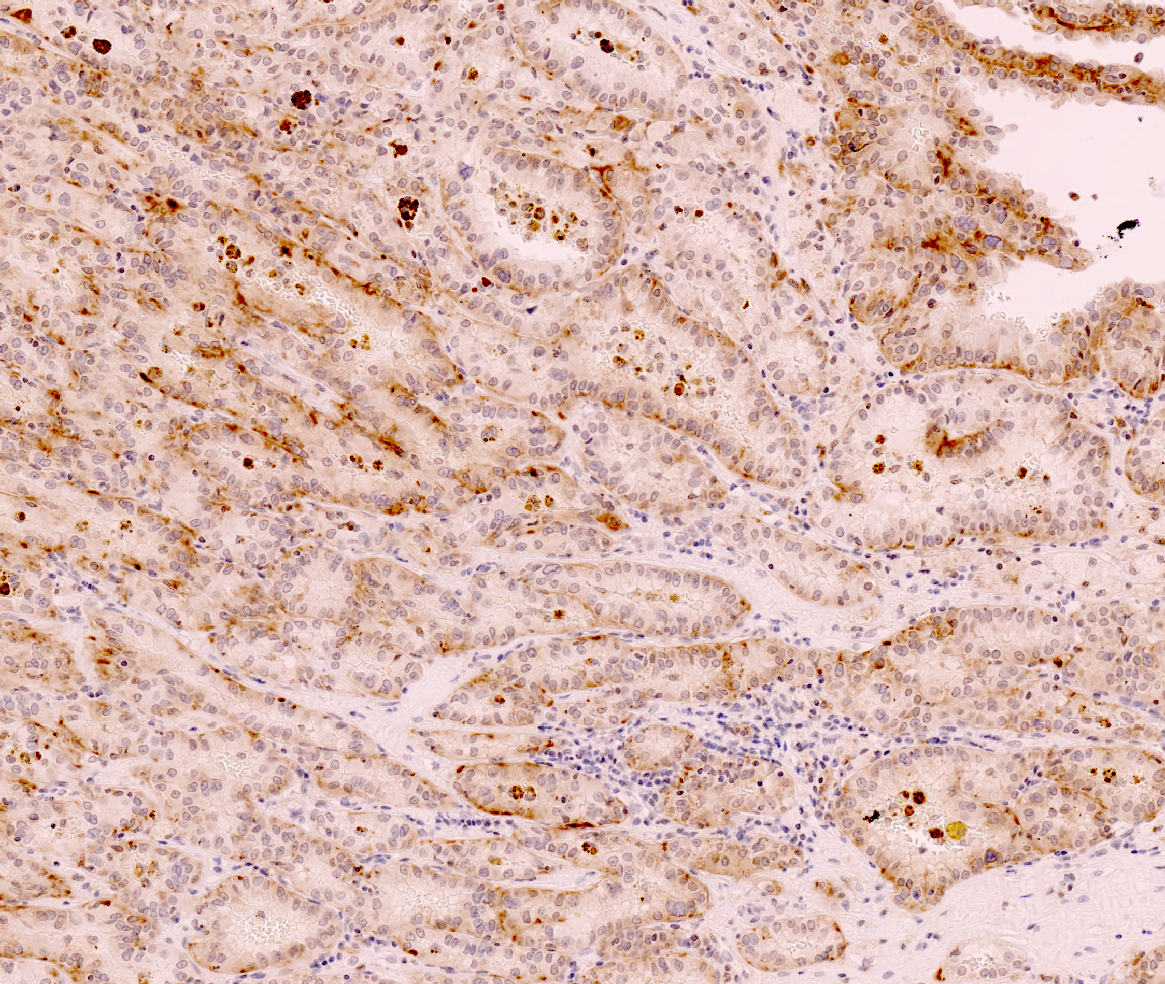

Supplement: Supplementary file 16 — Source Data for Figure 2 [file EMMM-15-e16877-s017.zip › Source_Data_Figure2/2A/TIF images for Figure 2A/BHD Patient 4 NPC1.tif]

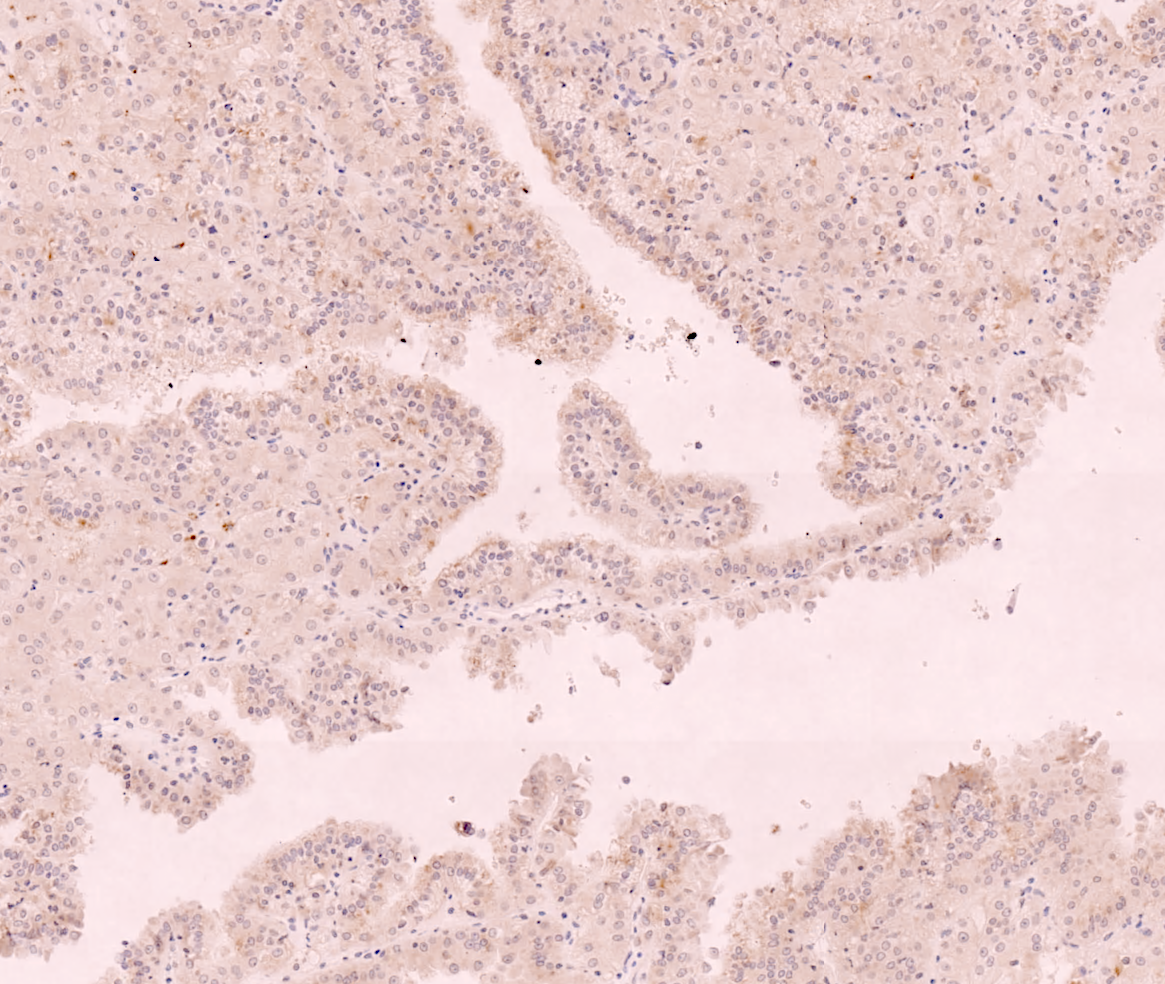

Supplement: Supplementary file 16 — Source Data for Figure 2 [file EMMM-15-e16877-s017.zip › Source_Data_Figure2/2A/TIF images for Figure 2A/BHD Patient 5 NPC1.tif]

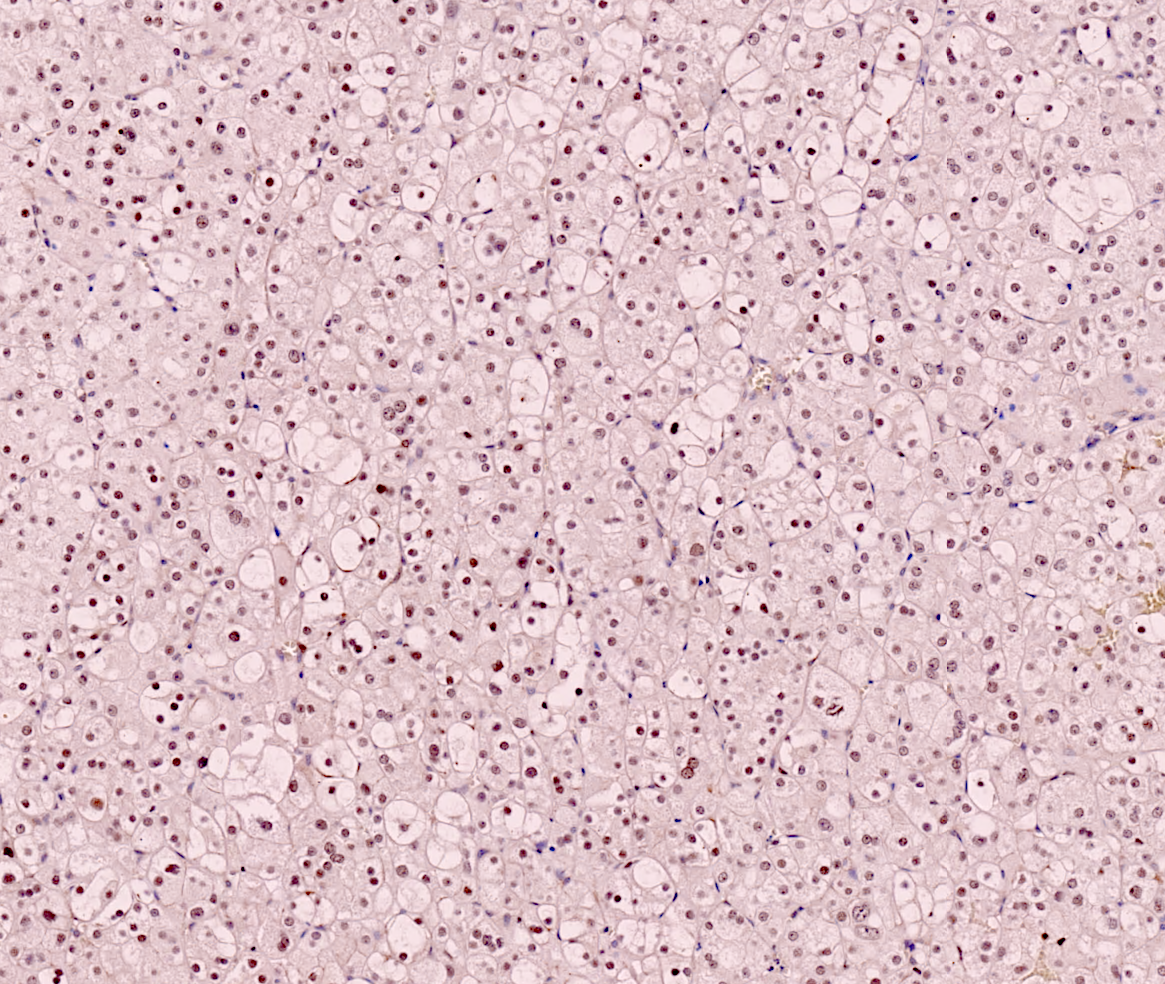

Supplement: Supplementary file 16 — Source Data for Figure 2 [file EMMM-15-e16877-s017.zip › Source_Data_Figure2/2A/TIF images for Figure 2A/BHD Patient 1 TFE3.tif]

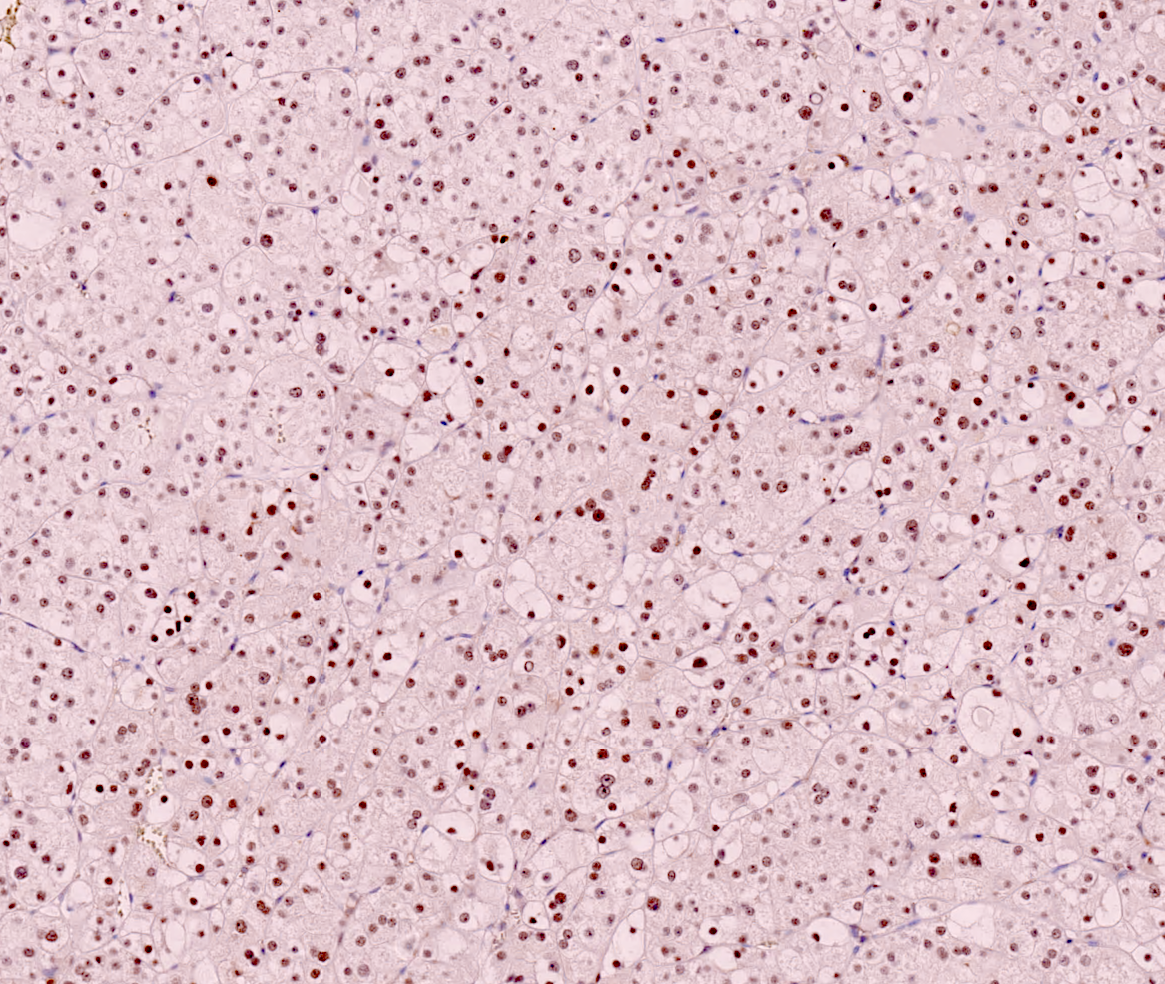

Supplement: Supplementary file 16 — Source Data for Figure 2 [file EMMM-15-e16877-s017.zip › Source_Data_Figure2/2A/TIF images for Figure 2A/BHD Patient 1 TFEB.tif]

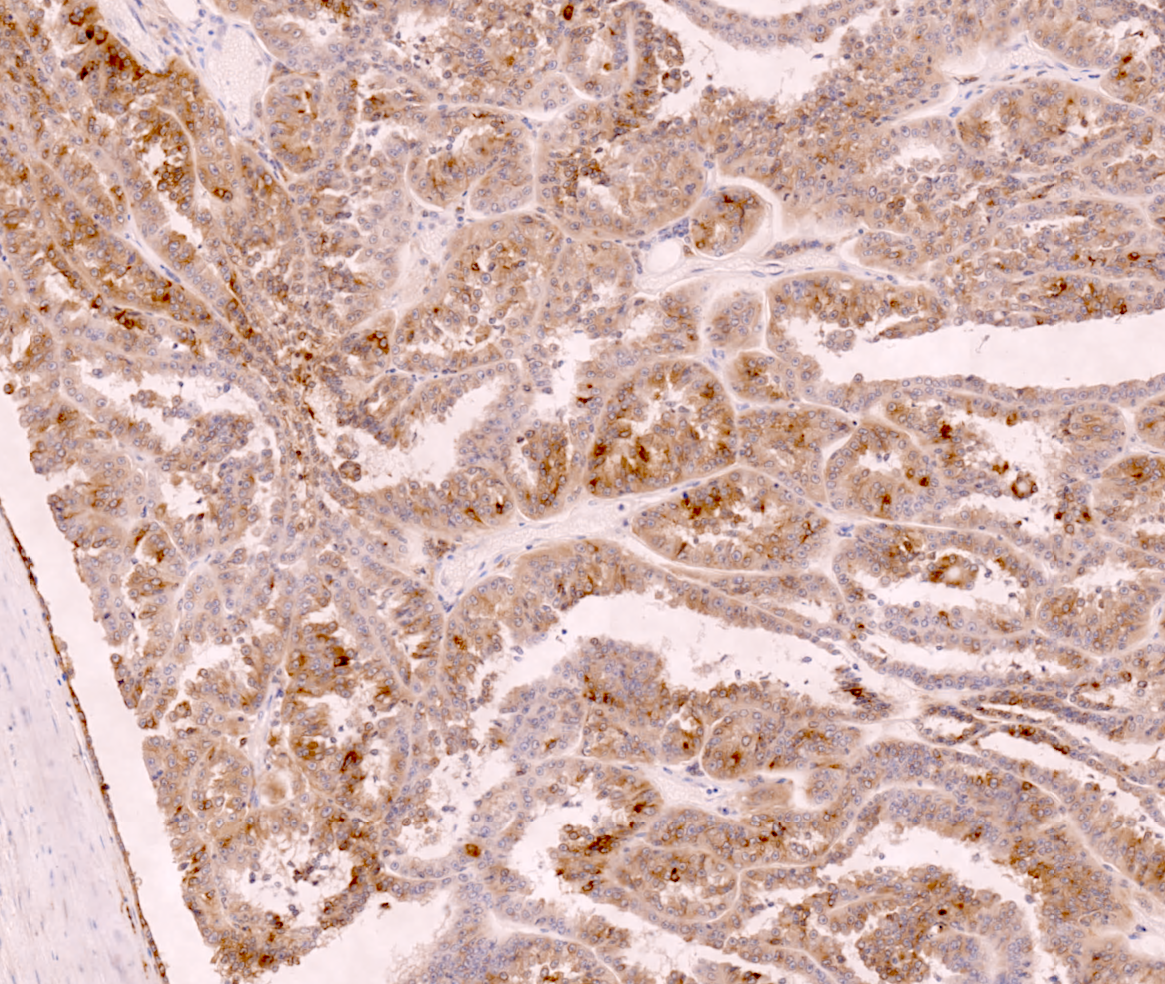

Supplement: Supplementary file 16 — Source Data for Figure 2 [file EMMM-15-e16877-s017.zip › Source_Data_Figure2/2A/TIF images for Figure 2A/BHD Patient 6 GPNMB.tif]

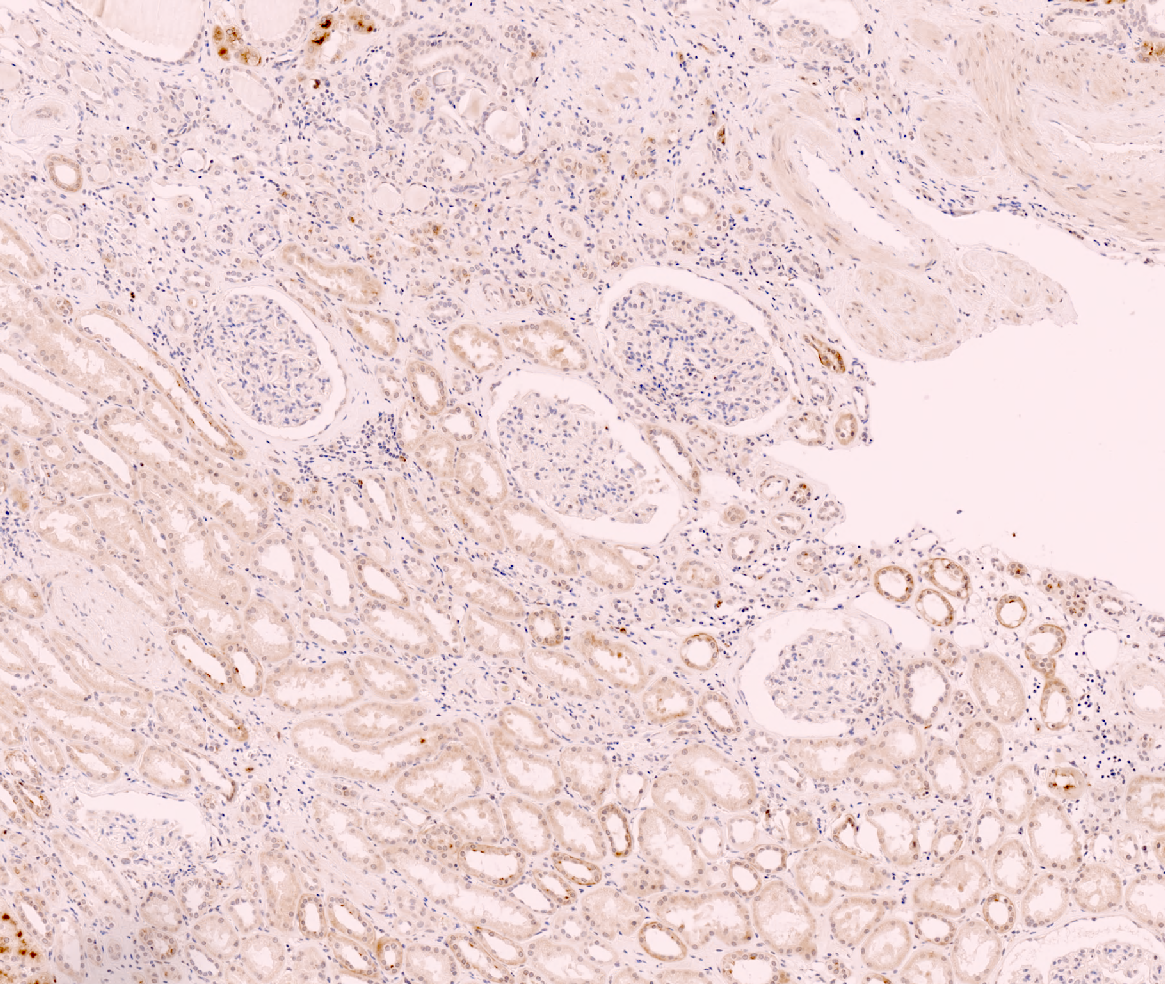

Supplement: Supplementary file 16 — Source Data for Figure 2 [file EMMM-15-e16877-s017.zip › Source_Data_Figure2/2A/TIF images for Figure 2A/BHD Patient 3 Normal NPC1.tif]

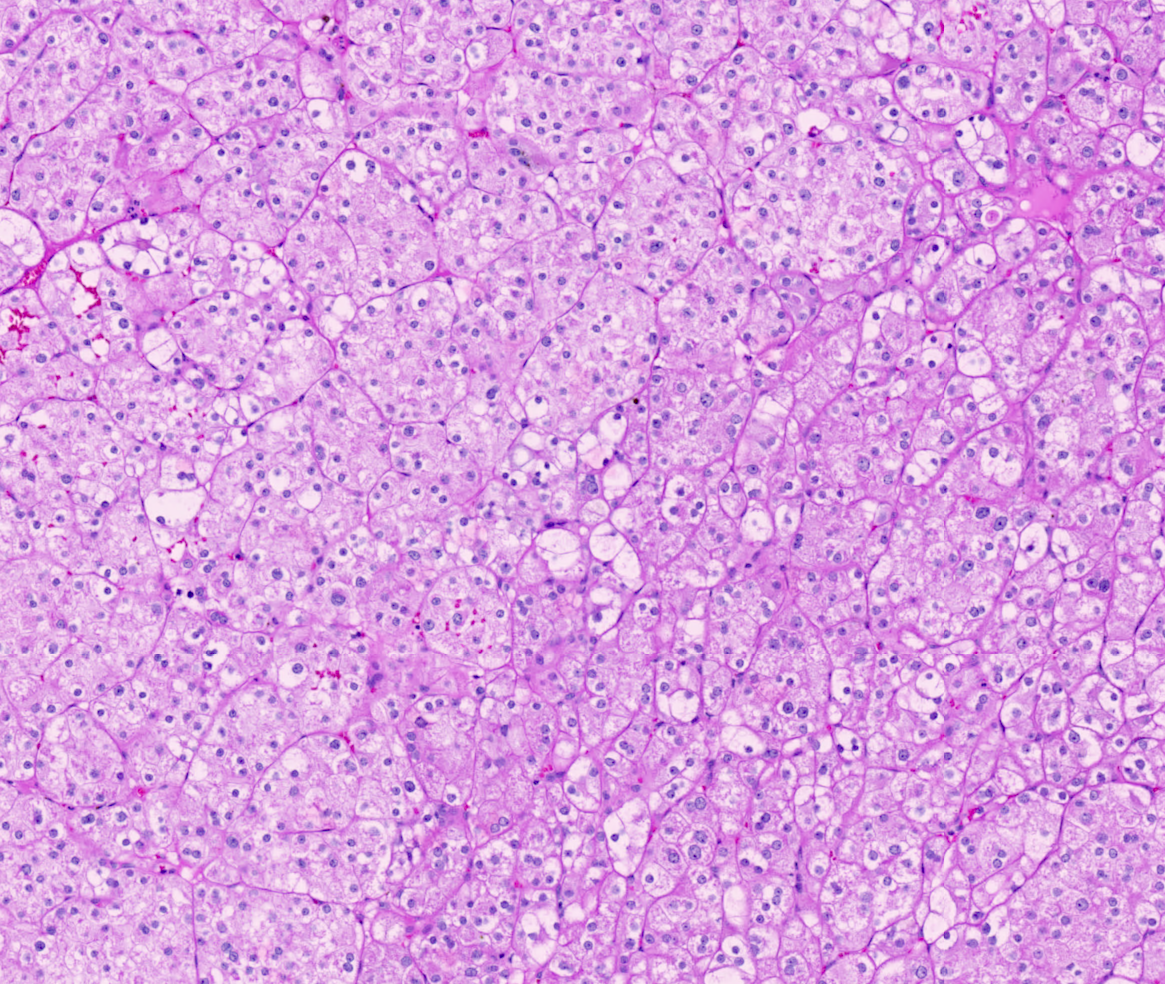

Supplement: Supplementary file 16 — Source Data for Figure 2 [file EMMM-15-e16877-s017.zip › Source_Data_Figure2/2A/TIF images for Figure 2A/BHD Patient 1 H&E.tif]

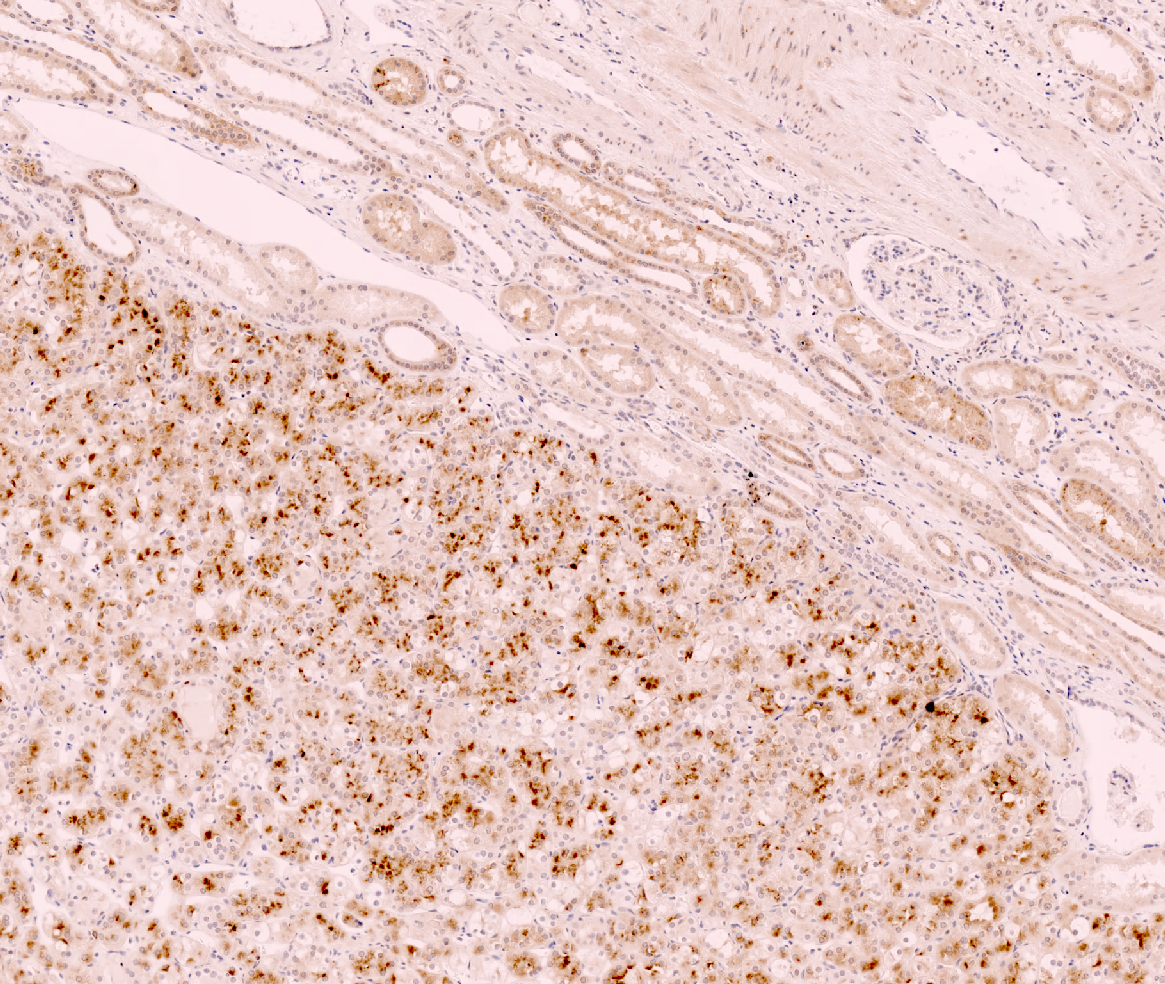

Supplement: Supplementary file 16 — Source Data for Figure 2 [file EMMM-15-e16877-s017.zip › Source_Data_Figure2/2A/TIF images for Figure 2A/BHD Patient 3 Tumor NPC1.tif]
