# Supplementary material for: TFEB and TFE3 drive kidney cystogenesis and tumorigenesis
Source: EMBO Mol Med. 2023 Mar 29;15(5):e16877. doi: 10.15252/emmm.202216877 (PMC10165358; doi:10.15252/emmm.202216877)
Supplement: Supplementary file 18 [file EMMM-15-e16877-s006.pdf]

EMBO Molecular Medicine

TFEB AND TFE3 DRIVE KIDNEY CYSTOGENESIS AND TUMORIGENESIS

Chiara Di Malta, Angela Zampelli, Letizia Granieri, Claudia Vilardo, Rossella De Cegli, Laura Cinque, Edoardo Nusco, Salvatore Pece, Daniela Tosoni, Francesca Sanguedolce, Nicolina Sorrentino, Maria Merino, Deborah Nielsen, Ramaprasad Srinivasan, Mark Ball, Christopher Ricketts, Cathy Vocke, Martin Lang, Baktiar Karim, Luisa Lanfrancone, Laura S. Schmidt, Marston W Linehan, and Andrea Ballabio
DOI: 10.15252/emmm.202216877

Corresponding author(s): Andrea Ballabio (ballabio@tigem.it) , Marston W Linehan (linehanm@mail.nih.gov), Chiara Di Malta (dimalta@tigem.it)

Review Timeline:

Submission Date:	13th Sep 22
Editorial Decision:	5th Oct 22
Revision Received:	27th Jan 23
Editorial Decision:	15th Feb 23
Revision Received:	27th Feb 23
Accepted:	28th Feb 23

Editor: Jingyi Hou

Transaction Report:

(Note: With the exception of the correction of typographical or spelling errors that could be a source of ambiguity, letters and reports are not edited. Depending on transfer agreements, referee reports obtained elsewhere may or may not be included in this compilation. Referee reports are anonymous unless the Referee chooses to sign their reports.)

6th Oct 2022

Dear Dr. Ballabio,

Thank you again for submitting your work to EMBO Molecular Medicine. We have now heard back from the three referees who agreed to evaluate your manuscript. As you will see from the reports below, the referees are overall supportive and think the study is interesting. Still, they raise a series of concerns, which we would ask you to address in a revision of the manuscript.

I think the referees' recommendations are relatively straightforward, and there is no need to reiterate all the points listed below. All issues raised by the referees need to be satisfactorily addressed. Please feel free to contact me in case you would like to discuss in further detail any of the issues raised by the referees.

We would welcome the submission of a revised version within three months for further consideration. Please note that EMBO Molecular Medicine strongly supports a single round of revision. As acceptance or rejection of the manuscript will depend on another round of review, your responses should be as complete as possible.

EMBO Molecular Medicine has a "scooping protection" policy, whereby similar findings that are published by others during review or revision are not a criterion for rejection. Should you decide to submit a revised version, I do ask that you get in touch after three months if you have not completed it to update us on the status.

We are aware that many laboratories cannot function at full efficiency during the current COVID-19/SARS-CoV-2 pandemic and have therefore extended our "scooping protection policy" to cover the period required for a full revision to address the experimental issues. Please let me know should you need additional time, and also if you see a paper with related content published elsewhere.

Please read below for important editorial formatting and consult our author's guidelines for proper formatting of your revised article for EMBO Molecular Medicine.

I look forward to receiving your revised manuscript.

Use this link to login to the manuscript system and submit your revision: https://embomolmed.msubmit.net/cgi-bin/main.plex

Kind regards,
Jingyi

Jingyi Hou
Editor
EMBO Molecular Medicine

*****

When submitting your revised manuscript, please carefully review the instructions that follow below. We perform an initial quality control of all revised manuscripts before re-review; failure to include requested items will delay the evaluation of your revision.

We require:

1) A .docx formatted version of the manuscript text (including legends for main figures, EV figures and tables). Please make sure that the changes are highlighted to be clearly visible.

2) Individual production quality figure files as .eps, .tif, .jpg (one file per figure). For guidance, download the 'Figure Guide PDF': (https://www.embopress.org/page/journal/17574684/authorguide#figureformat).

3) A .docx formatted letter INCLUDING the reviewers' reports and your detailed point-by-point responses to their comments. As part of the EMBO Press transparent editorial process, the point-by-point response is part of the Review Process File (RPF), which will be published alongside your paper.

4) A complete author checklist, which you can download from our author guidelines (https://www.embopress.org/page/journal/17574684/authorguide#submissionofrevisions). Please insert information in the

checklist that is also reflected in the manuscript. The completed author checklist will also be part of the RPF.

5) Please note that all corresponding authors are required to supply an ORCID ID for their name upon submission of a revised manuscript.

6) It is mandatory to include a 'Data Availability' section after the Materials and Methods. Before submitting your revision, primary datasets produced in this study need to be deposited in an appropriate public database, and the accession numbers and database listed under 'Data Availability'. Please remember to provide a reviewer password if the datasets are not yet public (see https://www.embopress.org/page/journal/17574684/authorguide#dataavailability).

In case you have no data that requires deposition in a public database, please state so in this section. Note that the Data Availability Section is restricted to new primary data that are part of this study.

7) For data quantification: please specify the name of the statistical test used to generate error bars and P values, the number (n) of independent experiments (specify technical or biological replicates) underlying each data point and the test used to calculate p-values in each figure legend. The figure legends should contain a basic description of n, P and the test applied. Graphs must include a description of the bars and the error bars (s.d., s.e.m.). See also 'Figure Legend' guidelines: https://www.embopress.org/page/journal/17574684/authorguide#figureformat

8) At EMBO Press we ask authors to provide source data for the main and expanded view figures. Our source data coordinator will contact you to discuss which figure panels we would need source data for and will also provide you with helpful tips on how to upload and organize the files.

9) Our journal encourages inclusion of *data citations in the reference list* to directly cite datasets that were re-used and obtained from public databases. Data citations in the article text are distinct from normal bibliographical citations and should directly link to the database records from which the data can be accessed. In the main text, data citations are formatted as follows: "Data ref: Smith et al, 2001" or "Data ref: NCBI Sequence Read Archive PRJNA342805, 2017". In the Reference list, data citations must be labeled with "[DATASET]". A data reference must provide the database name, accession number/identifiers and a resolvable link to the landing page from which the data can be accessed at the end of the reference. Further instructions are available at .

10) We replaced Supplementary Information with Expanded View (EV) Figures and Tables that are collapsible/expandable online. A maximum of 5 EV Figures can be typeset. EV Figures should be cited as 'Figure EV1, Figure EV2" etc... in the text and their respective legends should be included in the main text after the legends of regular figures.

- For the figures that you do NOT wish to display as Expanded View figures, they should be bundled together with their legends in a single PDF file called *Appendix*, which should start with a short Table of Content. Appendix figures should be referred to in the main text as: "Appendix Figure S1, Appendix Figure S2" etc.

- Additional Tables/Datasets should be labeled and referred to as Table EV1, Dataset EV1, etc. Legends have to be provided in a separate tab in case of .xls files. Alternatively, the legend can be supplied as a separate text file (README) and zipped together with the Table/Dataset file.

See detailed instructions here:

.

11) The paper explained: EMBO Molecular Medicine articles are accompanied by a summary of the articles to emphasize the major findings in the paper and their medical implications for the non-specialist reader. Please provide a draft summary of your article highlighting

- the medical issue you are addressing,

- the results obtained and

- their clinical impact.

This may be edited to ensure that readers understand the significance and context of the research. Please refer to any of our published articles for an example.

12) For more information: There is space at the end of each article to list relevant web links for further consultation by our readers. Could you identify some relevant ones and provide such information as well? Some examples are patient associations, relevant databases, OMIM/proteins/genes links, author's websites, etc...

13) Author contributions: You will be asked to provide CRediT (Contributor Role Taxonomy) terms in the submission system. These replace a narrative author contribution section in the manuscript.

14) A Conflict of Interest statement should be provided in the main text.

15) Every published paper now includes a 'Synopsis' to further enhance discoverability. Synopses are displayed on the journal webpage and are freely accessible to all readers. They include a short stand first (maximum of 300 characters, including space) as well as 2-5 one-sentences bullet points that summarizes the paper. Please write the bullet points to summarize the key NEW findings. They should be designed to be complementary to the abstract - i.e. not repeat the same text. We encourage inclusion of key acronyms and quantitative information (maximum of 30 words / bullet point). Please use the passive voice. Please attach these in a separate file or send them by email, we will incorporate them accordingly.

Please also suggest a striking image or visual abstract to illustrate your article as a PNG file 550 px wide x 300-600 px high.

EMBO Molecular Medicine has a "scooping protection" policy, whereby similar findings that are published by others during review or revision are not a criterion for rejection. Should you decide to submit a revised version, I do ask that you get in touch after three months if you have not completed it, to update us on the status.

Please note: When submitting your revision you will be prompted to enter your funding and payment information. This will allow Wiley to send you a quote for the article processing charge (APC) in case of acceptance. This quote takes into account any reduction or fee waivers that you may be eligible for. Authors do not need to pay any fees before their manuscript is accepted and transferred to the publisher.

EMBO Press participates in many Publish and Read agreements that allow authors to publish Open Access with reduced/no publication charges. Check your eligibility: https://authorservices.wiley.com/author-resources/Journal-Authors/open-access/affiliation-policies-payments/index.html

***** Reviewer's comments *****

Referee #1 (Remarks for Author):

In the present study, Malta et al. show that Tfeb and Tfe3 transcription factors contribute, in a differential and cooperative manner, to kidney cystogenesis in Folliculin (FLCN)/Tfeb/Tfe3 double and triple knockout mice. Of note, the analysis of Birt-Hogg-Dubé (BHD) patient-derived tumor samples revealed augmented activation of the TFEB/TFE3-mediated transcriptional program. In addition, silencing of either of the two genes rescued tumorigenesis in human BHD renal tumor cell line-derived xenografts. The authors conclude that TFEB and TFE3 are critical drivers of renal tumorigenesis and suggest novel therapeutic strategies based on inhibiting these transcription factors.
The study by Malta et al. is engaging, well-written, and provides relevant insights into the molecular pathogenesis of renal carcinogenesis. The appropriate methodologies were used to reach the proposed goals. The data are robust and support the conclusions drawn.
Major issue:
The authors revealed a series of downstream targets of Tfeb and Tfe3. Due to the lack of specific inhibitors for Tfeb and Tfe3, the authors should challenge some identified targets via gene silencing and/or soluble inhibitors, at least in vitro. These data would undoubtedly increase the relevance of the present study.

Referee #2 (Comments on Novelty/Model System for Author):

The use of gene knockout mice is a powerful means to analyze gene function. In this study, through the use of these mice, combined with tissue and cell level analysis, the author carried out their research. These methodologies are appropriate for the study。

Referee #2 (Remarks for Author):

In this short report, based on previous studies, the author investigated the role of MiT-TFE family genes in the development of renal cysts and tumors by focusing on TFE3 and using gene KO mice and human tumor samples. Although the function of TFE3 is considered to overlap with that of TFEB, their results explain the different phenotypes of TFEB and TFE3 KO mice and indicate that both TFEB and TFE3 contribute to renal tumorigenesis. Overall, this is an interesting report. The experiments were well-conducted and the manuscript was well-organized. In addition to the role of TFE3, gene expression profiles based on mice and tumor tissues also provide important clues for related research. I just have a few comments on the core issues of the study.

1. The authors believe that the different contributions of TFEB and TFE3 to renal pathology observed in Flcn KO mice are due to the different expression levels of these transcription factors in specific renal cell populations. Because this is the key point of the story, in addition to TFEB expression in Flcn KO mice, TFEB expression in Flcn/Tfe3 DKO mice and TFE3 expression in Tfeb/Flcn DKO mice should be checked.

2. The conclusion that TFE3 participates to the renal pathology in Flcn KO mice is only based on the longer life span of Flcn/Tfe3 DKO; Tfeb-HET mice. Analyzing the homodimer of TFEB and TFE3 in mice will be very helpful, because it was previously shown that the heterodimeric interaction is not essential for Mitf-Tfe function (PNAS 2002). Similar experiments can be conducted in UOK257/shTFEB cells and UOK257/shTFE3 cells respectively.

3. In supplementary Fig. 2, what's the control UOK257-2 cell? The localization of TFEB and TFE3 in untreated cells should be shown as the constitutive nuclear localization in UOK257 cells.

Referee #3 (Comments on Novelty/Model System for Author):

Highly rigorous genetically engineered mouse models and cell line/tumor data. No technical concerns.

Referee #3 (Remarks for Author):

Review for Experimental and Molecular Medicine of:

TFEB AND TFE3 DRIVE KIDNEY CYSTOGENESIS AND TUMORIGENESIS
Chiara Di Malta1,2*, Angela Zampelli1, Letizia Granieri3, Claudia Vilardo1, Rossella De Cegli1, Edoardo Nusco1, Salvatore Pece3, Daniela Tosoni3, Francesca Sanguedolce4, Nicolina Cristina Sorrentino1,5, Maria J. Merino6, Mark W. Ball7, Christopher J. Ricketts7, Cathy D.Vocke7, Martin Lang7, Baktiar Karim8, Luisa Lanfrancone3, Laura S Schmidt 7,9, W Marston Linehan7*and Andrea Ballabio10*.

Summary:

This study examines the importance of the autophagy regulators TFEB and TFE3 to cystogenesis/tumorigenesis in the setting of FLCN loss/BHD Syndrome. The authors utilize double and triple knockout mice and cell lines to explore relative contributions of each MiT-TFE family member to disease pathology, finding that KO of TFEB in FLCN KO mice restores WT kidney function/morphology while TFE3 has no significant effect. The authors attribute this to significantly higher TFEB expression vs TFE3 in relevant tissues and go on to show the TFE3 KO in TFEB heterozygotes did give some protection against renal cyst lethality.

The authors also stained several human BHD tumor biopsies and demonstrated significant TFEB/TFE3 nuclear localization/activation as predicted. RNA seq data from BHD tumors also showed significant upregulation of known TFEB targets, particularly lysosome-related genes. A BHD tumor-derived cell line also showed hyperactivation of TFEB and TFE3, with shRNA-mediated KD of either being sufficient to prevent tumor growth in a xenograft mouse model. Comparison of gene sets upregulated in BHD tumors but downregulated in TFEB/TFE3 KO tumors showed several overlapping genes related to autophagy and lysosome function.

Overall this is a very focused but interesting and well-designed study elucidating relative contributions of MiT-TFE family members to the patholobiology of this rare disease.

Major Points:

1. BHD is a relatively rare disease and your assembled cohort of patients/tumors seems like a very valuable resource. Curious if TFEB or TFE3 expression levels in patients correlate with overall survival, cyst burden, presence of extra-renal manifestations, or any other clinical parameters in your patients. Or could these associations be explored in any publicly-available data sets such as TCGA? Such an observation could potentially help to provide further human validation.

2. The observed effects of TFEB/TFE3 manipulation using both transgenic mice and xenografts discuss the importance of TFEB/TFE3 to lysosome biogenesis, which is well-supported by several transcriptomic experiments showing differential expression of multiple lysosome-related genes. It might be good to do a few confirmatory experiments using commercially-available dyes to measure both changes in total cellular lysosome mass and activity in model cell lines pre- and post-KD either by IF or flow cytometry. By the same token, it might also be interesting to compare staining for lysosome markers in both FLCN KO and FLCN double or triple KO mice or even in patient tumor sections relative to control kidney tissue

3. TFEB in particular is also known to activate mitochondrial biogenesis and oxidative phosphorylation, both of which can be important modulators of tumor growth. Staining with mitotracker or other commercial mitochondrial activity/mass dyes might help

rule out significant contribution from changes in energy balance to tumor growth. Metabolic measurements such as those performed by Agilent's Seahorse series could fulfill the same function.

4. As also touched on above, interpretation of the importance of TFEB and TFE3 relate to control of both of these TFs over both lysosome biogenesis and autophagy, but both of these genes have other targets related to a broad range of metabolic functions that could be important for tumor engraftment and growth in a xenograft model. You might consider sh- or CRISPR-mediated silencing of downstream, autophagy-specific genes such as ATG family members to see if these phenocopy TFEB/TFE3 KO grafts to see how important the autophagy arm of MiT-TFE family members' functions really are.

5. Translocation renal cell carcinomas are a rare and aggressive subtype, most commonly seen in pediatric patients, and are characterized by translocations of either TFEB, TFE3, or MITF. Given that MITF has also been suggested to exert control over autophagy in some contexts, might be interesting to see if MITF KD in U0K257 cells also downregulates any of the same lysosome or autophagy genes by RNA seq. If you see a similar pattern, it may broaden the importance of these findings out to additional subtypes of kidney cancer, though this may be beyond the scope of the current BHD-focused study.

Minor Points:

None

******* Reviewer's comments *******

Referee #1 (Remarks for Author):

In the present study, Malta et al. show that Tfeb and Tfe3 transcription factors contribute, in a differential and cooperative manner, to kidney cystogenesis in Folliculin (FLCN)/Tfeb/Tfe3 double and triple knockout mice. Of note, the analysis of Birt-Hogg-Dubé (BHD) patient-derived tumor samples revealed augmented activation of the TFEB/TFE3-mediated transcriptional program. In addition, silencing of either of the two genes rescued tumorigenesis in human BHD renal tumor cell line-derived xenografts. The authors conclude that TFEB and TFE3 are critical drivers of renal tumorigenesis and suggest novel therapeutic strategies based on inhibiting these transcription factors.
The study by Malta et al. is engaging, well-written, and provides relevant insights into the molecular pathogenesis of renal carcinogenesis. The appropriate methodologies were used to reach the proposed goals. The data are robust and support the conclusions drawn.
Major issue:
The authors revealed a series of downstream targets of Tfeb and Tfe3. Due to the lack of specific inhibitors for Tfeb and Tfe3, the authors should challenge some identified targets via gene silencing and/or soluble inhibitors, at least in vitro. These data would undoubtedly increase the relevance of the present study.

-We thank the reviewer for the positive evaluation of our manuscript and for his/her valuable suggestions to improve it. As suggested by this reviewer, we used siRNA approach to downregulate the 28 TFEB/TFE3 target genes selected by combining the list of TFEB/TFE3 target genes significantly upregulated in BHD tumor samples with the list of targets downregulated in UOK257 cells silenced for TFEB or TFE3 (Supplementary table 2). As readout, we monitored cell proliferation through MTT assay. We found that downregulation of several target genes significantly reduced proliferation of UOK257 cells relative to control cells silenced for scramble (new Supplementary Fig 6), in line with the idea that TFEB/TFE3 promote tumorigenesis through the regulation of several genes. These data may further restrict the list of target genes responsible for cell proliferation. Further analyses, especially in vivo, are needed to really understand whether specific TFEB/TFE3 targets are primarily responsible for tumor formation and growth. However, these studies are beyond the scope of this manuscript.

Referee #2 (Comments on Novelty/Model System for Author):

The use of gene knockout mice is a powerful means to analyze gene function. In this study, through the use of these mice, combined with tissue and cell level analysis, the author carried out their research. These methodologies are appropriate for the study。

Referee #2 (Remarks for Author):

In this short report, based on previous studies, the author investigated the role of MiT-TFE family genes in the development of renal cysts and tumors by focusing on TFE3 and using gene KO mice and human tumor samples. Although the function of TFE3 is considered to overlap with that of TFEB, their results explain the different phenotypes of TFEB and TFE3 KO mice and indicate that both TFEB and TFE3 contribute to renal tumorigenesis. Overall, this is an interesting report. The experiments were well-conducted and the manuscript was well-organized. In addition to the role of TFE3, gene expression profiles based on mice and tumor tissues also provide important clues for related research. I just have a few comments on the core issues of the study.

-We would like to thank this reviewer for pointing out the essence of our paper and for identifying all of the most important aspects.

1. The authors believe that the different contributions of TFEB and TFE3 to renal pathology observed in Flcn KO mice are due to the different expression levels of these transcription factors in specific renal cell populations. Because this is the key point of the story, in addition to TFEB expression in Flcn KO mice, TFEB expression in Flcn/Tfe3 DKO mice and TFE3 expression in Tfeb/Flcn DKO mice should be checked.

-We agree with the reviewer that testing TFEB levels in the Flcn/Tfe3 DKO mice and TFE3 expression in Tfeb/Flcn DKO mice would represent a good control for our experiments. Unfortunately, these DKO mice are not currently available in our laboratory, and it would take long time to re-establish these murine lines. Our previous studies indicate that TFEB and TFE3 play a cooperative role by regulating very similar sets of genes. Indeed, TFE3 full KO mice present metabolic phenotypes that are very similar to TFEB liver-, muscle-, and macrophage-specific conditional KO mice, and these phenotypes are more severe in TFEB/TFE3 DKO mice (Pastore *et al*, 2016, 2017). Notably, both TFEB overexpression in Tfe3 KO mice and TFE3 overexpression in TFEB-liver specific KO mice rescued the obesity phenotype associated with the KOs of these transcription factors, indicating that TFEB can compensate, at least partially, for TFE3 deficiency and vice versa(Pastore *et al*, 2017). We believe that the results presented in this manuscript are in line with our previous observations and that the different levels of TFEB and TFE3 in the cadherin 16-expressing cells may be responsible for the diverse phenotypes of kidney-specific FLCN/TFEB DKO mice and FLCN/TFE3 DKO mice. However, this is not the condition observed in patients, where both TFEB and TFE3 are highly expressed and constitutively activated in BHD-kidney tumors (Fig 2a). In line with this, we also show that both the single depletion of TFEB and the single depletion of TFE3 are sufficient to abolish the growth of BHD-tumor cells (UOK257)-derived xenografts (Fig 3a-f), clearly demonstrating that both TFEB and TFE3 drive tumorigenesis in BHD syndrome. This is the major conclusion of our study.

2. The conclusion that TFE3 participates to the renal pathology in Flcn KO mice is only based on the longer life span of Flcn/Tfe3 DKO; Tfeb-HET mice. Analyzing the homodimer of TFEB and TFE3 in mice will be very helpful, because it was previously shown that the heterodimeric interaction is not essential for Mitf-Tfe function (PNAS 2002). Similar experiments can be conducted in UOK257/shTFEB cells and UOK257/shTFE3 cells respectively.

We would like to clarify that the main evidence regarding the involvement of TFE3 in BHD renal pathology derives from the xenograft experiments (Fig 3 a-f), in which the single silencing of TFE3 is sufficient to abolish tumor growth.
As the reviewer stated, previous studies indicate that heterodimeric interaction is not essential for MiT/TFE function(Steingrimsson *et al*, 2002). Therefore, no specific function has been attributed to MiT/TFE heterodimers vs homodimers and the different contribution of each transcription factor to specific developmental or pathological conditions is most likely due to different levels of each factor in a time and tissue-specific fashion. Moreover, we believe that testing the presence of homodimers vs heterodimers from renal tissues or cells upon depletion of TFEB or TFE3 would not be really informative, because it is expected that silencing one of the two genes would impair the formation of heterodimers.
Testing the pathological relevance of TFEB/TFE3 heterodimers vs homodimers would require knock-in experiments in which one gene, TFEB or TFE3, is knocked-in in the locus of the other one. By doing so, gene dosage would be preserved but heterodimer formation abolished, thus allowing to really understand whether specific function may be attributed to TFEB/TFE3 heterodimers. However, this biological question is beyond the scope of our manuscript.

3. In supplementary Fig. 2, what's the control UOK257-2 cell? The localization of TFEB and TFE3 in untreated cells should be shown as the constitutive nuclear localization in UOK257 cells.

-We are sorry for the lack of clarity of Supplementary Figure 2 (now Supplementary Figure 3) legend. As stated in the manuscript, the UOK257 cells were derived from an individual with BHD syndrome and carry biallelic FLCN loss-of-function mutations(Yang *et al*, 2008). The UOK257-2 cells were generated by transducing the UOK257 cells with a lentiviral vector expressing FLCN(Baba *et al*, 2006), thus representing the control cell line for UOK257 cells.
TFEB and TFE3 need to bind to active Rags to be phosphorylated by mTORC1 and hence retained in the cytosol(Napolitano *et al*, 2020). Since FLCN is the GAP responsible for promoting RagC/D activation upon amino acid stimulation, the best way to monitor whether the lack of FLCN affects TFEB/TFE3 localization is to follow this parameter upon amino acid stimulation.
In control UOK257-2 cells, both TFEB and TFE3 are nuclear upon amino acids starvation, when Rags are inactive, and cytosolic once re-stimulated with amino acids, when Rags are fully active. As control, treatment with the mTOR inhibitor torin-1 impairs TFEB/TFE3 phosphorylation, thus promoting their nuclear accumulation, also in presence of amino acids. In UOK257 cells, the lack of FLCN impairs RagC/D activation, thus resulting in constitutive nuclear localization of TFEB and TFE3, also in presence of amino acids. We hope that this explanation can dispel the reviewer's concerns about this figure.

Referee #3 (Comments on Novelty/Model System for Author):

Highly rigorous genetically engineered mouse models and cell line/tumor data. No technical concerns.

Referee #3 (Remarks for Author):

Review for Experimental and Molecular Medicine of:

TFEB AND TFE3 DRIVE KIDNEY CYSTOGENESIS AND TUMORIGENESIS
Chiara Di Malta1,2*, Angela Zampelli1, Letizia Granieri3, Claudia Vilardo1, Rossella De Cegli1, Edoardo Nusco1, Salvatore Pece3, Daniela Tosoni3, Francesca Sanguedolce4, Nicolina Cristina Sorrentino1,5, Maria J. Merino6, Mark W. Ball7, Christopher J. Ricketts7, Cathy D.Vocke7, Martin Lang7, Baktiar Karim8, Luisa Lanfrancone3, Laura S Schmidt 7,9, W Marston Linehan7*and Andrea Ballabio10*.

Summary:

This study examines the importance of the autophagy regulators TFEB and TFE3 to cystogenesis/tumorigenesis in the setting of FLCN loss/BHD Syndrome. The authors utilize double and triple knockout mice and cell lines to explore relative contributions of each MiT-TFE family member to disease pathology, finding that KO of TFEB in FLCN KO mice restores WT kidney function/morphology while TFE3 has no significant effect. The authors attribute this to significantly higher TFEB expression vs TFE3 in relevant tissues and go on to show the TFE3 KO in TFEB heterozygotes did give some protection against renal cyst lethality.

The authors also stained several human BHD tumor biopsies and demonstrated significant TFEB/TFE3 nuclear localization/activation as predicted. RNA seq data from BHD tumors also showed significant upregulation of known TFEB targets, particularly lysosome-related genes. A BHD tumor-derived cell line also showed hyperactivation of TFEB and TFE3, with shRNA-mediated KD of either being sufficient to prevent tumor growth in a xenograft mouse

model. Comparison of gene sets upregulated in BHD tumors but downregulated in TFEB/TFE3 KO tumors showed several overlapping genes related to autophagy and lysosome function.

Overall this is a very focused but interesting and well-designed study elucidating relative contributions of MiT-TFE family members to the patholobiology of this rare disease.

-We thank the reviewer for her/his positive comments on our manuscript.

Major Points:

1. BHD is a relatively rare disease and your assembled cohort of patients/tumors seems like a very valuable resource. Curious if TFEB or TFE3 expression levels in patients correlate with overall survival, cyst burden, presence of extra-renal manifestations, or any other clinical parameters in your patients. Or could these associations be explored in any publicly-available data sets such as TCGA? Such an observation could potentially help to provide further human validation.

-We thank the reviewer for the very thoughtful comment. We have looked in the TCGA and the CPTAC RCC cohorts and they do not contain patients with germline FLCN mutations. A small number of cases have been reported with somatic FLCN mutations in the TCGA kidney papillary cell carcinoma cohort 3 out of 276 tumors were mutated, but there are not enough samples to look for a correlation with survival. Most BHD tumors are either hybrid oncocytic (59%) or chromophobe (33%) and they tend to have a very constant, slow growth rate (1 mm/yr). We do not currently have data to evaluate a correlation between the influences of TFE3 or TFEB on survival. However, we have had a number of BHD patients whose tumors have a non-canonical pathologic phenotypes that are nearly identical to those seen in patients carrying chromosomal translocations involving TFE3 or TFEB, associated with TFE3 renal cell carcinoma (papillary clear pathologic phenotype) or TFEB renal cell carcinoma (eosinophilic clear cell RCC), indicating the clear driver characteristics of TFE3 and TFEB in this entity. In order to respond to the reviewers's thoughtful comments about a potential clinical phenotypic manifestation in renal tumors, (new Fig 2a in the paper) as well as adding the following text to the discussion of the manuscript: "To investigate the activation state of TFEB and TFE3 in BHD patient-derived tissue samples, we performed histological examination of their cellular localization in renal tumors removed surgically at the National Cancer Institute from seven unrelated BHD patients (Figure 2a and Supplementary Table 1). While BHD-associated kidney tumors are characterized by a variety of histological subtypes, most frequently hybrid oncocytic tumor (50%: Patients 1&2) and chromophobe (35%:Patient 3) renal tumors(Schmidt & Linehan, 2015), we have also encountered BHD patients with more aggressive papillary/cystic histologic and/or clinical phenotypes (consistent with TFE3 fusion/translocation RCC) (Patients 4, 5 and 6) and eosinophilic/clear histology RCC reminiscent of TFE3/B translocation fusion RCC (Patient 7, UOB 257 cell line). The patients with pathologic phenotypes remiscent of TFE3/TFEB RCC tended to have a more aggressive clinical phenotype. Patients 6 and 7 developed metastatic disease and patient 5 became dialysis-dependent after surgery on both kidneys. We found increased nuclear staining of both TFEB and TFE3 in BHD hybrid and chromophobe RCC relative to control kidney tissues, as well as in the TFE3/TFEB-like BHD tumors with tubulo papillary/eosinophilic clear RCC, which correlated with increased staining of the Glycoprotein non-metastatic melanoma protein B (GPNMB), and the lysosomal marker NPC intracellular cholesterol transporter 1 (NPC1), highly responsive TFEB/TFE3 transcriptional targets (Fig 2a)."

2. The observed effects of TFEB/TFE3 manipulation using both transgenic mice and xenografts discuss the importance of TFEB/TFE3 to lysosome biogenesis, which is well-supported by several transcriptomic experiments showing differential expression of multiple

lysosome-related genes. It might be good to do a few confirmatory experiments using commercially-available dyes to measure both changes in total cellular lysosome mass and activity in model cell lines pre- and post-KD either by IF or flow cytometry. By the same token, it might also be interesting to compare staining for lysosome markers in both FLCN KO and FLCN double or triple KO mice or even in patient tumor sections relative to control kidney tissue.

-We thank the reviewer for this important suggestion. As shown in new Fig 2a, we stained a group of BHD tumors with different histological presentations with the NPC-1 lysosomal marker and found consistent staining with NPC-1 in the areas of the tumors which also stain for nuclear TFE3/TFEB and GPNMB. Moreover, we performed lysotracker and DQ-BSA staining to monitor lysosomal quantity and degradative ability, respectively, in UOK257 cells upon silencing of TFEB or TFE3 relative to control UOK257 transfected with scramble siRNA. In line with our hypothesis, the downregulation of TFEB or the one of TFE3 significantly reduced both lysosomal parameters (new Supplementary Fig 5). Finally, we stained kidney tissue from control or Flcn KO mice with LAMP1 antibody and found significant upregulation of this lysosomal marker (new Supplementary Fig 1b). Altogether, the results of these analyses are in line with our transcriptomic data, suggesting that excessive activation of lysosomal functions represent an important contributor to kidney pathology in BHD syndrome.

3. TFEB in particular is also known to activate mitochondrial biogenesis and oxidative phosphorylation, both of which can be important modulators of tumor growth. Staining with mitotracker or other commercial mitochondrial activity/mass dyes might help rule out significant contribution from changes in energy balance to tumor growth. Metabolic measurements such as those performed by Agilent's Seahorse series could fulfill the same function.

-We thank the reviewer for this excellent suggestion. There is a considerable amount of published data showing that BHD tumors and the UOK257 cell line have increased expression of PGC1a, a key regulator of mitochondrial biogenesis and an established TFEB/TFE3 target gene (Hasumi *et al*, 2012, 2014; Settembre *et al*, 2013). In addition, TFEB also promotes the expression of several genes involved in mitochondrial biogenesis, fatty acid oxidation, and oxidative phosphorylation(Mansueto *et al*, 2017). However, when we compared the list of TFEB/TFE3 target genes significantly upregulated in BHD tumor samples with the list of targets downregulated in UOK257 cells silenced for TFEB or TFE3, we did not find significant alteration of the mitochondrial gene category (Fig 3g, h, Supplementary Table 2, Supplementary Dataset 7). We also performed western blot analysis of UOK257 silenced for TFEB or TFE3 relative to control cells silenced for scramble and monitored the levels of several mitochondrial proteins to understand whether downregulation of these transcription factors could impact mitochondrial pathway in this disease cellular model. However, in line with our transcriptomic data, the silencing of TFEB or TFE3 was not associated with detectable reduction of the monitored mitochondrial proteins in UOK257 cells (Rebuttal Fig 1). Therefore, our result do not support the hypothesis that increased mitochondrial activity is the main mechanism by which MiT-TFE factors promote kidney tumorigenesis, however, this possibility cannot be completely ruled based on these correlative data.

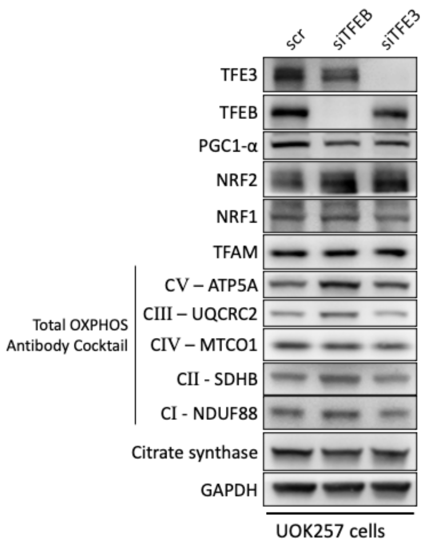

Rebuttal Fig. 1. **Silencing of TFEB or TFE3 does not affect mitochondria protein content.** Representative western blot analysis of the indicated proteins in UOK257 cells transfected with scramble siRNA (scr) or with siRNA targeting TFEB (siTFEB) or TFE3 (siTFE3).
Replicated three times.

4. As also touched on above, interpretation of the importance of TFEB and TFE3 relate to control of both of these TFs over both lysosome biogenesis and autophagy, but both of these genes have other targets related to a broad range of metabolic functions that could be important for tumor engraftment and growth in a xenograft model. You might consider sh- or CRISPR-mediated silencing of downstream, autophagy-specific genes such as ATG family members to see if these phenocopy TFEB/TFE3 KO grafts to see how important the autophagy arm of MiT-TFE family members' functions really are.

-We agree with this reviewer about the importance of TFEB/TFE3-mediated transcriptional regulation of autophagy pathway in different physiological and pathological conditions, and it would certainly be interesting to test its contribution to the growth of BHD associated kidney tumors. However, our selected list of candidate TFEB/TFE3 target genes (Supplementary table 2) do not include ATG proteins, thus suggesting us that increased transcriptional activation of autophagy pathway in BHD kidney tumors is not a major factor responsible of tumorigenesis. Therefore, we believe that the suggested experiment is beyond the scope of our study.

5. Translocation renal cell carcinomas are a rare and aggressive subtype, most commonly seen in pediatric patients, and are characterized by translocations of either TFEB, TFE3, or MITF. Given that MITF has also been suggested to exert control over autophagy in some contexts, might be interesting to see if MITF KD in U0K257 cells also downregulates any of the same lysosome or autophagy genes by RNA seq. If you see a similar pattern, it may broaden the importance of these findings out to additional subtypes of kidney cancer, though this may be beyond the scope of the current BHD-focused study.

-We thank this reviewer for suggesting us this experiment. With the exception of the melanocyte-specific MITF isoform, which is not subjected to mTORC1 regulation but shows constitutive nuclear localization(Roczniak-Ferguson *et al*, 2012), the cellular localization of the other MITF isoforms should also be sensitive to FLCN activity. Therefore, it is reasonable to think that increased activation of MITF also contributes to tumorigenesis in BHD. As suggested by this reviewer, we performed MITF siRNA in UOK257 cells and then evaluated the transcriptional profile of silenced cells relative to control cells transfected with scramble siRNA through RNA-seq analysis. We were quite surprised to find that downregulation of MITF in UOK257 cells is not associated with significant downregulation of lysosomal pathway, differently from what observed upon silencing of TFEB or TFE3 (Rebuttal Fig 2a,

Supplementary Fig 4 C). In detail, as shown in Rebuttal Fig 2b, the expression level of the 16 lysosomal/autophagy genes significantly downregulated upon silencing of TFEB or TFE3 in UOK257 cells (Supplementary Dataset 6) was largely unaffected upon silencing of MITF, being SQSTM1 (highlighted in green) the only transcript to be downregulated, whereas RAB7 (in red) was even upregulated. These results suggest that MITF does not have a major role of kidney tumorigenesis in BHD syndrome. Since MiT/TFE factors are known to regulate largely overlapping sets of target genes, their relative contribution is likely to be due to their different expression levels in the tested tumor cells. We believe that future in vivo studies are needed to rule out the involvement of MITF in BHD syndrome, therefore, we decided not to include these data in the manuscript.

a

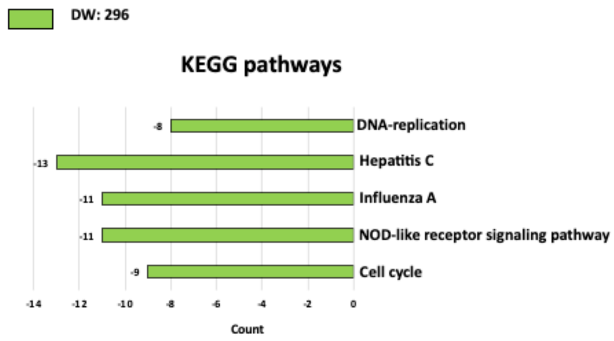

b

Symbol	log2FoldChange	padj
SQSTM1	-0,44783387	0,001456176
RAB7A	0,341976827	0,049506057
GNS	0,300080051	0,117354954
RAB1A	0,332124834	0,25044218
TPP1	0,240029712	0,394515721
GAA	0,357519727	0,413946632
CD164	0,261874099	0,553506189
PPT1	0,132111647	0,881133187
ATP6V1H	0,076503453	0,9647425
ATP6V0B	-0,04071459	0,9647425
PIK3CD	-0,116856917	0,9647425
IGF2R	0,137771702	0,9647425
UVRAG	0,116077669	0,9647425
ACP5	0,039687987	0,966457482
MCOLN1	0,014618356	0,995020286
RRAGC	0,000185646	0,999577677

Rebuttal Fig. 2. **Silencing of MITF in UOK257 is not associated with a significant downregulation of lyso-autophagy pathway.** (a) Kegg pathways associated with genes significantly downregulated upon silencing of MITF in UOK257 cells. (b) Table relative to the expression levels of the 16 lysosomal or autophagy genes significantly downregulated upon silencing of TFEB or TFE3 (Supplementary Dataset 5).

Minor Points:

None

References

- Baba M, Hong S-B, Sharma N, Warren MB, Nickerson ML, Iwamatsu A, Esposito D, Gillette WK, Hopkins RF, Hartley JL, *et al* (2006) Folliculin encoded by the BHD gene interacts with a binding protein, FNIP1, and AMPK, and is involved in AMPK and mTOR signaling. *Proc Natl Acad Sci USA* 103: 15552–15557
- Hasumi H, Baba M, Hasumi Y, Huang Y, Oh H, Hughes RM, Klein ME, Takikita S, Nagashima K, Schmidt LS, *et al* (2012) Regulation of mitochondrial oxidative metabolism by tumor suppressor FLCN. *J Natl Cancer Inst* 104: 1750–1764
- Hasumi Y, Baba M, Hasumi H, Huang Y, Lang M, Reindorf R, Oh H, Sciarretta S, Nagashima K, Haines DC, *et al* (2014) Folliculin (Flcn) inactivation leads to murine cardiac hypertrophy through mTORC1 deregulation. *Hum Mol Genet* 23: 5706–5719
- Mansueto G, Armani A, Viscomi C, D'Orsi L, De Cegli R, Polishchuk EV, Lamperti C, Di Meo I, Romanello V, Marchet S, *et al* (2017) Transcription Factor EB Controls Metabolic Flexibility during Exercise. *Cell Metab* 25: 182–196

- Napolitano G, Di Malta C, Esposito A, de Araujo MEG, Pece S, Bertalot G, Matarese M, Benedetti V, Zampelli A, Stasyk T, *et al* (2020) A substrate-specific mTORC1 pathway underlies Birt-Hogg-Dubé syndrome. *Nature*
- Pastore N, Brady OA, Diab HI, Martina JA, Sun L, Huynh T, Lim J-A, Zare H, Raben N, Ballabio A, *et al* (2016) TFEB and TFE3 cooperate in the regulation of the innate immune response in activated macrophages. *Autophagy* 12: 1240–1258
- Pastore N, Vainshtein A, Klisch TJ, Armani A, Huynh T, Herz NJ, Polishchuk EV, Sandri M & Ballabio A (2017) TFE3 regulates whole-body energy metabolism in cooperation with TFEB. *EMBO Mol Med* 9: 605–621
- Roczniak-Ferguson A, Petit CS, Froehlich F, Qian S, Ky J, Angarola B, Walther TC & Ferguson SM (2012) The transcription factor TFEB links mTORC1 signaling to transcriptional control of lysosome homeostasis. *Sci Signal* 5: ra42
- Schmidt LS & Linehan WM (2015) Molecular genetics and clinical features of Birt-Hogg-Dubé syndrome. *Nat Rev Urol* 12: 558–569
- Settembre C, De Cegli R, Mansueto G, Saha PK, Vetrini F, Visvikis O, Huynh T, Carissimo A, Palmer D, Klisch TJ, *et al* (2013) TFEB controls cellular lipid metabolism through a starvation-induced autoregulatory loop. *Nat Cell Biol* 15: 647–658
- Steingrimsson E, Tessarollo L, Pathak B, Hou L, Arnheiter H, Copeland NG & Jenkins NA (2002) Mitf and Tfe3, two members of the Mitf-Tfe family of bHLH-Zip transcription factors, have important but functionally redundant roles in osteoclast development. *Proc Natl Acad Sci USA* 99: 4477–4482
- Yang Y, Padilla-Nash HM, Vira MA, Abu-Asab MS, Val D, Worrell R, Tsokos M, Merino MJ, Pavlovich CP, Ried T, *et al* (2008) The UOK 257 cell line: a novel model for studies of the human Birt-Hogg-Dubé gene pathway. *Cancer Genet Cytogenet* 180: 100–109

15th Feb 2023

Dear Andrea,

Thank you for sending us your revised manuscript. We have now received the comments from the two referees who agreed to re-review your manuscript. As you will see below, the referees are satisfied with the modifications and think the study is now suitable for publication.

Before we can formally accept your manuscript, we would ask you to address the following editorial-level issues:

Kind regards,
Jingyi

Jingyi Hou
Editor
EMBO Molecular Medicine

*** Instructions to submit your revised manuscript ***

*** PLEASE NOTE *** As part of the EMBO Publications transparent editorial process initiative (see our Editorial at https://www.embopress.org/doi/pdf/10.1002/emmm.201000094), EMBO Molecular Medicine will publish online a Review Process File to accompany accepted manuscripts.

In the event of acceptance, this file will be published in conjunction with your paper and will include the anonymous referee reports, your point-by-point response and all pertinent correspondence relating to the manuscript. If you do NOT want this file to be published, please inform the editorial office at contact@embomolmed.org.

To submit your manuscript, please follow this link:

https://embomolmed.msubmit.net/cgi-bin/main.plex

When submitting your revised manuscript, please include:

1) a .docx formatted version of the manuscript text (including Figure legends and tables)

2) Separate figure files*

3) supplemental information as Expanded View and/or Appendix. Please carefully check the authors guidelines for formatting Expanded view and Appendix figures and tables at https://www.embopress.org/page/journal/17574684/authorguide#expandedview

4) a letter INCLUDING the reviewer's reports and your detailed responses to their comments (as Word file).

5) The paper explained: EMBO Molecular Medicine articles are accompanied by a summary of the articles to emphasize the major findings in the paper and their medical implications for the non-specialist reader. Please provide a draft summary of your article highlighting
- the medical issue you are addressing,
- the results obtained and
- their clinical impact.
This may be edited to ensure that readers understand the significance and context of the research.
Please refer to any of our published articles for an example.

6) For more information: There is space at the end of each article to list relevant web links for further consultation by our readers. Could you identify some relevant ones and provide such information as well? Some examples are patient associations, relevant databases, OMIM/proteins/genes links, author's websites, etc...

7) EMBO Molecular Medicine now requires a complete author checklist (https://www.embopress.org/page/journal/17574684/authorguide) to be submitted with all revised manuscripts. Please use the checklist as guideline for the sort of information we need WITHIN the manuscript. The checklist should only be filled with page

numbers were the information can be found. This is particularly important for animal reporting, antibody dilutions (missing) and exact values and n that should be indicted instead of a range.

8) Every published paper now includes a 'Synopsis' to further enhance discoverability. Synopses are displayed on the journal webpage and are freely accessible to all readers. They include a short stand first (maximum of 300 characters, including space) as well as 2-5 one sentence bullet points that summarise the paper. Please write the bullet points to summarise the key NEW findings. They should be designed to be complementary to the abstract - i.e. not repeat the same text. We encourage inclusion of key acronyms and quantitative information (maximum of 30 words / bullet point). Please use the passive voice. Please attach these in a separate file or send them by email, we will incorporate them accordingly.

You are also welcome to suggest a striking image or visual abstract to illustrate your article. If you do please provide a jpeg file 550 px-wide x 400-px high.

9) A Conflict of Interest statement should be provided in the main text

10) Please note that we now mandate that all corresponding authors list an ORCID digital identifier. This takes <90 seconds to complete. We encourage all authors to supply an ORCID identifier, which will be linked to their name for unambiguous name identification.

Currently, our records indicate that the ORCID for your account is 0000-0003-1381-4604.

Please click the link below to modify this ORCID:
Link Not Available

11) The system will prompt you to fill in your funding and payment information. This will allow Wiley to send you a quote for the article processing charge (APC) in case of acceptance. This quote takes into account any reduction or fee waivers that you may be eligible for. Authors do not need to pay any fees before their manuscript is accepted and transferred to our publisher.

*Additional important information regarding Figures

Each figure should be given in a separate file and should have the following resolution:
Graphs 800-1,200 DPI
Photos 400-800 DPI
Colour (only CMYK) 300-400 DPI"

Figures are not edited by the production team. All lettering should be the same size and style; figure panels should be indicated by capital letters (A, B, C etc). Gridlines are not allowed except for log plots. Figures should be numbered in the order of their appearance in the text with Arabic numerals. Each Figure must have a separate legend and a caption is needed for each panel.

*Additional important information regarding figures and illustrations can be found at
https://bit.ly/EMBOPressFigurePreparationGuideline. See also figure legend preparation guidelines:
https://www.embopress.org/page/journal/17574684/authorguide#figureformat

The system will prompt you to fill in your funding and payment information. This will allow Wiley to send you a quote for the article processing charge (APC) in case of acceptance. This quote takes into account any reduction or fee waivers that you may be eligible for. Authors do not need to pay any fees before their manuscript is accepted and transferred to our publisher.

***** Reviewer's comments *****

Referee #1 (Comments on Novelty/Model System for Author):

No further concerns

Referee #1 (Remarks for Author):

The Authors have properly addressed the concerns raised by the Reviewer

Referee #2 (Remarks for Author):

The authors have explained the issues I raised, and I have no further questions about publishing the study.

28th Feb 2023

Dear Andrea,

I am pleased to inform you that your manuscript is accepted for publication and is now being sent to our publisher to be included in the next available issue of EMBO Molecular Medicine.

We would like to remind you that as part of the EMBO Publications transparent editorial process initiative, EMBO Molecular Medicine will publish a Review Process File online to accompany accepted manuscripts. If you do NOT want the file to be published or would like to exclude figures, please immediately inform the editorial office via e-mail.

Please read below for additional IMPORTANT information regarding your article, its publication and the production process.

Congratulations on your interesting work.

Best wishes,
Jingyi

Jingyi Hou
Editor
EMBO Molecular Medicine

Follow us on Twitter @EmboMolMed
Sign up for eTOCs at embopress.org/alertsfeeds

*** *** *** IMPORTANT INFORMATION *** *** ***

SPEED OF PUBLICATION
The journal aims for rapid publication of papers, using using the advance online publication "Early View" to expedite the process: A properly copy-edited and formatted version will be published as "Early View" after the proofs have been corrected. Please help the Editors and publisher avoid delays by providing e-mail address(es), telephone and fax numbers at which author(s) can be contacted.

Should you be planning a Press Release on your article, please get in contact with embomolmed@wiley.com as early as possible, in order to coordinate publication and release dates.

LICENSE AND PAYMENT:

All articles published in EMBO Molecular Medicine are fully open access: immediately and freely available to read, download and share.

EMBO Molecular Medicine charges an article processing charge (APC) to cover the publication costs. You, as the corresponding author for this manuscript, should have already received a quote with the article processing fee separately. Please let us know in case this quote has not been received.

Once your article is at Wiley for editorial production you will receive an email from Wiley's Author Services system, which will ask you to log in and will present you with the publication license form for completion. Within the same system the publication fee can be paid by credit card, an invoice, pro forma invoice or purchase order can be requested.

Payment of the publication charge and the signed Open Access Agreement form must be received before the article can be published online.

PROOFS

You will receive the proofs by e-mail approximately 2 weeks after all relevant files have been sent o our Production Office.

Please return them within 48 hours and if there should be any problems, please contact the production office at embopressproduction@wiley.com.

Please inform us if there is likely to be any difficulty in reaching you at the above address at that time. Failure to meet our deadlines may result in a delay of publication.

All further communications concerning your paper proofs should quote reference number EMM-2022-16877-V3 and be directed to the production office at embopressproduction@wiley.com.

Thank you,

Jingyi Hou
Editor
EMBO Molecular Medicine

EMBO Press Author Checklist

Corresponding Author Name: Chiara Di Malta, W Marston Linehan and Andrea Ballabio
Journal Submitted to: Embo Molecular Medicine
Manuscript Number: EMM-2022-16877

USEFUL LINKS FOR COMPLETING THIS FORM
The EMBO Journal - Author Guidelines
EMBO Reports - Author Guidelines
Molecular Systems Biology - Author Guidelines
EMBO Molecular Medicine - Author Guidelines

Reporting Checklist for Life Science Articles (updated January 2022)
This checklist is adapted from Materials Design Analysis Reporting (MDAR) Checklist for Authors. MDAR establishes a minimum set of requirements in transparent reporting in the life sciences (see Statement of Task: 10.31222/osf.io/9sm4x). Please follow the journal's guidelines in preparing your manuscript.
Please note that a copy of this checklist will be published alongside your article.

Abridged guidelines for figures
1. Data
The data shown in figures should satisfy the following conditions:
- the data were obtained and processed according to the field's best practice and are presented to reflect the results of the experiments in an accurate and unbiased manner.
- ideally, figure panels should include only measurements that are directly comparable to each other and obtained with the same assay.
- plots include clearly labeled error bars for independent experiments and sample sizes. Unless justified, error bars should not be shown for technical replicates.
- if n<5, the individual data points from each experiment should be plotted. Any statistical test employed should be justified.
- Source Data should be included to report the data underlying figures according to the guidelines set out in the authorship guidelines on Data Presentation.

2. Captions
Each figure caption should contain the following information, for each panel where they are relevant:
- a specification of the experimental system investigated (eg cell line, species name).
- the assay(s) and method(s) used to carry out the reported observations and measurements.
- an explicit mention of the biological and chemical entity(ies) that are being measured.
- an explicit mention of the biological and chemical entity(ies) that are altered/varied/perturbed in a controlled manner.
- the exact sample size (n) for each experimental group/condition, given as a number, not a range;
- a description of the sample collection allowing the reader to understand whether the samples represent technical or biological replicates (including how many animals, litters, cultures, etc.).
- a statement of how many times the experiment shown was independently replicated in the laboratory.
- definitions of statistical methods and measures:
  - common tests, such as t-test (please specify whether paired vs. unpaired), simple χ2 tests, Wilcoxon and Mann-Whitney tests, can be unambiguously identified by name only, but more complex techniques should be described in the methods section;
  - are tests one-sided or two-sided?
  - are there adjustments for multiple comparisons?
  - exact statistical test results, e.g., P values = x but not P values < x;
  - definition of 'center values' as median or average;
  - definition of error bars as s.d. or s.e.m.

Please complete ALL of the questions below.
Select "Not Applicable" only when the requested information is not relevant for your study.

Materials

Newly Created Materials	Information included in the manuscript?	In which section is the information available? (Reagents and Tools Table, Materials and Methods, Figures, Data Availability Section)
New materials and reagents need to be available; do any restrictions apply?	Not Applicable	

Antibodies	Information included in the manuscript?	In which section is the information available? (Reagents and Tools Table, Materials and Methods, Figures, Data Availability Section)
For **antibodies** provide the following information:<br>- Commercial antibodies: RRID (if possible) or supplier name, catalogue number and or/clone number<br>- Non-commercial: RRID or citation	Yes	Materials and Methods

DNA and RNA sequences	Information included in the manuscript?	In which section is the information available? (Reagents and Tools Table, Materials and Methods, Figures, Data Availability Section)
**Short novel DNA or RNA including primers, probes**: provide the sequences.	Not Applicable	

Cell materials	Information included in the manuscript?	In which section is the information available? (Reagents and Tools Table, Materials and Methods, Figures, Data Availability Section)
**Cell lines:** Provide species information, strain. Provide accession number in repository **OR** supplier name, catalog number, clone number, and/**OR** RRID.	Yes	Materials and Methods
**Primary cultures:** Provide species, strain, sex of origin, genetic modification status.	Not Applicable	
Report if the cell lines were recently **authenticated** (e.g., by STR profiling) and tested for mycoplasma contamination.	Not Applicable	

Experimental animals	Information included in the manuscript?	In which section is the information available? (Reagents and Tools Table, Materials and Methods, Figures, Data Availability Section)
**Laboratory animals or Model organisms:** Provide species, strain, sex, age, genetic modification status. Provide accession number in repository **OR** supplier name, catalog number, clone number, **OR** RRID.	Yes	Materials and Methods
**Animal observed in or captured from the field:** Provide species, sex, and age where possible.	Not Applicable	
Please detail **housing and husbandry conditions**.	Yes	Materials and Methods

Plants and microbes	Information included in the manuscript?	In which section is the information available? (Reagents and Tools Table, Materials and Methods, Figures, Data Availability Section)
**Plants:** provide species and strain, ecotype and cultivar where relevant, unique accession number if available, and source (including location for collected wild specimens).	Not Applicable	
**Microbes:** provide species and strain, unique accession number if available, and source.	Not Applicable	

Human research participants	Information included in the manuscript?	In which section is the information available? (Reagents and Tools Table, Materials and Methods, Figures, Data Availability Section)
If collected and within the bounds of privacy constraints report on age, sex and gender or ethnicity for all study participants.	Yes	Materials and Methods and Table EV2

Core facilities	Information included in the manuscript?	In which section is the information available? (Reagents and Tools Table, Materials and Methods, Figures, Data Availability Section)
If your work benefited from core facilities, was their service mentioned in the acknowledgments section?	Yes	Acknowledgments section

Design

Study protocol	Information included in the manuscript?	In which section is the information available? (Reagents and Tools Table, Materials and Methods, Figures, Data Availability Section)
If study protocol has been **pre-registered, provide DOI in the manuscript**. For clinical trials, provide the trial registration number **OR** cite DOI.	Not Applicable	
Report the **clinical trial registration number** (at ClinicalTrials.gov or equivalent), where applicable.	Not Applicable	

Laboratory protocol	Information included in the manuscript?	In which section is the information available? (Reagents and Tools Table, Materials and Methods, Figures, Data Availability Section)
Provide DOI OR other citation details if **external detailed step-by-step protocols** are available.	Not Applicable	

Experimental study design and statistics	Information included in the manuscript?	In which section is the information available? (Reagents and Tools Table, Materials and Methods, Figures, Data Availability Section)
Include a statement about **sample size** estimate even if no statistical methods were used.	Yes	Figure legends
Were any steps taken to minimize the effects of subjective bias when allocating animals/samples to treatment (e.g. **randomization procedure**)? If yes, have they been described?	Yes	Materials and Methods
Include a statement about **blinding** even if no blinding was done.	Yes	Materials and Methods
Describe **inclusion/exclusion criteria** if samples or animals were excluded from the analysis. Were the criteria pre-established?<br><br>If sample or data points were omitted from analysis, report if this was due to attrition or intentional exclusion and provide justification.	Not Applicable	no samples/animals were excluded
For every figure, are **statistical tests** justified as appropriate? Do the data meet the assumptions of the tests (e.g., normal distribution)? Describe any methods used to assess it. Is there an estimate of variation within each group of data? Is the variance similar between the groups that are being statistically compared?	Yes	Materials and Methods and Figure legends

Sample definition and in-laboratory replication	Information included in the manuscript?	In which section is the information available? (Reagents and Tools Table, Materials and Methods, Figures, Data Availability Section)
In the figure legends: state number of times the experiment was **replicated** in laboratory.	Yes	Figure legends
In the figure legends: define whether data describe **technical or biological replicates**.	Yes	Figure legends

Ethics

Ethics	Information included in the manuscript?	In which section is the information available? (Reagents and Tools Table, Materials and Methods, Figures, Data Availability Section)
Studies involving **human participants**: State details of **authority granting ethics approval** (IRB or equivalent committee(s), provide reference number for approval.	Yes	Materials and Methods
Studies involving **human participants**: Include a statement confirming that **informed consent** was obtained from all subjects and that the experiments conformed to the principles set out in the WMA Declaration of Helsinki and the Department of Health and Human Services Belmont Report.	Yes	Materials and Methods
Studies involving **human participants:** For publication of **patient photos**, include a statement confirming that consent to publish was obtained.	Yes	Materials and Methods
Studies involving experimental **animals**: State details of **authority granting ethics approval** (IRB or equivalent committee(s), provide reference number for approval. Include a statement of compliance with ethical regulations.	Yes	Materials and Methods
Studies involving **specimen and field samples:** State if relevant **permits** obtained, provide details of authority approving study; if none were required, explain why.	Not Applicable	

Dual Use Research of Concern (DURC)	Information included in the manuscript?	In which section is the information available? (Reagents and Tools Table, Materials and Methods, Figures, Data Availability Section)
Could your study fall under dual use research restrictions? Please check biosecurity documents and list of **select agents and toxins** (CDC): https://www.selectagents.gov/sat/list.htm	Not Applicable	
If you used a select agent, is the security level of the lab appropriate and reported in the manuscript?	Not Applicable	
If a study is subject to dual use research of concern regulations, is the name of the **authority granting approval and reference number** for the regulatory approval provided in the manuscript?	Not Applicable	

Reporting

The MDAR framework recommends adoption of discipline-specific guidelines, established and endorsed through community initiatives. Journals have their own policy about requiring specific guidelines and recommendations to complement MDAR.

Adherence to community standards	Information included in the manuscript?	In which section is the information available? (Reagents and Tools Table, Materials and Methods, Figures, Data Availability Section)
State if relevant guidelines or checklists (e.g., **ICMJE, MIBBI, ARRIVE, PRISMA**) have been followed or provided.	Not Applicable	
For **tumor marker prognostic studies**, we recommend that you follow the **REMARK** reporting guidelines (see link list at top right). See author guidelines, under 'Reporting Guidelines'. Please confirm you have followed these guidelines.	Not Applicable	
For **phase II and III randomized controlled trials**, please refer to the **CONSORT** flow diagram (see link list at top right) and submit the CONSORT checklist (see link list at top right) with your submission. See author guidelines, under 'Reporting Guidelines'. Please confirm you have submitted this list.	Not Applicable	

Data Availability

Data availability	Information included in the manuscript?	In which section is the information available? (Reagents and Tools Table, Materials and Methods, Figures, Data Availability Section)
Have **primary datasets** been deposited according to the journal's guidelines (see 'Data Deposition' section) and the respective accession numbers provided in the Data Availability Section?	Yes	Materials and Methods
Were **human clinical and genomic datasets** deposited in a public access-controlled repository in accordance to ethical obligations to the patients and to the applicable consent agreement?	Yes	Materials and Methods
Are **computational models** that are central and integral to a study available without restrictions in a machine-readable form? Were the relevant accession numbers or links provided?	Not Applicable	
If publicly available data were reused, provide the respective **data citations in the reference list**.	Not Applicable	
